# Epigenetic tumor heterogeneity in the era of single-cell profiling with nanopore sequencing

**DOI:** 10.1186/s13148-022-01323-6

**Published:** 2022-08-27

**Authors:** Yohannis Wondwosen Ahmed, Berhan Ababaw Alemu, Sisay Addisu Bekele, Solomon Tebeje Gizaw, Muluken Fekadie Zerihun, Endriyas Kelta Wabalo, Maria Degef Teklemariam, Tsehayneh Kelemu Mihrete, Endris Yibru Hanurry, Tensae Gebru Amogne, Assaye Desalegne Gebrehiwot, Tamirat Nida Berga, Ebsitu Abate Haile, Dessiet Oma Edo, Bizuwork Derebew Alemu

**Affiliations:** 1 Department of Medical Biochemistry, School of Medicine, College of Health Sciences, Addis Ababa University, P.O. Box: 9086, Addis Ababa, Ethiopia; 2 Department of Medical Biochemistry, School of Medicine, St. Paul’s Hospital, Millennium Medical College, Addis Ababa, Ethiopia; 3 Department of Medical Anatomy, School of Medicine, College of Health Sciences, Addis Ababa University, Addis Ababa, Ethiopia; 4 Department of Statistics, College of Natural and Computational Sciences, Mizan Tepi University, Tepi, Ethiopia

**Keywords:** Epigenetics, Nanopore sequencing, CpG island, Tumor heterogeneity, Methylated cytosine, Oxford nanopore, MinION

## Abstract

Nanopore sequencing has brought the technology to the next generation in the science of sequencing. This is achieved through research advancing on: pore efficiency, creating mechanisms to control DNA translocation, enhancing signal-to-noise ratio, and expanding to long-read ranges. Heterogeneity regarding epigenetics would be broad as mutations in the epigenome are sensitive to cause new challenges in cancer research. Epigenetic enzymes which catalyze DNA methylation and histone modification are dysregulated in cancer cells and cause numerous heterogeneous clones to evolve. Detection of this heterogeneity in these clones plays an indispensable role in the treatment of various cancer types. With single-cell profiling, the nanopore sequencing technology could provide a simple sequence at long reads and is expected to be used soon at the bedside or doctor’s office. Here, we review the advancements of nanopore sequencing and its use in the detection of epigenetic heterogeneity in cancer.

## Introduction

At the half of the twentieth century, the discovery of DNA structure brought the demand to sequence it [1–3]. The two most popular methods Sanger and Maxam–Gilbert were introduced based on chain termination reactions and chemical cleavage analysis, respectively [4–7]. The Sanger method which depends on termination of the growing nucleotide chain with dideoxythymidine triphosphate (ddTTP) dominated the traditional Maxam–Gilbert method [8, 9, 32, 33]. It was also used by automation and mass in the human genome project (HGP) [10–12]. Due to technological shortcomings, the human genome was not possible to be fully sequenced in 2003 [13, 14]. Recently, the gapless next-generation sequencing (NGS) in a T2T consortium makes it possible to address the whole-genome parts. NGS is one of the sequencing technologies that made possible the advance in Oxford Nanopore sequencing with ultra-long-read capacities [15, 16].

Pocket-sized nanopore sequencers, which do not need a reverse transcription process and do not require a high-skill data entry approach, are becoming in need following their introduction for commercial purposes in 2014 [17, 18]. The technology enabled viral genome sequencing during the outbreaks of the Ebola virus in remote areas of West Africa and the Zika virus in the deeply forested regions of Brazil [19, 20]. These days it is used in China to sequence and identify SARS-CoV-2 [21, 22].

Single-molecule direct sequencing characteristics of nanopore-based sequencing methods look tailor-made to sequence epigenetics which has a significant role in driving cancer and its heterogeneity [23–25]. Methyl-CpG-binding proteins are identifiers of methylcytosine residues to attract transcriptional repressor complexes like histone deacetylases (HDAC). Those proteins connecting methylation with histone modification are the foundations of epigenetics [26, 27]. Here, we review an introduction to the development of NGS technology based on nanopore sequencing and its application to identify epigenetic tumor heterogeneity [28]. We also discuss the most studied and more impactful methylation and related cytosine modifications that exist as CpG islands.

## Advancement of nanopore sequencing as the 4th-generation sequencer

### Sequencing from Sanger to 4th-generation NGS

DNA sequencing technology has passed through a half-century of advancements starting from the Sanger and Maxam–Gilbert to the fourth generation of NGS, and nanopore sequencing is marked as the beginning of the fourth generation of gene sequencing technology [29–31].

**Fig. 1:**
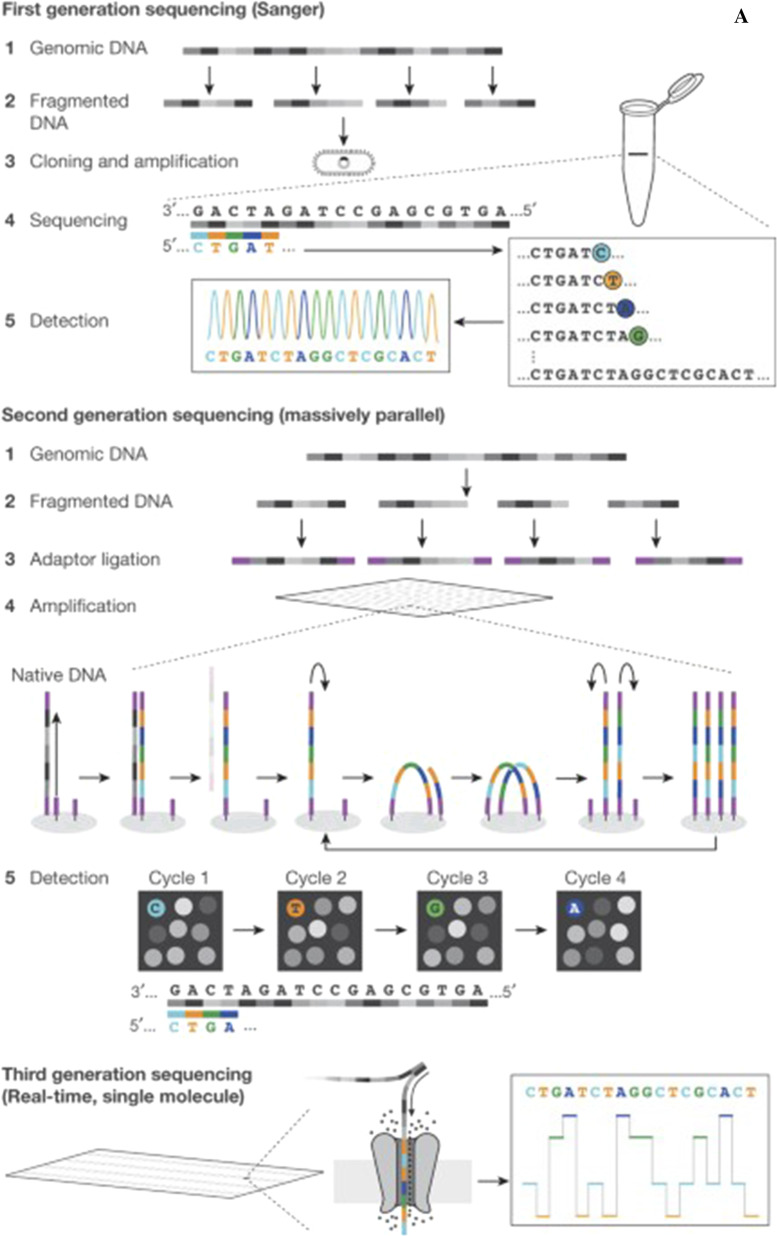

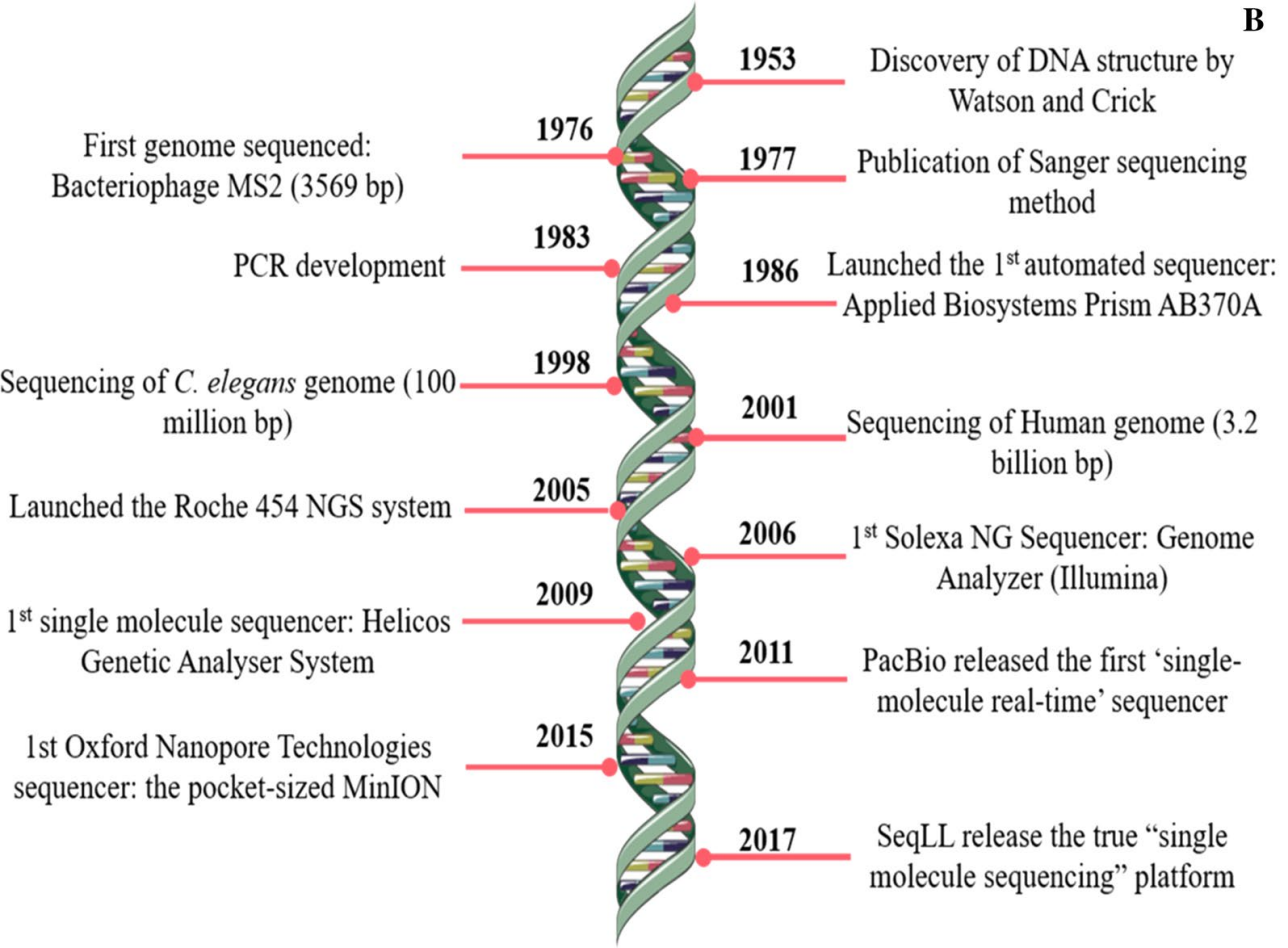
**A** Diagrammatic examples of first, second, and third-generation sequencing. Image reprinted from [48] with permission of the publisher (Request ID 600061564, 25 Nov 2021). **B** DNA sequencing timeline. The landmark events in DNA sequencing. Image adapted from [49]

HGP when started in 1990 needed to have a well-established sequencing technology that would make the project feasible because of the automation of the sequencing technology and the scaling up of some advancements [34, 35]. Finalization of HGP brought the reference human genome sequence as well as the advancement of the sequencing technology too [10, 31]. First-generation sequencing used for HGP required longer running times and high cost with limited throughputs. As sequencing demanded more throughput and low-cost technology, the shift from the first generation to the second generation was made in the mid-20s to establish the second-generation sequencing (SGS) [31, 36–38]. The shifting was achieved by devising a massively parallel sequencing system that started with the introduction of Roche 454’s pyrosequencing [39–41]. Since SGS is limited to short-read (35-1000 bases) and requires PCR amplification like Sanger’s method, it is unable to read regions such as high/low G + C regions, tandem repeat regions, interspersed repeat regions, and is hard to sequence [36, 38]. These SGS difficulties in resolving repetitive sequences of highly fragmented assemblies lead to the development of the next era of gene sequencing, third-generation sequencings (TGSs) including Illumina/Solexa and PacBio [44–46]. TGS is marked by single-molecule real-time (SMRT) sequencing, with improved reading length from tens of bases to tens of thousands of bases, reduced sequencing time from days to hours, and PCR elimination of sequencing biases [44, 47] (Fig. 1).

In 2007, Illumina/Solexa was introduced the sequencing by synthesis (SBS) method of Genome Analyzer platform afterward sequencing by ligation system of ABI’s SOLID—Applied Biosystems instrument [42, 43]. SBS with bisulfite sequencing could be used to identify the methylation of cytosine. However, it could not be able to discriminate between C and 5mC from 5hmC [59, 60].

PacBio RS II as the first commercialized third-generation DNA sequencers that works by enabling the direct observation of DNA synthesis has the advantage of sequencing long-read lengths, high consensus accuracy, a low degree of bias, simultaneous capability of epigenetic characterization and is useful for direct detection of base modifications such as methylation [36, 38, 44, 47, 50, 54–56]. Generally, PacBio RS II is ideal for whole-genome sequencing, targeted sequencing, complex population analysis, RNA sequencing, and epigenetic characterization. PacBio RS II works without PCR amplification and offers the advantages of providing long-read lengths (> 20 kb) and maximum read length (> 60 kb) over first and second-generation platforms. PacBio system is also capable of directly detecting and discriminating epigenetic modifications [28, 54]. Moreover, many hybrid sequencing strategies have been developed and coupled with PacBio to make it more affordable and scalable. The noticeable limitations of PacBio include lower throughput, higher error rates, and higher cost per base [51–53].

**Fig. 2:**
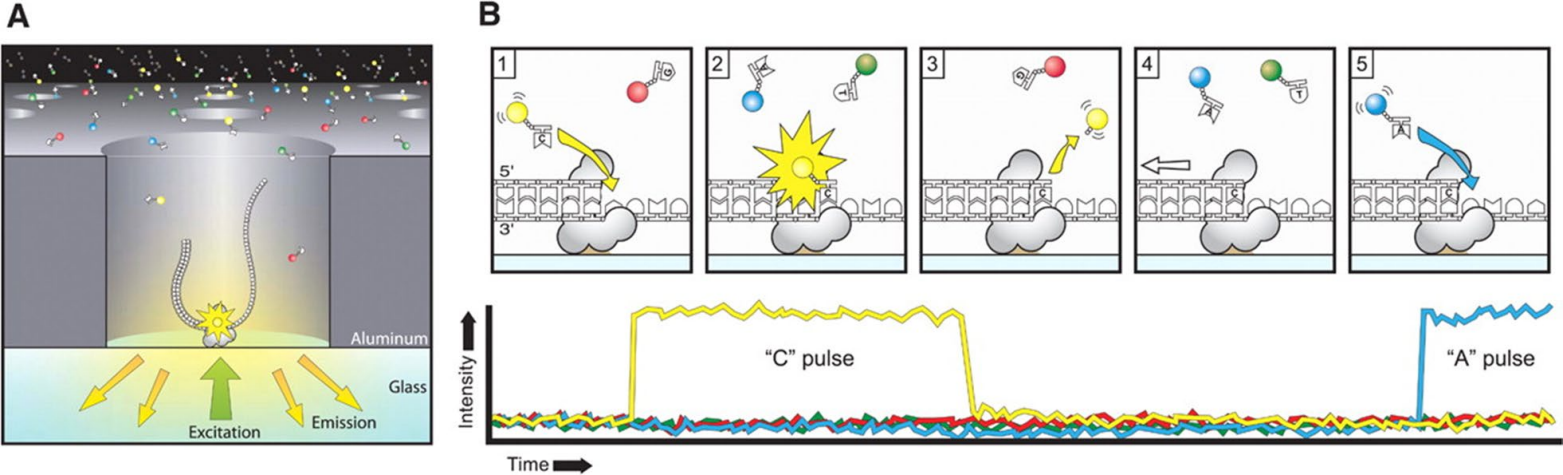
Principle of single-molecule real-time sequencing. **A** A single molecule of DNA template-bound Phi 29 is immobilized at the bottom of the zero-mode waveguide nanophotonic structure, which is illuminated by laser light. **B** Diagrammatic order of the phospho-linked dNTP association cycle. (1) Phospho-linked nucleotide forms a binding with a template in the polymerase active site. (2) Advancing fluorescence output on the analogous color channel. (3) Phosphodiester linkage formation releases the dye binder phosphate product followed by the ending of zero-mode waveguide nanophotonic fluorescence pulse. (4) Translocation of polymerase enzyme to the next nucleotide of the template strand. (5) Binding of the next cognate nucleotide on the active site of polymerase to continue the cycle [57, 58]. Image reprinted from [57] with permission of the publisher (Order license ID: 1164215-1, 25 Nov 2021)

In PacBio single nucleotide sequencing, four fluorescent-labeled nucleotides with distinct emission spectrum are added to the chip called SMRT cell, and a zero-mode wavelength light pulse is captured when a base is added (Fig. 2). The pulse is then interpreted as a base sequence [38, 54].

The most recent (NGS) sequencing with nanopore technology (majorly discussed in this review) has a thin membrane structure that holds nanoscale holes. When biological molecules smaller than the nanopore pass through the hole, it detects the potential charge of individual molecules passing through it [31, 61, 62]. The four various companies are competing to dominate the NGS market based on their price, method, and average reading length (Table 1).

**Table 1:** Comparison of different NGS technologies. Adapted from [36]

	Roche 454	Illumina/Solexa genome analyzer	PacBio SMRT	MinION
Company	Roche	Illumina	Pacific Biosciences	Oxford Nanopore Technologies (ONT)
Release year	2005	2006/7	2010	2014
Method	Sequencing by synthesis	Sequencing by synthesis	Sequencing by synthesis	Direct sequencing
Price	$13,700	Expensive—$ 49,000 to over a million	$695	$1000 with 500 disposable flow cells
Average reads	100–150 bp	75 bp highly accurate	10-25 Kb	The longest reads over 100 Kb

### The development of Oxford Nanopore sequencing technology

In 2012, nanopore technology started to be applicable for RNA sequencing with reverse transcription and amplification methods. Following that, Oxford Nanopore Technologies (ONT) developed a device based on an array of biological nanopores that enable reliable decoding of long sequences with an acceptable error rate, low cost, and better miniaturization [64, 94]. Its long-read sequencing capacity makes it a landmark in the history of sequencing [63–66]. The sequencing is a direct, highly parallel, real-time, single-molecule method that manifests an improved reading length of nucleotides [95–98].

Nanopores in NGS could reduce the time required for sample amplification along with enzymes, reagents, and optics used in sequencing by synthesis methods. Nanopore sensors are purely electrical and could penetrate blood or saliva DNA samples [67–70]. A nanopore is a nanoscale opening biological pore simulated from a protein channel through a lipid membrane. The pore can be made by ion track etching or straightforward planar lithography. Using a sensitive patch-clamp amplifier, the ionic current through a single pore can be used to separate two chambers labeled cis and trans [71–73]. Voltage is also applied across the membrane to create an ionic current through the nanopore [67, 72].

**Fig. 3:**
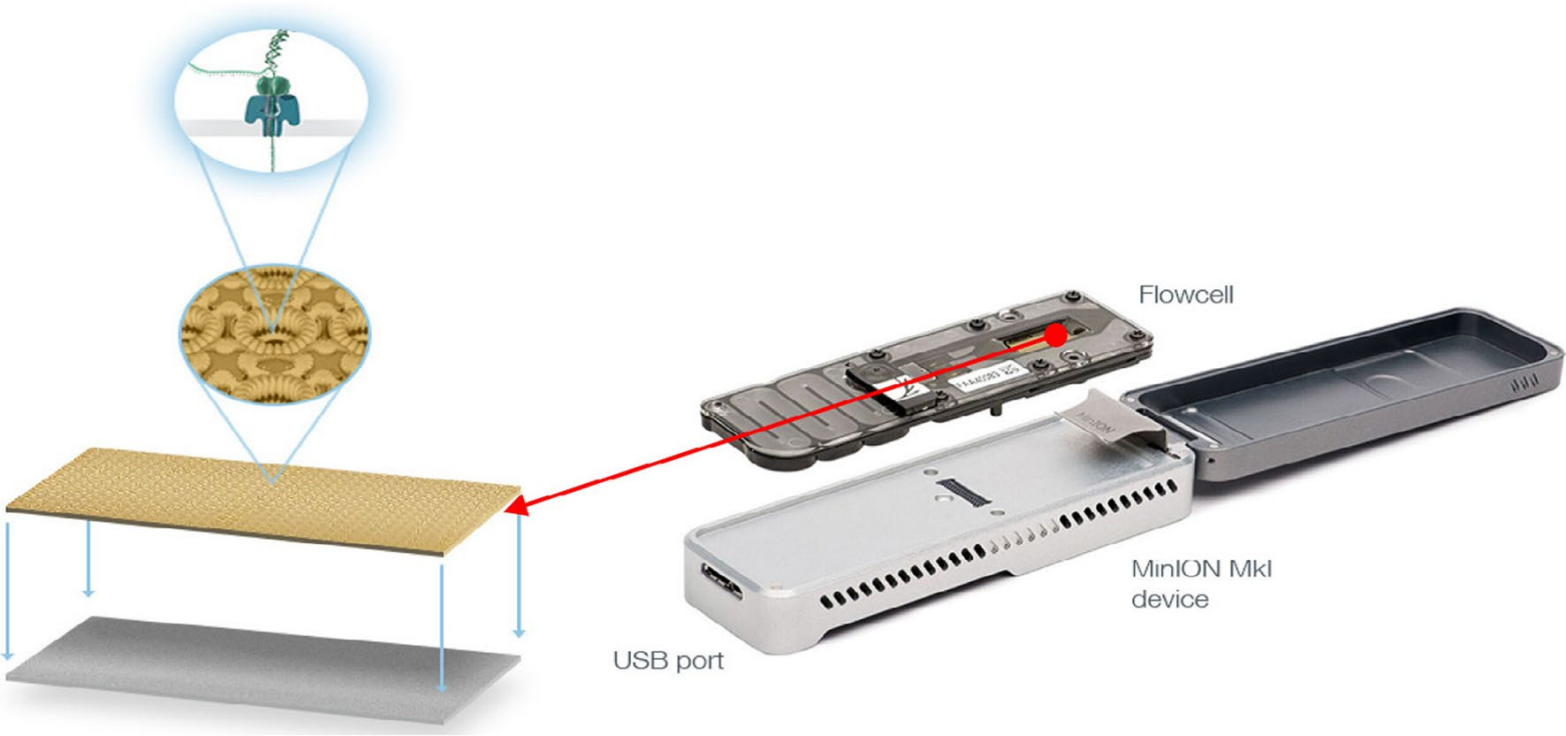
The MinION sequencing device—DNA sequencing is performed by adding a sample to the flow cell. The sensor measures the change in magnitude of current in the nanopore when the DNA molecule passes through it. The data streams are passed to the application-specific integrated circuit ASIC and MinKnow, which generate the signal-level data. Image reprinted from [63] with permission of the publisher (Request ID 600062077, 01 Dec 2021)

With mandatory changes from the previous sequencing methods, nanopore sequencing is an essential tool in medicine, such as in cancer research and diagnosis [73–76]. Moreover, the pore-based sequencing can be used to sequence, assemble, and analyze structural variants and detect epigenetic marks to point-of-care implementation for future human genomics applications [75, 77–79] (Fig. 3).

Nanopore sequencing technology was the result of a combination of gradual, long, multidisciplinary efforts from different directions [80]. The first upbringing was done in 1976 when Erwin Neher and Bert Sakmann developed mechanisms to record and measure the amount of current flowing through a single ion channel embedded in a biological membrane [81, 82]. But, the direct idea to use ion current measurement for sequencing through a membrane-embedded nanopore was introduced by David Deamer in 1989 [83, 84].

**Fig. 4:**
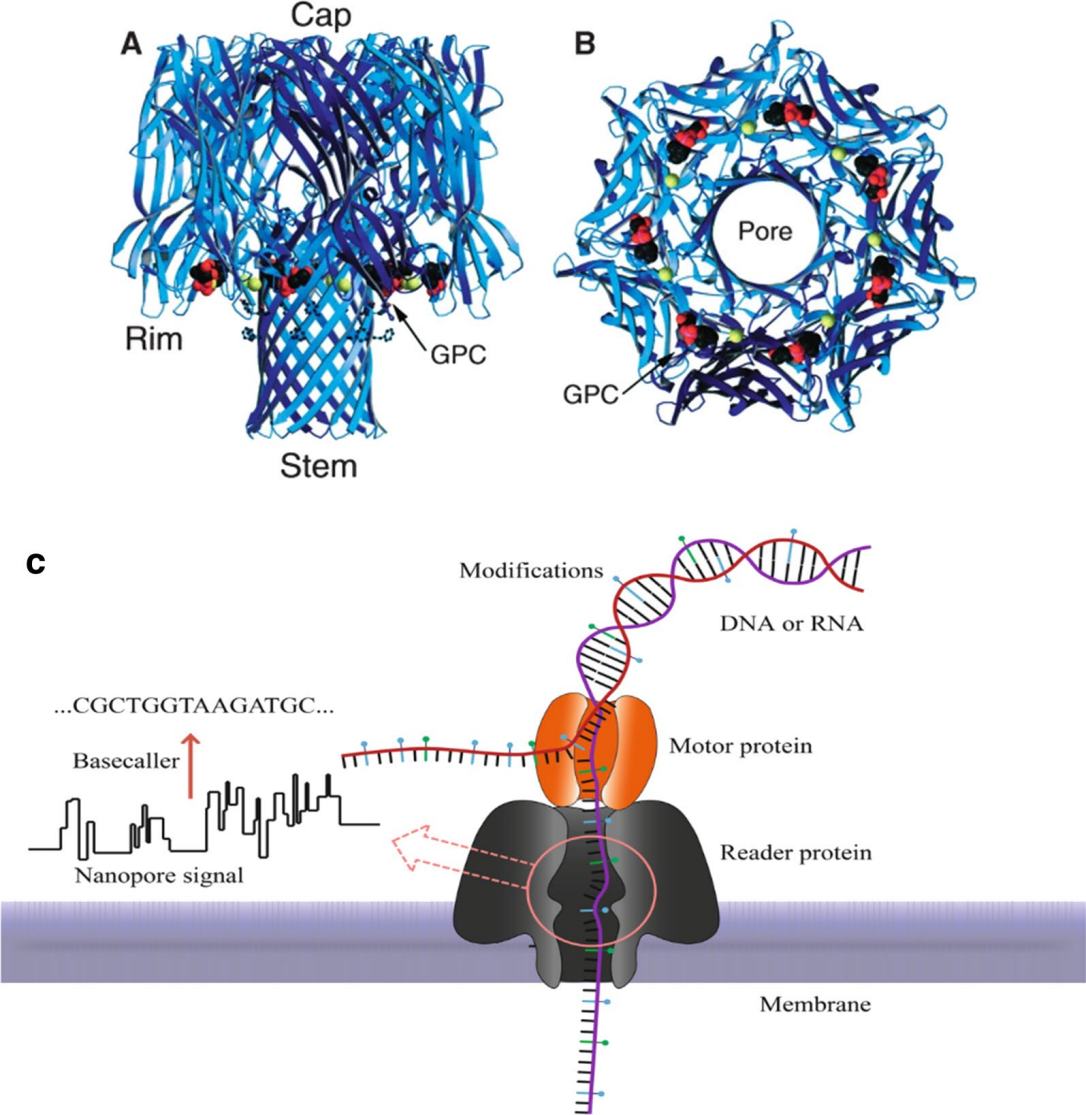
Representation of α-hemolysin from Staph aureus. Reprinted from [91] with permission of the publisher (CCC License ID: 5196920847168, 27 Nov 2021). **A** Side view of the alpha-hemolysin heptameric complex indicates the exact location of the phospholipid bilayer. **B** View of alpha-hemolysin from the cis entrance to the pore [86]. **C** Structure of α-hemolysin nanopore embedded in a phospholipid bilayer. In nanopore sequencing, the motor protein guides the DNA strand to pass through the pore. This causes current fluctuations through the membrane. The nanopore signal later is converted into a nucleic acid sequence by the base caller. The DNA substrate (violet) is inserted into the pore by an applied electric field. Image adapted from [92]

**Fig. 5:**
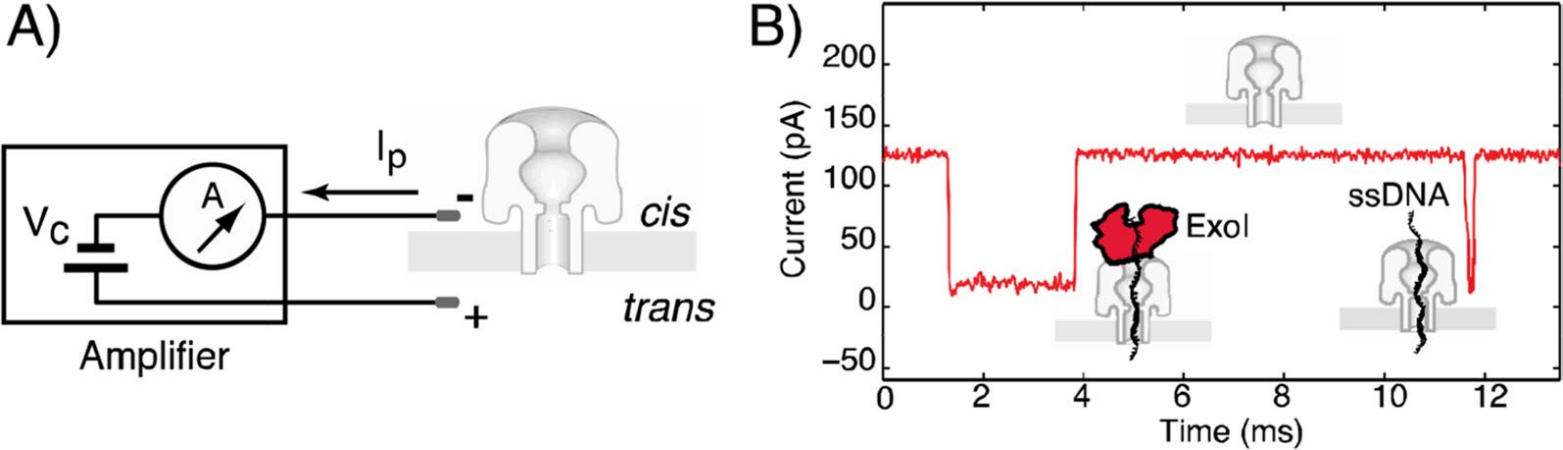
Biological nanopore instruments with representative ssDNA and protein-bound DNA events [67, 93]. Image reprinted from [67] with permission of the publisher (CCC License ID: 600061571, 25 Nov 2021). **A** The nanopore instrument uses an amplifier to apply a command voltage Vc and measure ionic current Ip through the nanopore channel. **B** At 120 mV in 1 M buffered KCl solution, 120 pA of open-channel current is attenuated to 15 pA for 0.2 ms upon the capture of ssDNA into the channel from the cis-chamber until the DNA passes through the pore. With Exol and ssDNA in the cis-chamber, bound events are also observed in which the duration of the current shift is extended 2 ms [67]

Deamer’s lack of a possible ion channel to allow a nucleotide to pass through was solved when he came across the John Kasianozicz for studying α-hemolysin, which is a protein toxin secreted by Staphylococcus aureus (Fig. 4A, B) [85, 86]. A phospholipid bilayer embedded with biological hemolysin nanopores is separated into two chambers, filled with a KCl solution. The applied electric potential with ionic current (Fig. 5) pushes the negatively charged DNA to the positive pole through the pore until it translocates (Fig. 4C) [87]. Translocation velocity depends on electrical potential applied, nanopores used, and the single or double strandedness of the DNA. Optimal velocity is around 2 nucleotides per millisecond, and a 10 × 10 array human genome can be sequenced in 8 h [88]. The four nucleotides are differentiated by various current disturbances created by translocation of ionic signal blockage. The amplitude and duration of blockages depend on the length and width of the translocating polymer [89, 90].

**Fig. 6:**
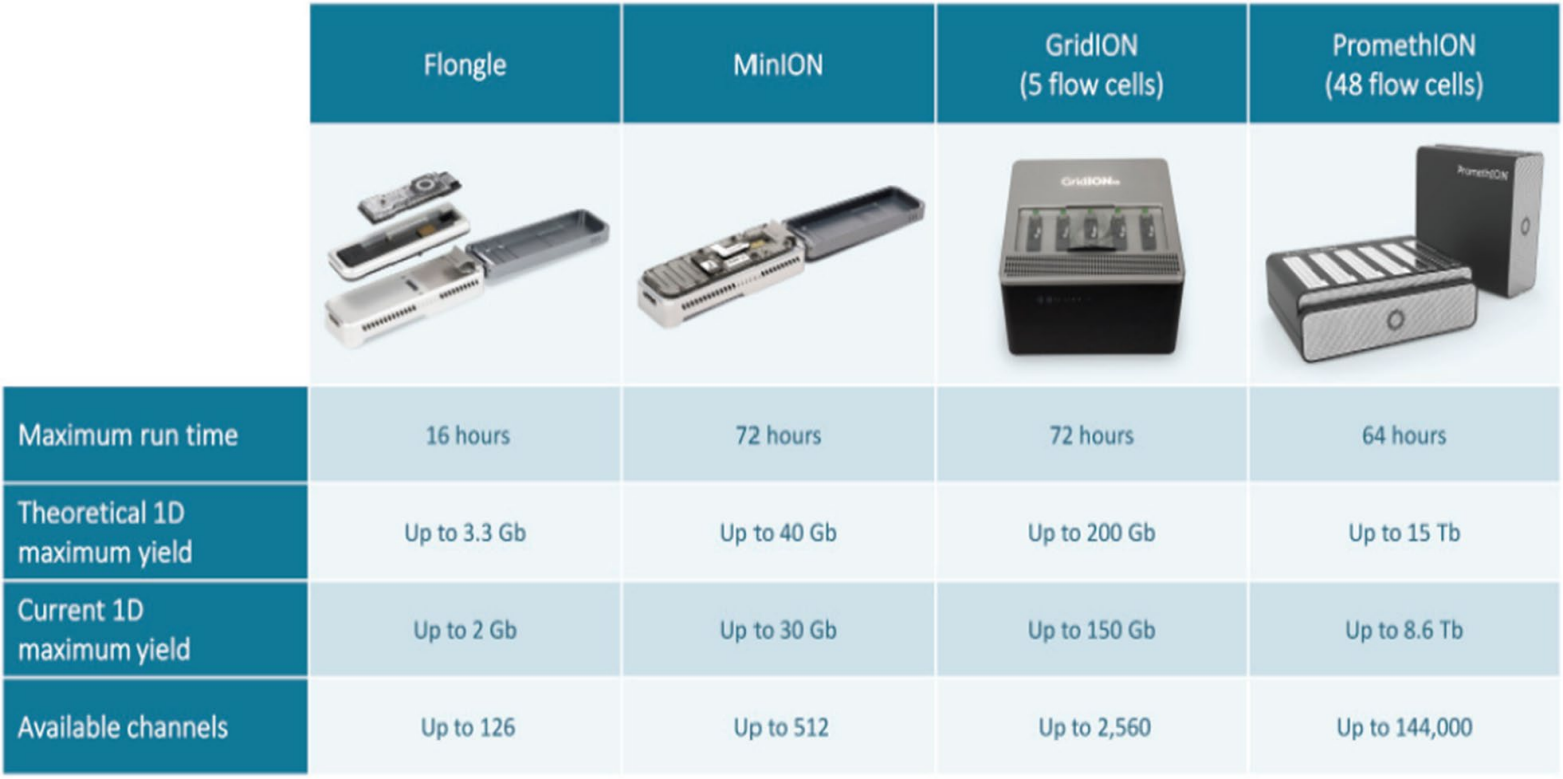
Comparison of currently available Oxford Nanopore Technologies. Adapted from [23]

To break into the sequencing by synthesis sector, ONT designed a more stable membrane to support the nanopores, which were initially manufactured from lipids. Since the lipid was extremely sensitive to pH and temperature, it was replaced by lipid-coated Teflon hand-fabricated material [99]. The usual membrane works only seconds to minutes before it collapses and takes the whole day of production of the membrane to generate half an hour of data. ONT moved on to synthetic membrane material that makes it more effective. Moreover, to overcome this challenge, in February 2012 GridION, Flongle, MinION, and PromethION platforms were displayed [100, 104, 105]. Perhaps MinION took the most attention, as it deciphers almost a billion DNA bases in 6 h while priced at $900 (Fig. 6) [102, 103].

### Biological versus solid-state nanopores

The initial biological nanopores still yielded the best results with easily makeable, highly modifiable, and reproducible structures that allow repeatable current measurement [109–111]. The inorganic nanopores have strength in terms of temperature, solvent compatibility, robustness, and the ability to be integrated with semiconductor electronics [112–114]. Solid-state nanopores have an advantage over biological counterparts such as the stronger thermal, mechanical and chemical stability; ease of modifications; tunable pore size and morphology, readily able to be integrated into nanofluidic or other nanodevices, and scalability of fabrication [115–118]. The most common solid-state nanopores are SiO_2_ and low-stress silicon-rich nitride SiN_x_. In addition to the well-developed handling of these materials for semiconductor microelectronic fabrication, silicon-based nanopores are preferred for their robustness, good resistivity, and dielectric strength [119–121]. Other elements tried for nanopores are Al_2_O_3_ and HfO_2_, to provide unique membrane fabrication [122–125].

Solid-state pores, first made by ion-beam sculpting later by transmission electron microscopy (TEM) drilling or dielectric breakdown, have the limitations of being unable to achieve the required thickness needed for membrane stability [107]. In comparison with biopores, solid-state nanopores exhibit lower single-molecule detection due to the intrinsic thickness and lack control over surface charge distribution [126].

A versatile nanopore membrane based on MoS_2_ was developed with signal amplitude five times higher than solid-state Si_3_N_4_ membranes, and unlike graphene nanopores, no special surface treatment was needed to avoid strong interactions between DNA and the surface [126, 127]. Monolayer 2D materials such as graphene, MoS_2_, WS_2,_ and hexagonal boron nitride (h-BN) are thicker as the spacing between the nucleotides [128, 129]. Compared with traditional solid-state nanopore membranes, monolayer 2D membranes are ideal for nanopore devices as they exhibit a high ionic current signal-to-noise ratio and relatively large sensing regions [129, 130]. Solid-state nanopores channels are long around 100 times the distance between two bases in a DNA molecule (0.5 nm) [131, 132]. Even though it has a sticking effect during translocation, ultrathin graphene monolayer membranes drilled by electron beams after being placed on a silicon nitride are preferable solid-state nanopore technology [131, 133].

**Fig. 7:**
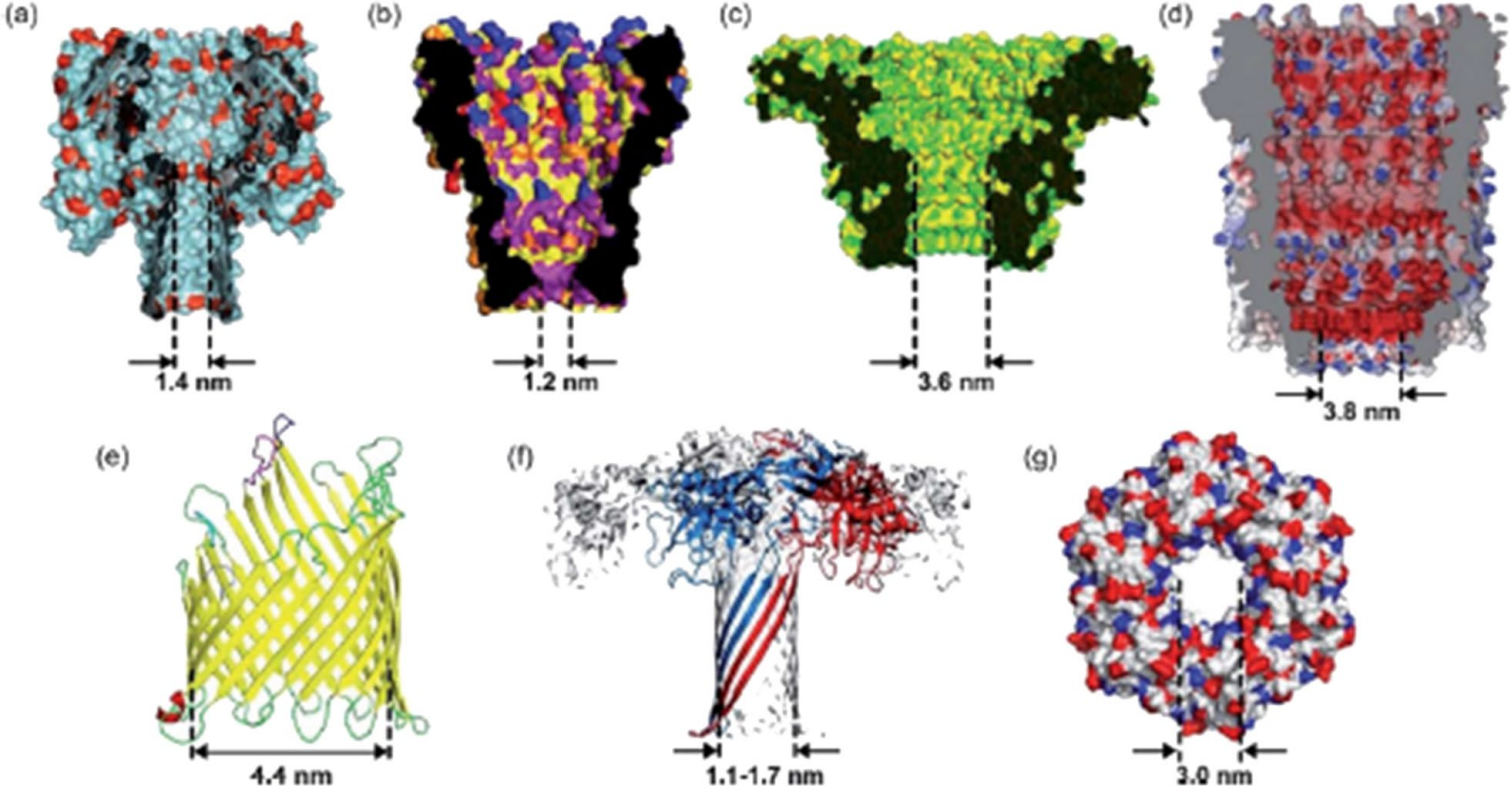
Examples of biological nanopores. **a** α-HL, **b** MspA, **c** Phi 29, **d** ClyA, **e** FhuA, **f** aerolysin, **g** SP1. Reprinted from [142] with permission of the publisher (CCC License ID: 1164219-1, 25 Nov 2021)

Following identification of hemolysin as biological pore, stable membrane nanopores allowing passage of fewer nucleotides at a time were required to reduce entry of numerous nucleotides at once [116]. Thus, Funnel-shaped Mycobacterium smegmatis porin A (MspA) was introduced as an alternative to hemolysin [116, 134]. Unlike mushroom-shaped α-hemolysin, MspA has a reduced passing number of nucleotides in the stem [135]. To improve the readout of ONT nanopores, CsgG (Curli-specific gene products A-G) *Escherichia coli* outer membrane lipoprotein was also introduced [136]. Out of tens of nanopores tested and thousands of mutants, the CsgG pore had a very narrow and well-defined passage for a DNA strand and outsmarted all the pores tried by ONT [137, 138]. Later CsgG pore was engineered with reading heads that improved the signal and accuracy of the sequence readout [139, 140]. Other protein nanopores include Outer membrane protein F (OmpF), Outer membrane protein G (OmpG), Aerolysin, Nocardia farcinica peptide A/B (NfpA/NfpB), and cytolysin A (ClyA) were also been tried [112, 141] (Fig. 7, Table 2).

**Table 2:** Different biological nanopores characteristics. Adapted from [142]

Biological nanopore	Diameter (nm)	Analyte	Comment
α-HL	1.4	RNA, ssDNA, aa, polymers, peptides, proteins	Large-scale application due to its reproducible structure and easy manipulation by site-directed mutagenesis
MspA	1.2	ssDNA, dsDNA	Suitable geometry for nanopore DNA sequencing
Phi 29	3.6	ssDNA, dsDNA	Allowing for the detection of large analysts and offering more space for further modifications
ClyA	3.3	ssDNA, proteins	Suitable for the accommodation of small to medium-sized proteins within the nanopore lumen
FhuA	2.4	Enzymes, protein–DNA interaction	Examining the proteolytic activity of an enzyme at pH 3.9 and determining the kinetics of protein–DNA–aptamer interactions
Aerolysin	1–1.7	Peptides, proteins	Sensing the α-helix peptides and unfolded proteins
SP1	3	ssDNA	Analyzing of ssDNA

**Fig. 8:**
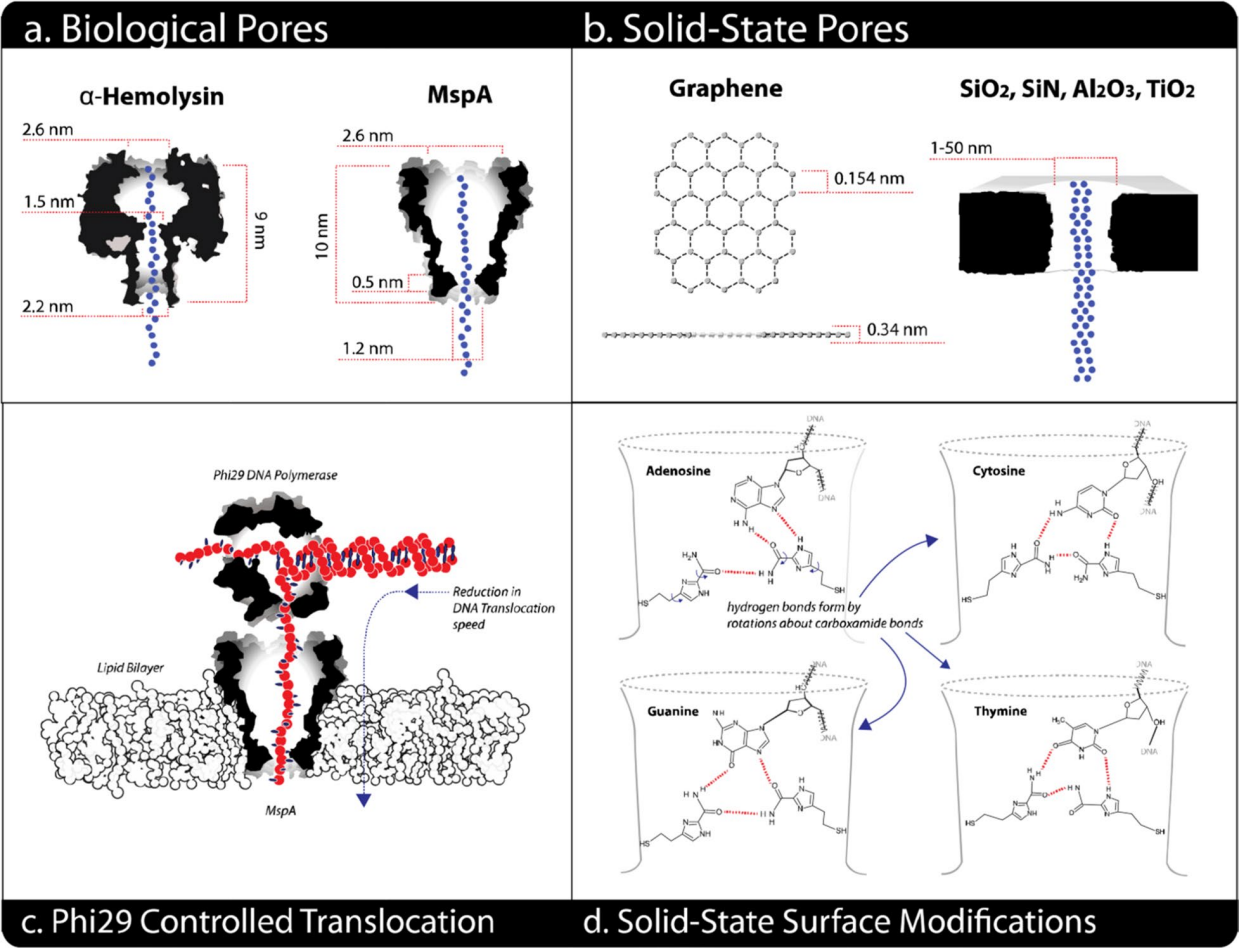
Various types and geometries of nanopores. Reprinted from [67] with permission of the publisher (CCC License ID: 600061571, 25 Nov 2021)

Diversifying the nanopore type from different building materials to get more precision, size and chemical properties have widened the application of nanopores beyond sequencing [143]. Self-assembled pore types are produced from a variety of materials including proteins, peptides, synthetic organic compounds, and DNA of various [144]. Companies like Genia technologies (acquired by Roche in $300 million aiming to combine biological nanopores with an optical detection), quanta pore, quantum Biosystems (by prof. T Kawai combining tunneling electron detector with nanopore sequencing), Base4, and Noblemen Biosciences aim to cleave single nucleotides into droplets in a water–oil emulsion and detect their presence by a chemical cascade of reactions [89, 145] (Fig. 8).

### Controlling DNA translocation through a nanopore

One of the crucial hurdles for the success of nanopores to be a reliable DNA analysis tool is the ultrafast and stochastic nature of DNA translocation, which demanded the incorporation of motor proteins to translocate DNA by base wise and other experimental modifications [107]. The origin of this problem is the velocity fluctuations due to random diffusion Brownian motion, which combine with a directed motion to create the event of a drift–diffusion process [146]. To achieve a single-nucleotide resolution, the translocation speed of the DNA is expected to be 1–100 ms/nt [107, 147]. Incorporating a biological motor or nanobead and regulating the driving voltage by adjusting pore geometry and experimental conditions are the two ways that have been tried [107, 148]. Sensing each nucleotide of a DNA strand and delivering the strand into the nanopore in a controlled manner were tried to be addressed by modifying macroscopic properties such as solvent viscosity and ion concentration or temperature [149, 150]. Molecular dynamic simulations providing a series of metal-dielectric layers have also been proposed as an additional option [151].

#### Incorporation of a biological motor or nanobead

**Fig. 9:**
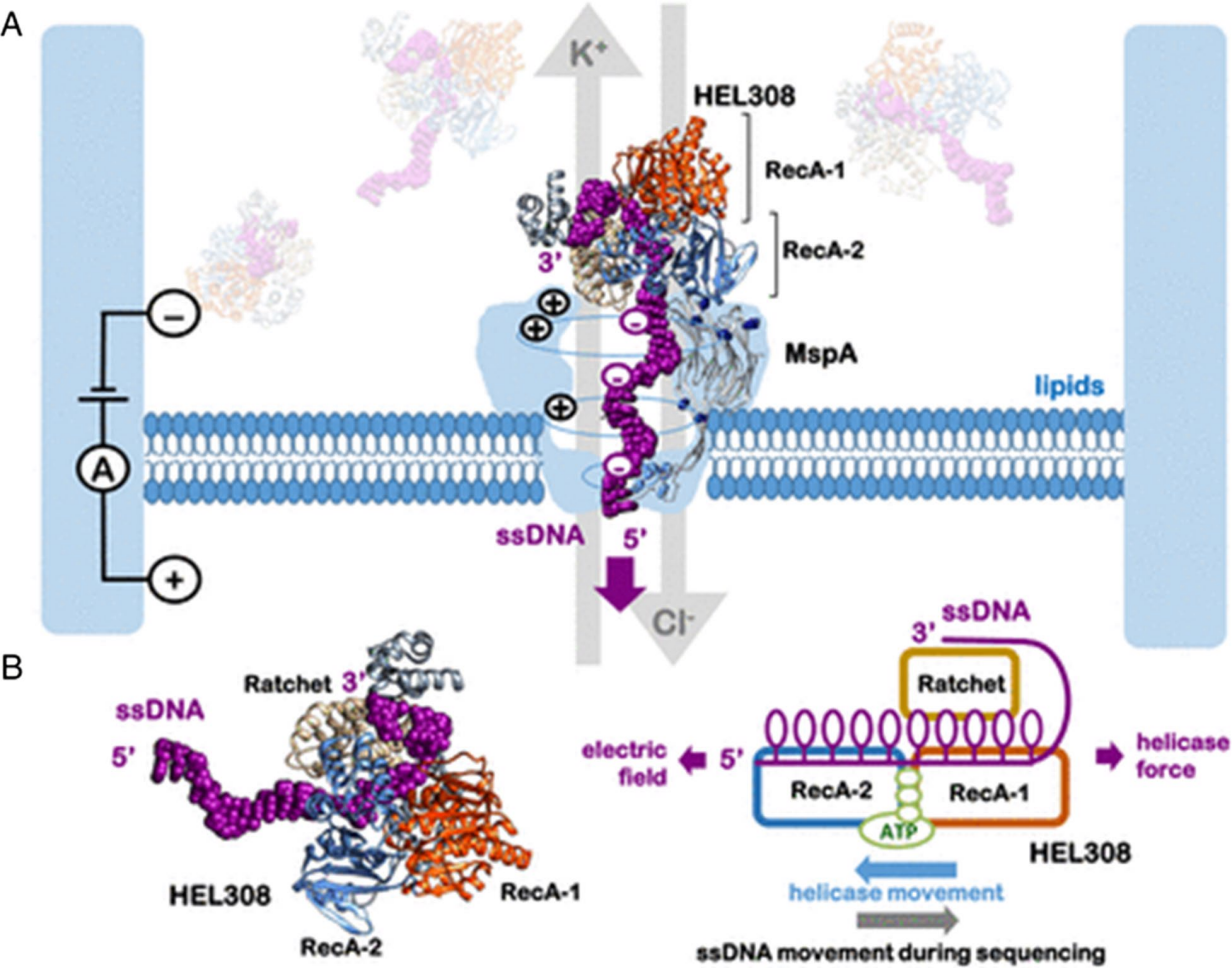
HEL308—helicase as motor protein translocating ssDNA **A** shows the mechanism and **B** Domain organization and motions of HEL308. The two (recombination protein A) RecA 1 and 2 domains compose the motor part; here, ATP binds between them and drives or rectifies the mechanochemical cycle, and the auxiliary ratchet domain makes several contacts with ssDNA and may offer determinants of the potential sequence specificity [159, 160]. Image adapted from [159]

To enhance base recognition, DNA exonuclease (from *E. coli* exonuclease I (ExoI)) and DNA polymerase enzymes were used as a motor in α-HL [152]. Weighing disadvantages like being unable to have multiple reads due to complete digestion of the strand, and the demand to have a precise feeding of nucleotides into pores, made exonuclease enzymes outdated early in motor protein studies [153, 154]. The first polymerase that is considered as A-family was the Klenow fragment (KF) of *E. Coli* DNAP I with α-HL pores [155]. However, due to stability and processivity issues, the A-family DNAP was replaced by B-family DNAP, i.e., Phi 29 [156]. The bacteriophage phi29 DNA polymerase (phi29 DNAP) has a high affinity for DNA substrates and works well with α-HL and MspA pores [157]. Unlike polymerase, helicases with the ability to bind single-stranded nucleic acids require a partial duplex where the new nucleotides are added to the 3′ end of the primer [158]. Helicase has also a better affinity, can eliminate double reading bases and skipping due to fluctuation in synthesis rate, and exhibits the proofreading trait of Phi 29-DNAP [159, 160] (Fig. 9).

An integrated nanopore platform with a nanobead structure was reported to decelerate DNA movement and the noise is reduced by a polyimide layer along with a controlled dielectric breakdown (CDB) process for nanopore fabrication [161]. The second way of controlling translocation relied on regulating the driving voltage as mentioned above, and adjusting pore geometry and experimental conditions is helpful [162–164].

#### Adjusting pore geometry

Limited pore geometries were the factors that forced research to expand into solid-state nanopores, which can give diversity in pore shape. But, they have reduced spatial resolution due to the required thickness needed for membrane stability [107, 119, 165]. Decreasing the nanopore diameter to almost the same size as that of ssDNA, i.e., 1.4 nm, decreases the translocation speed to 1.4 microsecond/base, making narrowing the nanopore one effective way to improve translocation [166]. When the pore diameter is reduced, the amplitude of current signals from DNA increases. Compared to cylindrically shaped nanopores on a continuum modeling system, conical-shaped nanopores produce greater signal amplitudes from biomolecule translocation [167].

#### Adjusting experimental conditions

The ultrafast translocation speed of single-stranded DNA (ssDNA) in solid-state nanopores is one of the predicaments, and there are various ways to decelerate the speed [161, 166, 168], one of which is controlled dielectric breakdown (CBD) with a divalent metal cation especially Ca^2+^ provides a silicon nitride nanopore with a deceleration of 100 microseconds per base [169]. Pore-dwelling time was shown to be increased by varying electrolyte cationic species and solution molarities. For solid-state pores, when the cation size decreases from K^+^ to Na^+^ to Li^+^, translocation time strongly increases both for dsDNA and ssDNA and that is due to the stronger binding capacity of smaller cations to the DNA strand [170]. Slowing down of DNA translocation velocity using a LiCl salt gradient and nanofiber mesh was implemented to maintain the DNA molecule in the sensing time of nanopores. Compared to other alkali solutions, LiCl can extend the dwell time by 20 ms (five times longer than NaCl and KCl) for which it reaches 100 ms when the concentration increases and the nanofiber mesh further retards it by 162 to 185 ms [171]. Lowering the translocation speed of ssDNA by using 15-fold increases in LiCl salt concentration brings counter-ion binding and effective lowering of the overall charge of DNA, which in turn lessens the electrophoretic driving power of the system to slow down the translocation velocity. Lowering the translocation enhanced resolution until it allows 5’mC to be distinguished from C without using methyl-specific labels is mandatory [172]. On the other side, decreasing the KCl concentration from 1 to 0.1 M resulted in a shorter time to pass through the nanopore and oppositely longer transit time was gained with a low concentration of MgCl_2_ in silicon nitride nanopore systems [173].

#### Enhancing the signal-to-noise ratio SNR

**Fig. 10:**
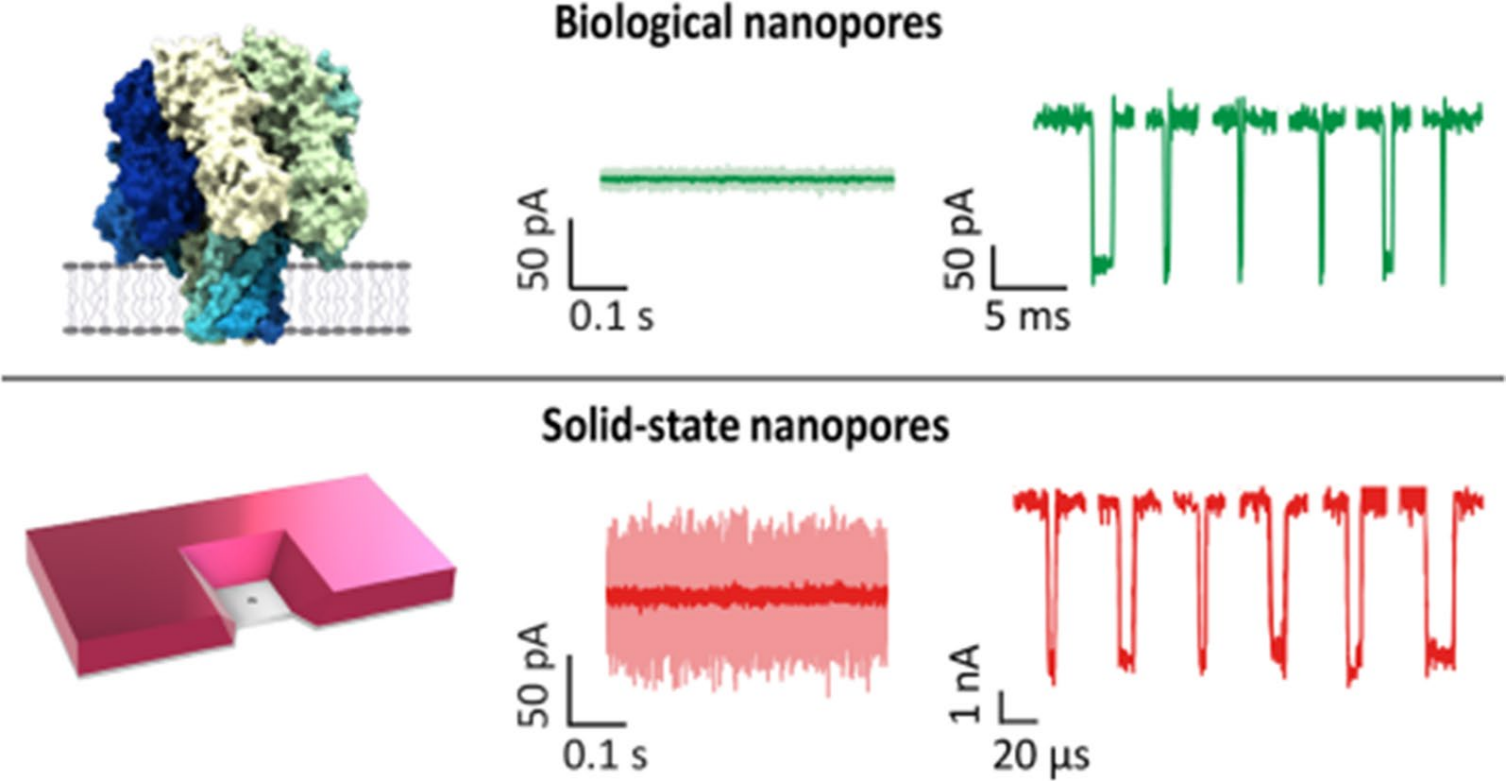
Noise in biological and solid-state nanopores. Image adapted from [174]

The major hurdle in the progression of nanopore technology is noise in the ionic current, limiting the signal-to-noise ratio (SNR). Solid-state nanopores have the highest SNR due to the large currents at which they can be operated and the relatively low noise at high frequencies. Still, the translocation speed slowdown plays a major role and MspA was shown to increase the SNR > 160 fold [174] (Fig. 10).

**Fig. 11:**
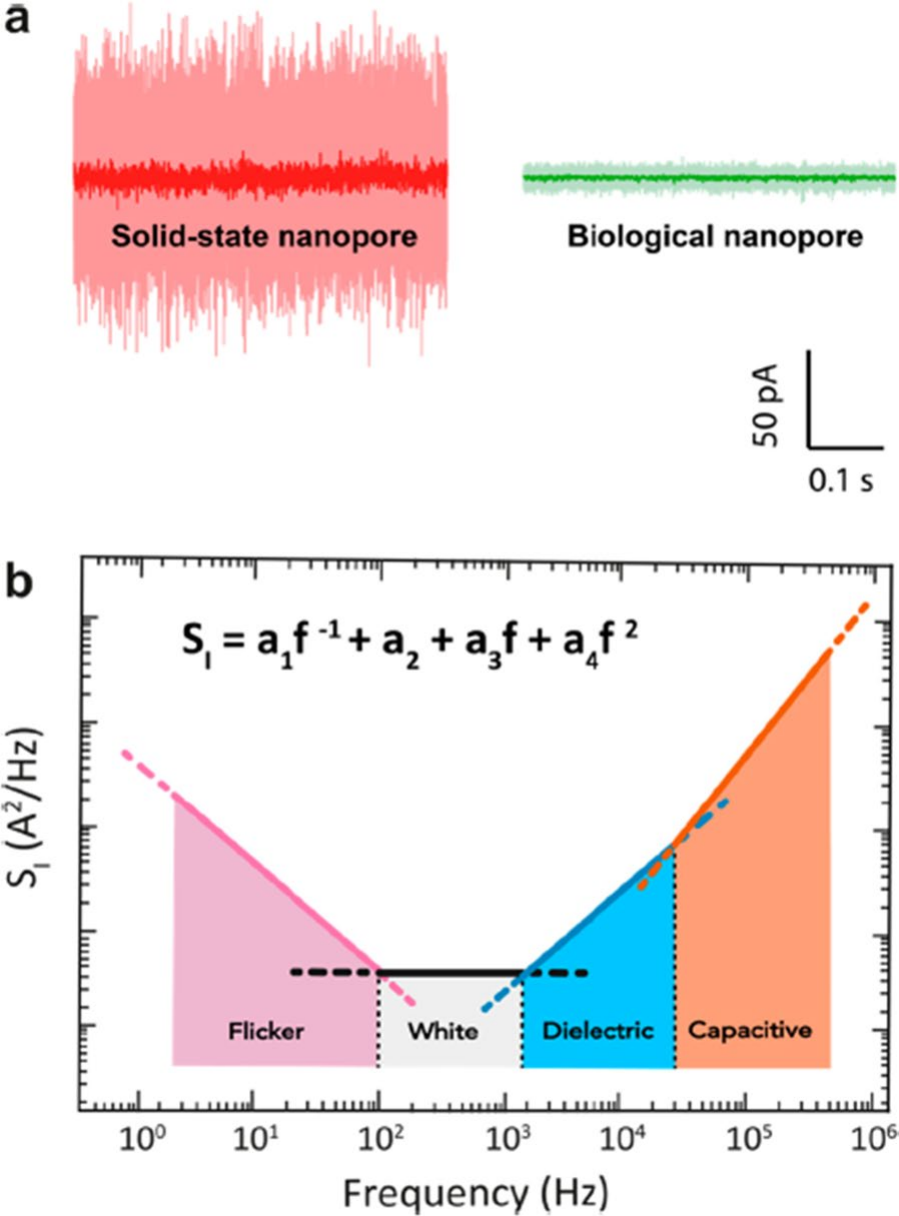
Ionic current noise in nanopores for solid-state SiN_2_ nanopores and biological α-HL (**a**) pore performed at a constantly applied bias of 100 mV in 1 M KCl buffer at pH 7 at a bandwidth of 10 kHz (**b**). Image adapted from [174]

Nanopore noise power spectral density (PSD) is composed of 1/*f* noise: white noise, dielectric noise, and amplifier noise, each dominating at different frequencies. When we see the origin of the noise, 1/f noise is due to surface and bulk effect; white noise is from thermal and shot effect; dielectric noise from dielectric membrane current leakage and amplifier noise are due to capacitance in the chip and amplifier [175] (Fig. 11).

To manipulate for SNR improvement, the diameter of the nanopore is limited by the molecule size, and the membrane thickness is constrained by material properties [176–178]. Using theoretical thickness limits of amorphous, Si membrane-based nanopore is becoming the leading material for increasing the ionic conductance and producing a high signal-to-noise ratio for sequencing applications [179, 180]. Various approaches are followed to overcome the noise limitations, for example, increasing the conformational stiffness and decreasing pore size in biological nanopores [174], surface functionalization of the SiN_*x*_ nanopores with a hydrophilic layer such as Al_2_O_3_ or SiO_2_ [124], application of high electric fields to the pore [181], choosing a pH far from the isoelectric point of the nanopore material [174] which are proved to help reduce the noise in solid-state nanopores [178]. Suppression of dielectric noise by minimizing the capacitance and dielectric loss of the chip is also another way to reduce noise [174, 175].

The other improvement area of ONT is the computational requirements for higher SNR and throughputs [182, 183]. This demands more algorithms for base calling, mapping, and variant calling [184]. Low SNR due to technological limitations of the nanopore sequencers makes it unable to read and determine the required nucleotide sequences [182].

#### Expanding the range too long reads

To sequence unambiguously spanning repetitive elements of the genome, long reads are required for increasing a significant length [187, 188]. The method of pipetting reagents as slowly as possible to minimize shearing force and preserve long DNA templates during library preparation was developed and called SNAILS (a slow nucleic acid instrument for long sequences) [187]. SNAILS implements automating the slow pipetting of library preparation reagents to increase the consistency and throughput of long-read nanopore sequencing [187, 189]. Focusing on DNA extraction and enzymatic reactions to further increase the read length, it is possible to transform from 50 to 70 kb of mechanical shearing to 90 to 100 kb reads of transposase-mediated fragmentation [190].

At the beginning of the millennium, the initial draft of the human genome was not completed and remained as such until the Oxford Nanopore sequencing technology complements the PacBio sequencing [191]. So, we see the complete set of human genomes sequenced. The remaining 8% of the genome addressed by the telomere-to-telomere (T2T) consortium included: gapless assemblies for all 22 autosomes plus chromosome X, all centromeric satellite arrays, and the short arms of the five acrocentric chromosomes [16, 192]. Long-read sequencing gets into inaccessible parts of the genome such as centromeres [101], telomeres, and acrocentric genomic regions [193]. In those regions, massive arrays of tandem repeats predominate and manifest the highest mutation rates both in germline and soma makes [194]. Identification of those techniques allowing access to the regions was a blessing for genomic analysis research and industry [101].

#### Computational advancements

Computational analysis in sequencing experiments has various tools [104, 105]. But their selection needs to be clear, and separate tools are required for individual steps. Managing and integrating the tools is also difficult. Combining tools to pipelines might help and play a role in mapping sequencing reads, calculating methylation levels, and distinguishing differently methylated positions or regions [106]. Since movement was slow to allow identification of individual nucleotides, the other challenge was creating a well-controlled ratchet of the nucleotide through the pore [87, 107, 108].

Nanopore sequencers can generate enormous amounts of data within a short period due to the development of computational systems that incorporate nanopore chemistry and base calling software [182, 184]. The software performs sequencing and reading of nucleotide fragments followed by two approaches: read mapping and de novo assembly [345]. Read mapping is an alignment of reads against the reference genome to identify variations in the sequenced genome [383]. De novo assembly is used to combine the reads for building the original sequence in the absence of a reference genome [384]. In 2014, Oxford Nanopore Technologies (ONT) launched a beta-testing program for the MinION followed by the development of novel computational approaches for base calling, data handling, read mapping, de novo assembly, and variant discovery of this new generation of data [15, 195]. These approaches improve the de novo sequencing of genomes and make possible the investigation of structural variants with unrivaled accuracy and resolution. The advancement can also reduce the higher error rate of nanopore sequencing techniques [196].

##### Nanopore chemistry software

A change in sequencing chemistry of sequencers like MinION and GridION has shown a valuable improvement in error rates. Before the production of MinION, sequencing through the biological nanopore allows 1D sequencing of a template strand up on unwinding the double strands by motor protein [182, 387]. However, early models of MinION provided 2D sequencing software that incorporates proofreading of both strands (Template and complimentary), realized due to ligation of hairpin structure to the DNA strands. The accuracy of the 2D read has been more than 5% of the 1D read (read of the template strand alone) [64]. Recently, ONT has developed 1D^2^ sequencing software that permits the sequencing of the template and complementary strands without physical ligation. Due to this change, 1D^2^ has shown an increase of 7% accuracy than 1D software [182, 385, 386].

##### Base calling software

A base calling that involves the computational process of converting the obtained raw current signals to nucleotide sequences is very important for the detection of epigenetic modifications [388]. Hence, ONT has gone through various development stages of base calling software. The base calling was obtained from fragmented current data using HMM at the early stages of development, followed by the implementation of a recurrent neural network in 2016 [389]. Raw current data have been used to collect base calling in 2017. As the accuracy demand increased, updated flip-flop and customized base calling models were practiced in 2018 and 2019, respectively [184, 390].

**Fig. 12:**
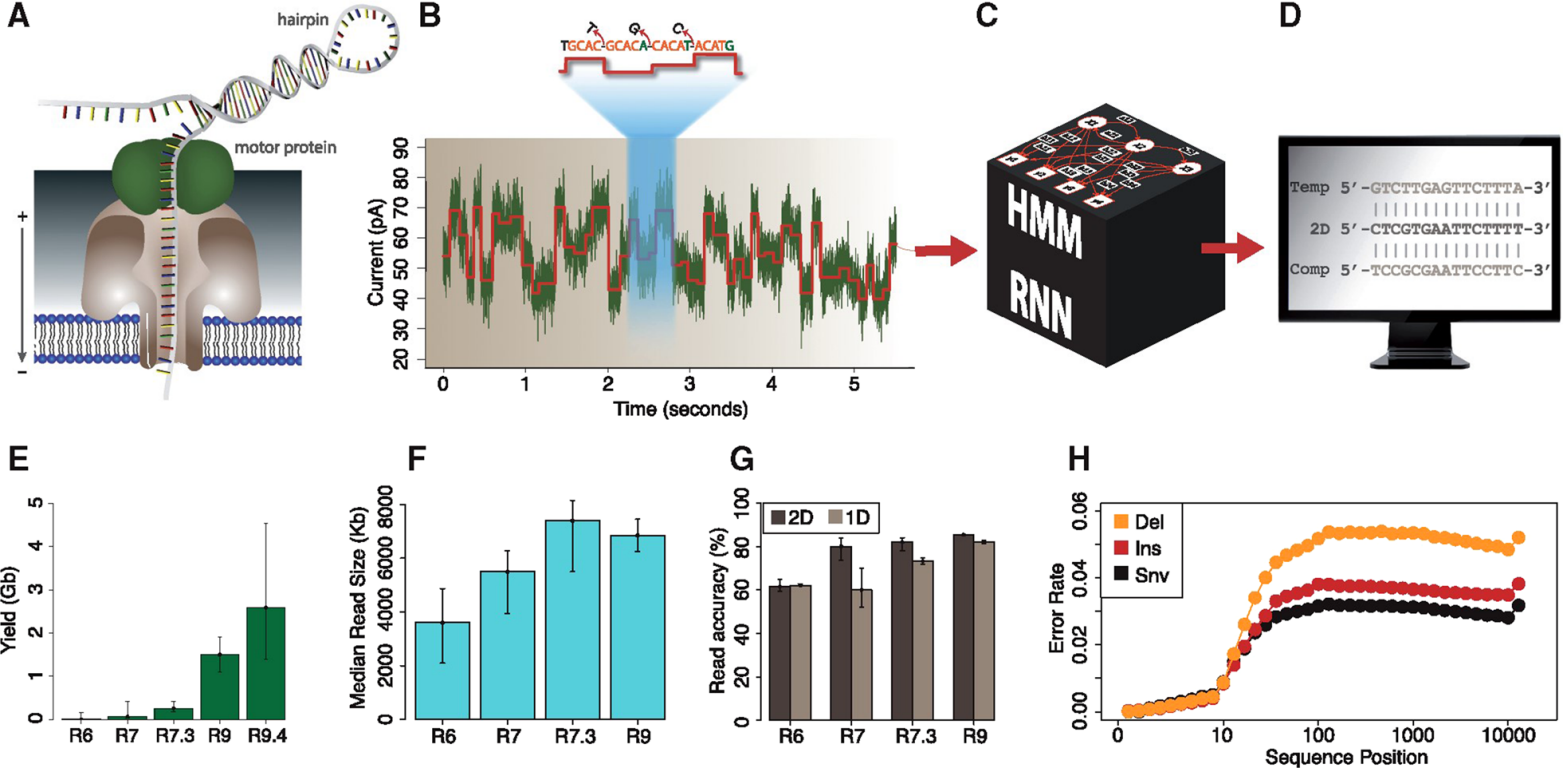
Steps for computational sequencing of DNA using a nanopore. Image reprinted from [195] with permission of the publisher (License ID: 1164222-1, 25 Nov 2021)

Real-time base calling can be simplified as the current formats like BAM/CRAM (Binary alignment map/Compressed reference alignment map) are unable to completely reach the ultra-long reads [77]. Up to five neighboring bases influence the current level of a single DNA strand that traverses through MspA [185]. Such kind of limitations inspired to use of the most dynamic programming such as the Viterbi algorithm [186]. Of course, genotyping accuracy is racing short-read sequencing instruments and it is because of insufficiency to discriminate between heterozygous and homozygous alleles. This urges a need for structural variant genotyping tools for long, single-molecule sequencing reads [77]. The computational program of MinION has identification steps to convert base calling electronic data into the required nucleotide sequences [63]. First, the motor protein found above the nanopore unwinds the dsDNA to make proper passage of the ssDNA through the nanopore (Fig. 12A). Second, the ionic current signals obtained from the nucleotide reading are segregated into mean, standard deviation, and length (Fig. 12B). Those signals have a constant sampling frequency of 5000 Hz. Third, the segregated results are then transferred to the machine learning approach box for translating into the template and adjunct signals (Fig. 12C). Finally, the sequence of signals results in a display with the computer device (Fig. 12D). The performance of each step can be evaluated through graphs based on throughput, read length, and accuracy (Fig. 12E, F, G, H) [195].

## Current challenges and opportunities of nanopore sequencing technology

The two challenges that need to be solved in nanopore sequencing are enzyme turnover and the interval in which the nanopore current is released [67, 186]. The enzyme turnover is used for the identification of successive bases in the sequence stochastic, giving an imperfect ratchet in which the interval between each advance of DNA is variable [197]. Some of the intervals may be so short, overlooked in system noise, or repetitive sequences of identical bases may not be recognized in long intervals. Improved ratcheting mechanisms for accurate nanopore sequencing might solve the issue [186].

Solid-state nanopores modification and functionalization for mimicking some of the important biological pore characteristics are advancing. However, nanopores are single-use only and require more effort to achieve reversible functionalization [198, 199]. Therefore, a hybrid biological/artificial nanopore is the most promising strategy to combine robustness and selectivity [200–202]. Nanopore technology in terms of consensus base calling accuracy is unable to compete with other sequencing platforms [203, 204]. Single-molecule sequencing (SMS) has trouble producing sufficient signals, and as a result, the error rates of the individual sequencing reads are higher than SBS sequencing data [205, 206]. Of course, nanopores enabled genome-wide and transcriptome-wide analysis on top of these base modifications in epigenomics. Additionally, as a nanopore technology being applied to protein sequencing too, for proteomics, the opportunity brings the multi-omics to a single platform, which would be nanopore sequencing, the future of sequencing for all applications including in human health and medicine [207–209].

The competition with PacBio and the biggest market shareholder Illumina is enormous. Although high-coverage sequencing is required in SMRT, detection with high accuracy is possible using low-coverage reads in nanopore sequencing [209, 210]. It has been easy for Oxford Nanopore to defeat both Illumina and PacBio on the battlefields of legal charges; it seems to continue as such due to super-packed patents held by Oxford Nanopore Company for producing, hunting, and claiming for more than a decade [211–213].

Even though many solutions emanate to the challenges as mentioned in Sect. 3, the decade-long journey of nanopore sequencing technology challenges remains still concerning for the adepts working on the technology. Daniel Branton once predicted in his “the potential and challenges of nanopore sequencing” paper in 2008, those similar challenges still exist, but great advancements have been made too [108].

### Workflow for Nanopore sequencing

**Fig. 13:**
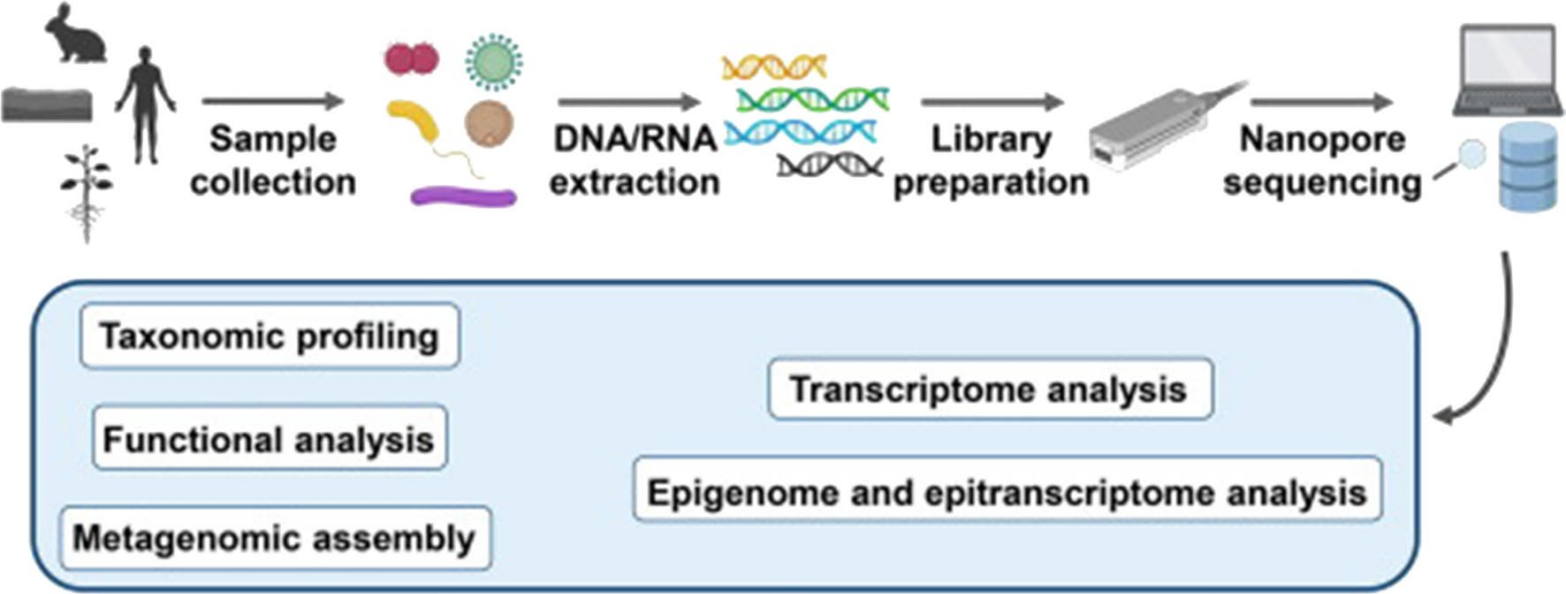
The workflow for nanopore sequencing. Image adapted from [216]

All relevant regulations for working with human subjects should be compiled before sample and library preparation for nanopore sequencing proceeds [214]. Extraction of nucleic acids followed by library preparation and base calling was subsequently performed [66]. Before sequencing and assembling large DNA fragments from short DNA oligonucleotides, a general step is increasing the nanopore sequencing throughput of small DNA amplicons [214, 215] (Fig. 13).

**Fig. 14:**
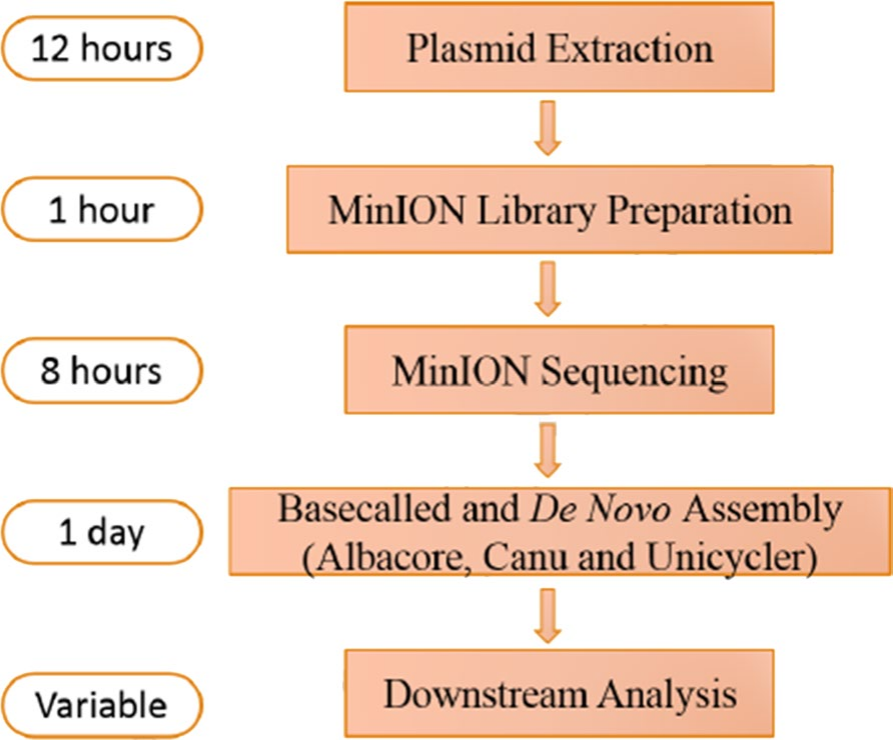
Workflow and period for MinION nanopore sequencing and assembly process. The estimation was based on a rapid barcoding sequencing kit, which could pool twelve samples in a single run. Base calling and de novo assembly are dependent on the computer’s capacity used. Image adapted from [218]

Mapping of nanopore reads is done by alignment to the reference genome with Minimap2. For reads matching known genes, the gene name is added to the corresponding SAM record using the Sicelore Add Gene Name Tag method; here, the genes are annotated with their nanopore read sequence and read qualities [217] (Fig. 14).

## Epigenetic tumor heterogeneity and sequencing technologies

### Epigenetics and tumor heterogeneity

#### Epigenetics

**Fig. 15:**
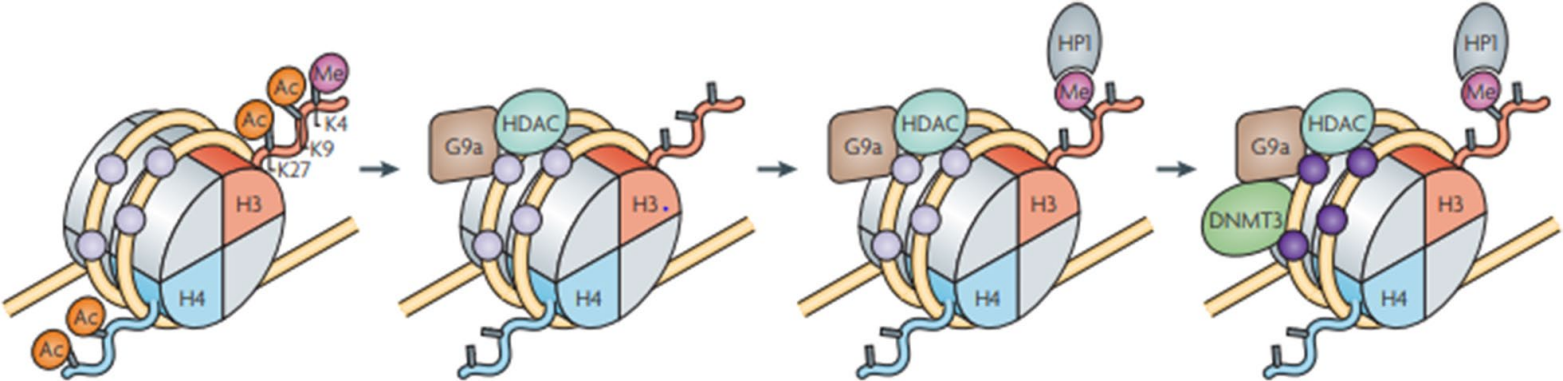
The linkage between DNA methylation and histone modification in pluripotency genes. In embryonic stem cells, pluripotency genes such as Oct 3/4 and Nanog have acetylated (unmethylated) CpG islands. These islands are combined with acetylated Histones (Ac) H3 and H4 and methylated (Me) lysine 4(K4) of Histone H3. With the initiation of differentiation histone methyltransferase (G9a) together with histone deacetylase (HDAc) enzyme binds to the complex. The binding leads to deacetylation of H3 and H4. At the same time demethylation of K4 is catalyzed by HDAc and methylation of K9 is catalyzed by G9a. This modification created a binding site for the chromodomain protein heterochromatin protein 1(HP1). Finally, G9a recruits the methylases DNMT3A and DNMT3B (dark purple circles), which will mediate the de novo methylation of the deacetylated DNA [232, 234]. The process favors epigenetic silencing and methylation while blocking heterochromatinization. Image reprinted from [235] with permission of the publisher (Request ID 600061575 25 Nov 2021)

Epigenetic components could be conceived as writers, readers, and erasers; writers add chemical groups to histones or DNA (e.g., histone acetyltransferase HATs, histone methyltransferases HMT, or DNA methyltransferases) [219]. Erasers like histone deacetylases HDACs or histone demethylases HDMTs remove the added chemical groups [220–222]. A set of reader domains that act as effector proteins by attaching to specific sequences, e.g., methyl-binding domain proteins or Bromo and extra-terminal (BETs) domain proteins, are also known [220, 223, 224]. Out of this DNA methylation which refers to the modified nucleotide 5-methylcytosine (5mC) [225] is the first epigenetic factor to be identified and the main focus here. 5mC is found within all sequences but is highly rich at sequences where cytosine is immediately followed by guanine in the 5′ to 3′ direction [226]. 5mC is considered as a CpG site, while regions with high CpG sites are known as CpG islands found over two-thirds of gene promoters and can serve as epigenetic regulatory switches that restrict gene expression when methylated [227, 228]. CpG islands at the promoter region silence genes for normal developmental requirements and during tumorigenesis [229, 230]. Unlike relatively plastic transcriptional regulation done by histone modification, gene silencing through DNA methylation is more durable and persistent [231]. As a consequence, methylation is the primary epigenetic silencing mechanism used for the repression of endogenous transposons, imprinted genes, and pluripotency-related genes in somatic cells [232, 233] (Fig. 15).

**Fig. 16:**
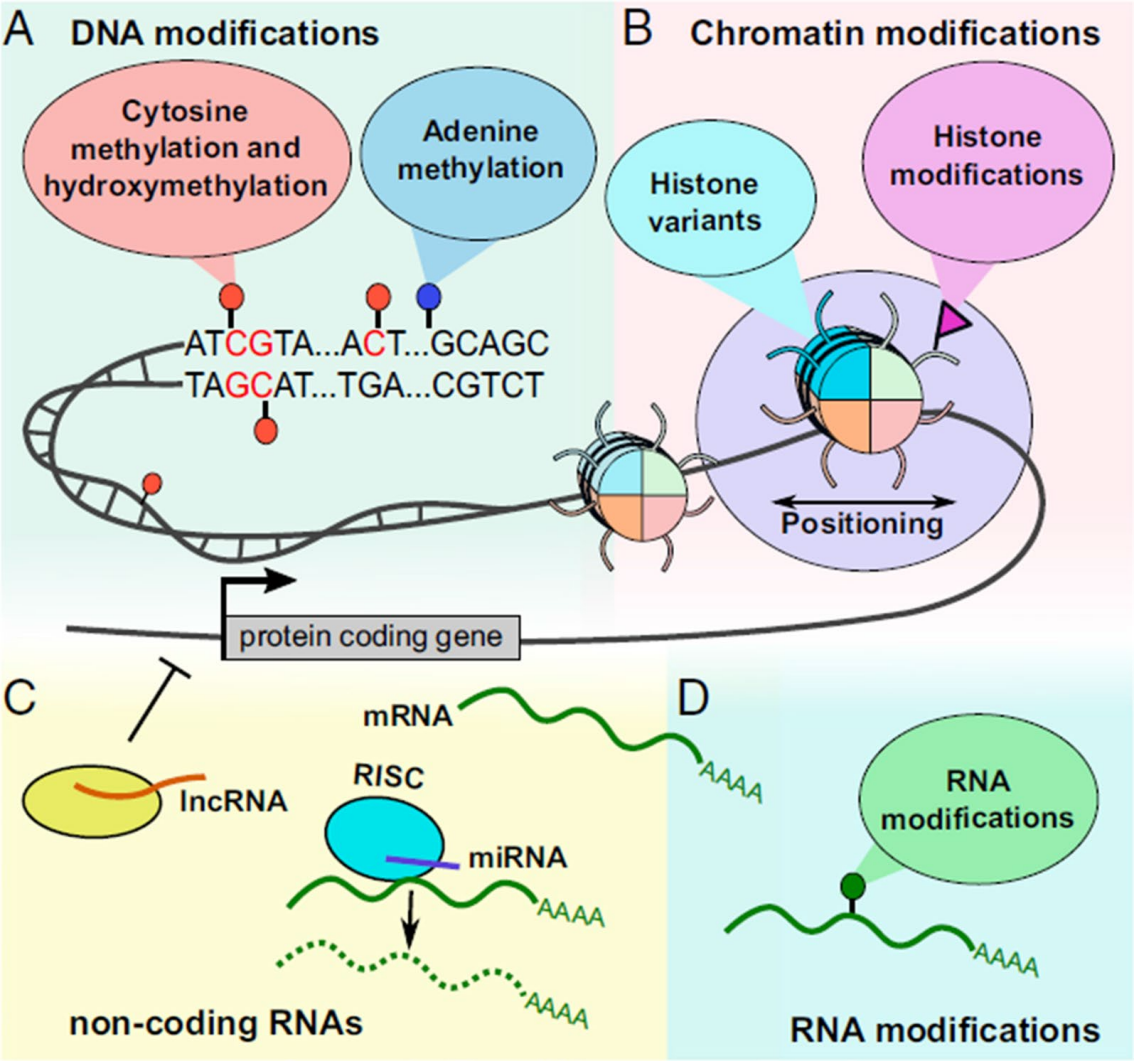
The landscape of epigenetic mechanisms. **A** Cytosine and adenine modification, cytosine by methylation, hydroxymethylation (hmC), formylation (fC), and carboxylation (caC) while adenines by methylation. **B** Histone modification and nucleosomes having different histone variants change position. **C** Non-coding RNAs play an important role in transcription regulation and are sometimes considered epigenetic mechanisms. **D** All RNA modifications can also be considered as a part of epigenetics. Image adapted from [240]

Other than methylation, there are additional dinucleotide modifications with potential regulatory roles such as 5-hydroxymethylcytosine (5hmC), 5-formylcytosine (5Fc), and 5-carboxylcytosine (5CaC) [236]. DNA methylation at the 5th position of cytosine forms 5-methyl cytosine (5mC), which is the main DNA modification occurring mostly in CpG dinucleotide sites of mammals. 5mC can be converted to 5hmC, 5Fc, and 5CaC by ten–eleven translocation families of enzymes called α-ketoglutarate-dependent dioxygenases [237, 238]. Indeed, distribution of 5hmC is possible at protein-coding gene bodies and promoters found on long non-coding RNAs, LncRNAs (Fig. 16) [239].

The regulatory function of methylation, especially in hypermethylation, lays in the recruitment of co-repressors after the promoter regions of a gene get extra methylation [241]. Such extra methylation leads to transcriptional silencing. The regulation process is directed by DNA methyltransferases (DNMT 1, 2, 3A, and 3B) and methyl-CpG-binding proteins, which identify methylcytosine residues to attract transcriptional repressor complexes like histone deacetylases (HDAC) [27, 242, 243]. Histone acetylation (HAT) and histone deacetylation (HDAC) ultimately affect gene transcription as regulators [27]. There are small RNAs that manage scaffolds that are complementary and nascent but used as an agent to guide histone and DNA methyltransferases [244]. Apart from small RNAs, chromatin-associated long non-coding RNA scaffolds play an independent but co-transcriptional silencing role that provides a system to detect and silence inappropriate transcriptional events [245]. This system also allows the registration of memory for what is carried out as self-reinforcing epigenetic loops [246] (Table 3).

**Table 3:** Chromatin modifications, readers, and their functions. Adapted from [247] with permission of the publisher (License ID 1165278-1, 01-Dec-2021)

Chromatin modifications, readers, and their function			
Chromatin modification	Nomenclature	Chromatin-Reader motif	Attributed function
*DNA modifications*			
5-methylcytosine	5mC	MBD domain	Transcription
5-hydroxymethylcytosine	5hmC	Unknown	Transcription
5-formylcytosine	5fC	Unknown	Unknown
5-carboxylocytosine	5caC	Unknown	Unknown
*Histone modifications*			
Acetylation	k-ac	Bromodomain Tandem PHD fingers	Transcription, repair, replication, and condensation
Methylation (lysine)	K-me1, K-me2, K-me3	Chromodomain, Tudor domain, MBT domain, PWWP domain, PHD fingers, WD40/β propeller	Transcription and repair
Methylation (arginine)	R-me1, R-me2s, R-me2a	Tudor domain	Transcription
Phosphorylation Ser and Thr	S-ph, T-ph	14-3-3, BRCT	Transcription, repair, and condensation
Phosphorylation (tyrosine)	Y-ph	SH2	Transcription and repair
Ubiquitylation	k-ub	UIM, IUIM	Transcription and repair
Sumoylation	k-su	SIM	Transcription and repair
ADP ribosylation	E-ar	Macro domain, PBZ domain	Transcription and repair
Deimination	R-Cit	Unknown	Transcription and decondensation
Proline isomerization	P-cis–trans	Unknown	Transcription
Crotonylation	K-cr	Unknown	Transcription
Propionylation	K-pr	Unknown	Unknown
Butyrylation	K-bu	Unknown	Unknown
Formylation	K-fo	Unknown	Unknown
Hydroxylation	Y-oh	Unknown	Unknown
O-Glc-NAcylation (Ser and The)	S-GlcNAc; T-GlcNAc	Unknown	Transcription

The role of oxidized 5-methylcytosine was controversial for a long time, but the discoveries of binding proteins as a reader to these sites started to show their roles [248, 249]. For 5hmC, a reader protein like UHRF2 (Ubiquitin-like with PHD and ring finger domains 2) was recognized [250]. But, downstream biological effects of this binding have not yet been identified [248, 251]. 5fC and 5caC exist in low amounts specifically in certain genomic locations like enhancers and promoters, and targeted studies have identified binding proteins for those modified nucleotides [252].

#### Association of epigenetic dysregulation with cancer and targeted therapeutics

The advancement of molecular sequencing technologies to characterize epigenetic aspects has made it one of the other hallmarks of cancer [253, 254]. DNA methylation profiles regulate key cellular processes such as apoptosis, lipogenesis, and downstream transcriptional effects of the MAPK-pathway [255]. Uncontrolled regulation of methylation in those gene regions results in the growth of tumor cells in colorectal cancer (CRC) [256]. Further, methylation-associated epigenetic driver genes have been identified to be involved in the early stages of tumorigenesis in CRC. CRC tumors display CpG island methylator phenotypes (CIMPs). Those phenotypes show high concordance with specific genetic changes, disease risk factors, and patient outcomes [257]. So, hypermethylation of the CpG island region leads to the silencing of tumor suppressor genes to cause the growth of tumor cells [258], while hypomethylation of the CpG island promotes transcriptional oncogenes [259]. Dysregulated epigenetic mechanisms, methylation, and histone modification are also highly associated with the occurrence of glioblastoma [260].

5hmC has specific characteristics which make it suitable for biological functions, majorly to block 5mC-seeking protein interactions with DNA [261, 262]. As a transient intermediate, it has a role during germ cell and early embryonic development to facilitate DNA demethylation [263–266]. During cell differentiation and reprogramming, TET-mediated DNA demethylation is started with the oxidation of 5mC to 5hmC [267–269]. With further oxidations, 5hmC is transformed to an intermediate 5caC and eventually completes DNA demethylation when converting to cytosine [266–270].

On gene bodies and promoters, 5-hydroxymethylcytosine (5hmC) has various roles in cancer hallmarks and differential 5hmC levels were correlated with clinical outcomes and tumor status in colorectal cancer (CRC) patients [239]. 5hmC on the other way has a role in the regulation of DNA functions that makes it one of the early cancer diagnosis and prognosis markers in the future [271, 272]. This expectation comes after the recognition of 5hmC as a transitional state intermediate that has its role to play in the demethylation process of genetic regulation [263, 273].

Generally, epigenetic aberrations of DNA methylation, histone modifications, chromatin remodeling, and micro-RNA can show cancer development and progression and are used as biomarkers for patient stratification [274, 275]. They are also used as predictive models to allow the use of cancer epigenetics in the diagnosis, prognosis, and treatment of patients [274, 276] (Table 4).

**Table 4:** Epigenetics role in tumorigenesis and progression. Adapted from [247] with permission of the publisher (License ID 1165278-1, 01-Dec-2021)

		Enzymes/readers	Mutation	Tumor
Cancer mutations affecting epigenetic regulators of DNA methylation	Methyltransferase	DNMT3A	M, F,N,S	AML, MDS, MPD
	Hydroxymethylation and derivatives	TET1	T	AML
	Hydroxymethylation and derivatives	TET2	M, N, F	AML, MPD, MDS, CMML

Epigenetics study moving deep in exploration to targeting epigenetic aberrations as a potential anticancer therapy is suitable for reversible nature of epigenetic changes [277, 278]. Several epigenetic inhibitor agents have been developed and approved for use in routine clinical practice [253, 254, 279]. The mechanism of epigenetic therapy comprises inhibitors of methylation or demethylation and acetylation or deacetylation of DNA and histone proteins [253, 280–282]. Inhibitors of epigenetic regulatory mechanisms include various analogs of adenosine, cytidine or deoxycytidine or non-nucleoside small molecule inhibitors for DNMT and hydroxamic acids such as trichostatin A (TSA) and suberoylanilide bishydroxamide (SAHA) for HDAC [27, 283]. Epidrug designs have targeted HDAC inhibitors such as SAHA and romidepsin for refractory cutaneous T cell lymphoma [284, 285], belinostat for peripheral T cell lymphoma [286, 287], or panobinostat for multiple myeloma including decitabine as DNMT inhibitor for hematological malignancies such as myelodysplastic syndromes, acute myeloid leukemia and chronic myelomonocytic leukemia [220, 288, 289].

Numerous epigenetic biomarkers with cancer detection, diagnosis, and/or prognosis capability have been identified [290, 291]. However, their clinical availability is low. Lack of independent validation and variable experimental designs in multicenter groups hindered the advance of translational studies to convert the markers to clinically useful tools [292]. The lack of validation also hinders the availability of easy and affordable testing for cancer [290].

#### Tumor epigenetic heterogeneity

Heterogeneity of tumors could occur among patients, in the same patient of multiple tumors with the same origin or within a tumor subpopulation, which is called inter-patient heterogeneity, intra-patient heterogeneity, or intra-tumor heterogeneity [23, 293]. As a survival mechanism in various environmental conditions, DNA modification among individual cells is an important epigenetic factor that can regulate phenotypic heterogeneity [294, 295]. Substantial heterogeneity in expression is found even among morphologically indistinguishable cells, which play an important functional role in tissue biology and disease states such as cancer [233].

In human cancer, epigenetic aberrant changes occur more frequently than gene mutations [23, 296, 297]. However, the majority of cancer research focuses on the genetic bases, particularly mutational activation of oncogenes or inactivation of tumor suppressor genes (TSG) [23]. In several lineages of tumor cell differentiation programs, epigenetic mechanisms are integral parts and have a potential molecular link between cancer, stem cell biology, and drug resistance [24].

**Fig. 17:**
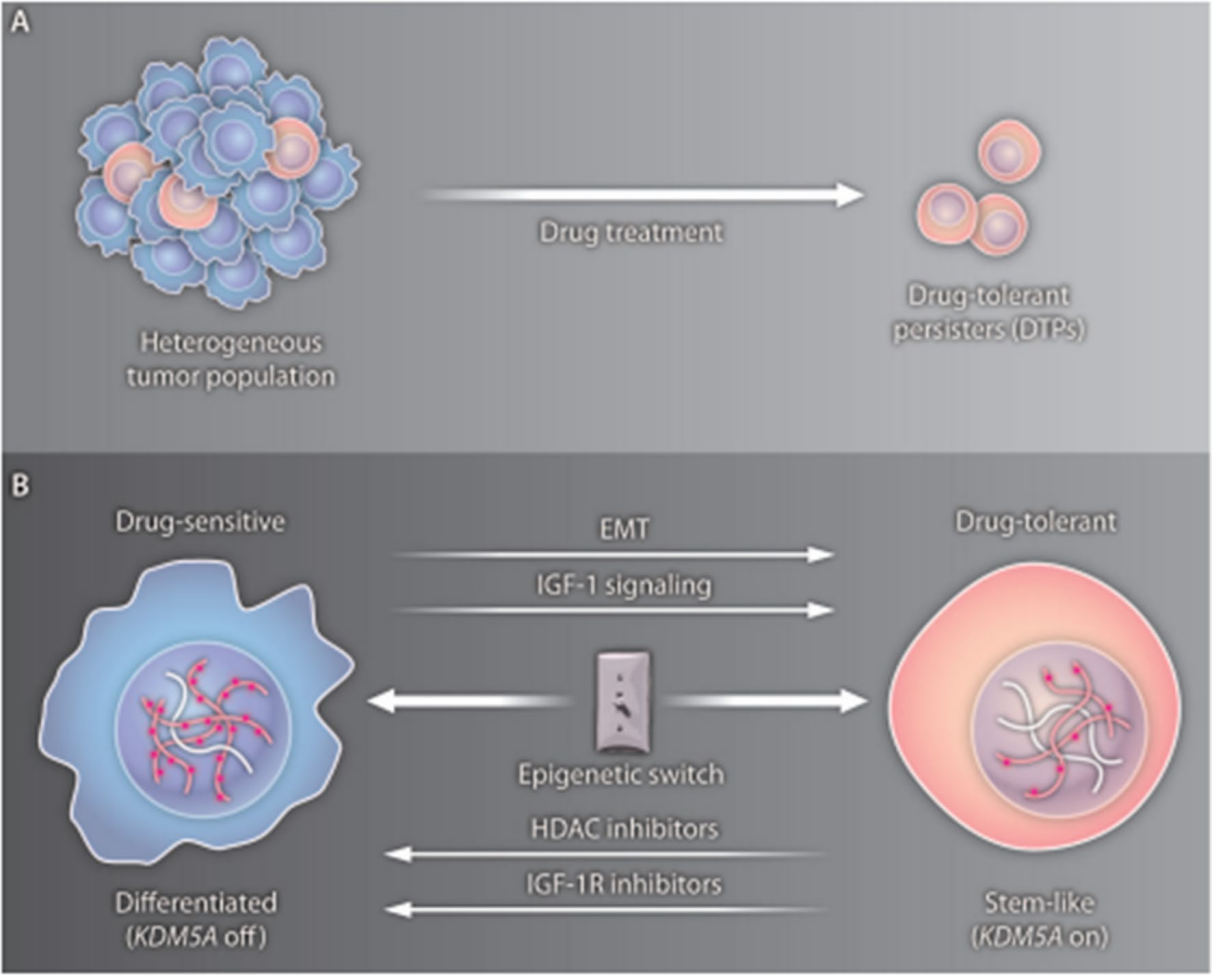
Tumor cell heterogeneity results in a drug-tolerant phenotype of the tumor. **A** Selection of a subset of DTPs after treatment. **B** Epigenetic changes mediate the transition between drug-sensitive to drug-tolerant states. Image adapted from [24]

**Fig. 18:**
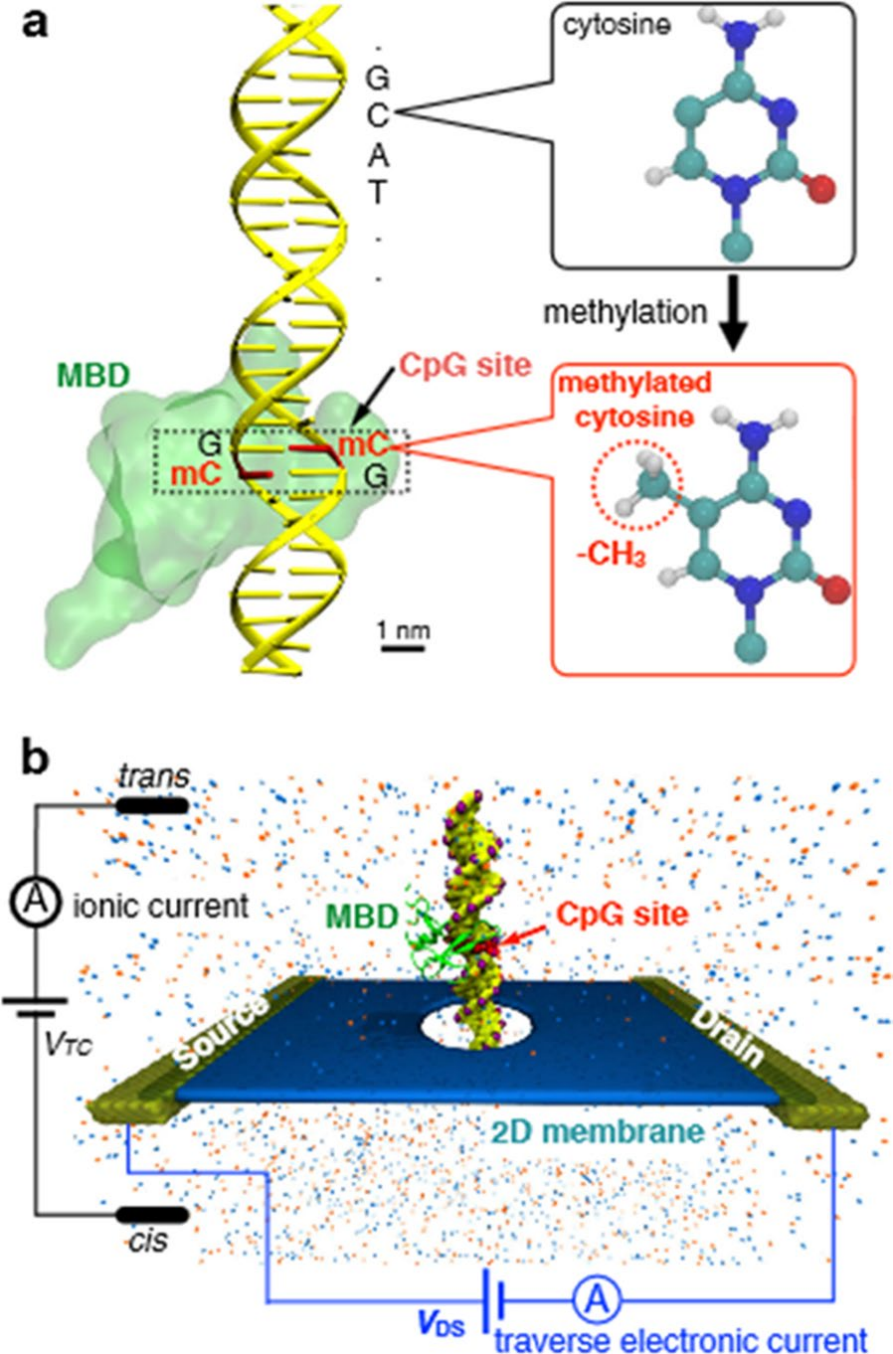
Identification of methylated cytosine residues using solid nanopore synthesized from 2D graphene or MoS_2_. Image adapted from [351]_._ **a** Discrimination of C and mC structures with the help of MBD1 protein. The methylation occurs in the fifth carbon position of the cytosine ring structure, and most of the mC nucleotides are found in the CpG island region of the gene. **b** Diagrammatic detection model of the mC during nanopore sequencing of DNA. The identification is based on utilizing ionic current differences obtained from the application of the required voltage

The level of methylation heterogeneity was found to be correlated with times of relapse-free and overall survival in 79 intra-tumor colorectal tumors [298, 299]. Abundant evidence supports that tumors are frequently composed of heterogeneous cell types to which drug resistance appears to be linked [300, 301] and the role of epigenetic mechanisms for mediating drug resistance in subpopulations of cancer cells has compelling evidence [24, 302] (Figs. 17, 18).

### Mapping epigenetic heterogeneity in tumor

#### Roles of epigenetic sequencing in tumor heterogeneity

When we looked at the physiological functions of the TET proteins and their mechanisms of regulation of DNA methylation and transcription, out of the three TET genes TET1 and TET2, expression levels were shown to be low in hepatocellular carcinoma (HCC) tissues [303, 304]. Studies have also revealed that global genomic 5hmC levels are down-regulated in HCC tissues and cell lines [305, 306]. For designing early detection and therapeutic strategies, 5hmC signatures found in HCC tissues and in circulating cell-free DNA are important [305]. Functions of 5-hydroxymethylcytosine (5hmC) in gene regulation and cancer pathogenesis were studied by using sequenced cell-free 5hmC obtained from 49 patients with seven different cancer types. The finding showed that distinct features are available to predict cancer types and stages with high accuracy. The study also suggested that cell-free 5hmC signatures may potentially be used to track tumor stages in some cancer types [307].

Cancer-associated 5hmC signatures were identified in cfDNA [308, 309]. The signatures are characteristics for specific cancer types which are highly predictive of colorectal, gastric, lung, and pancreatic cancers [307, 308]. This marker has also great potential for diagnosis and prognosis of cancer from an analysis of blood samples [308, 310]. So, excelling on conventional biomarkers comparable to 5hmC is further required.

#### Conventional sequencing methods in epigenetics

DNA methylation can be assessed by: digestion of DNA with chemical conversion (bisulfite reactions), methyl-sensitive restriction enzymes, and affinity enrichment of methylated DNA fragments [311, 312]. A strategy that could distinguish 5-hydroxymethylcytosine, 5-formylcytosine, and 5-carboxylcytosine from 5-methylcytosine is important, and many strategies have been developed with their advantages and limitations [236]. Methylation sequencing and/or microarray-based profiling strategies work with NGS techniques [313]. All the epigenetic sequencing methods to map the 5mC need to work with next-generation sequencing that gives the chance to long-read sequencing both for DNA and RNA and they can directly read out the modifications at once [314].

Bisulfite sequencing (BS-Seq) is based on the reactivity difference between methylated cytosine and unmethylated cytosine brought by bisulfite treatment that deaminates unmethylated cytosine to uracil (U), while the methylated one preserves itself [315], so that, during PCR amplification, methylated cytosine remains cytosine, while unmethylated cytosine would be read out as T [314].

Though the base-resolution bisulfite method is the one taken as a gold standard, so far, it had flaws because of the harsh chemical treatment nature, degrades the majority of the DNA, and limits the library of generated epigenetic sequencing [316]. Bisulfite sequencing has many integral faults starting from missing to distinguishing between 5mC and 5hmC [317]. Bisulfite sequencing also provides combined signals such as reduction of sequence complexity leading to low mapping rates, uneven genome coverage, and inherent biases [314, 318]. Those drawbacks occur because 95% of the total cytosine in the mammalian genome is converted to thymine [314]. The most serious problem inherent in base-resolution sequencing and awaiting a possible solution to ameliorate is the degradation of the majority of the DNA during bisulfite treatment and the low conversion efficiency. The bisulfite conversion is also blind to distinguish between 5mC and 5hmc [319].

Alternative to bisulfite techniques, there have been bisulfite-free and base-level resolution sequencing methods like TET-assisted pyridine borane sequencing (TAPS) and are developed for both 5mC and 5hmC [316, 320]. TAPS combines TET oxidation of 5mC and 5hmC to 5-carboxylcytosine (5caC) with pyridine borane reduction of 5caC to dihydrouracil (DHU) [321]. The C-to-T transition completes when PCR converts DHU to thymine and TAPS detects modifications directly with high sensitivity and specificity, without affecting unmodified cytosine [322]. The method preserves up to 10 kilobases long that enable cheaper methylome analysis [316].

Another method based on oxidative bisulfite sequencing (oxBS-Seq) applies the oxidation capability of potassium perruthenate (KRuO_4_) to produce 5fC and through bisulfite treatment converts into U and the conversion rate is 94.5% [314]. Finally, the 5hmC level and position can be obtained by subtracting the oxBS-Seq from the BS-Seq [323–325]. Potassium perruthenate is more damaging than potassium ruthenate, and the latter is more helpful for nanoscale genomic mapping in limited biological and clinical samples [320]. This method is claimed to be able to detect cell-free DNA (cfDNA) of healthy donors and cancer patients, showing base-resolution hydroxymethylomes in the human cfDNA for the first time [314, 326].

Data analysis of methylation needs an efficient tool with bisulfite sequencing datasets, and the recently developed tool BSPAT (bisulfite pattern analysis) has removed multiple/pairwise sequence alignment methods for fast alignment of sequence reads. To make DNA methylation mechanisms and regulation explored, BSPAT summarizes and visualizes DNA methylation co-occurrence patterns [327].

Improvement of the cost along for accessibility and genome coverage of approaches is important especially for those of bisulfite-free methods with base-pair resolution (which are now single-molecule and single-cell analysis) [328]. The methylome’s large portion could be addressed by microarrays and next-generation sequencing technologies at genome-wide levels to generate base-resolution maps of 5mC and its oxidation derivatives in genomic samples [329, 330]. For this purpose, quantitative approaches have been established under bisulfite-based methods like classical bisulfite sequencing, pyro sequencing, etc. [331–333].

Before PCR amplification, CpG methylation at the single-base resolution can be determined by methylation-sensitive restriction endonucleases [332]. Affinity-based methods also enrich the methylated areas. But it is difficult to reach the exact site to directly determine. Moreover, the bisulfite method requires DNA denaturation and causes DNA degradation that decreases its efficiency [334, 335]. There are also PCR-caused mapping inefficiencies of bisulfite-treated DNA and bisulfite conversion rates to be considered [311].

The complexity of library preparation and incomplete chemical conversion biases increased due to the bisulfite used to convert unmethylated cytosines to uracil [25, 336]. Illumina-based sequencing fails short of short-read lengths that hinder allele-specific methylation. On the other hand, PacBio long-read sequencing lacks high sequence coverage, limiting it from sequencing the methylated nucleotides. However, Oxford Nanopore sequencing is becoming the most advanced to fit into the situation [25].

## Nanopore sequencing for epigenetic tumor heterogeneity

### Nanopore sequencing advancing epigenetic mapping

**Fig. 19:**
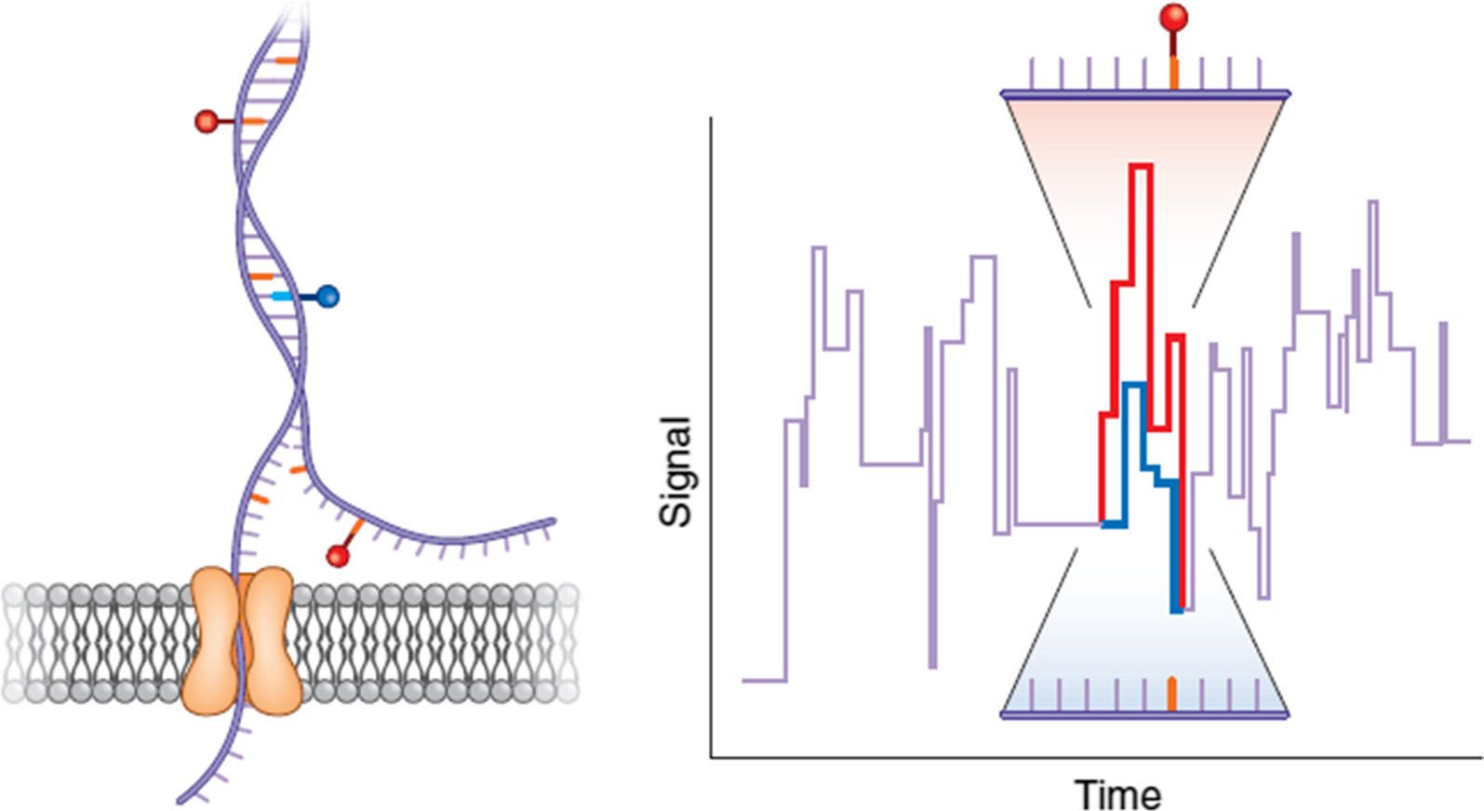
Direct reading of DNA methylation by nanopore sequencing. The ionic current is changed as single-stranded DNA passes through the pore; having a methyl group and small changes due to methylation are interpreted by a new set of algorithms. Image reprinted from [25] with permission of the publisher (Request ID: 600061678, 27 Nov 2021)

Methylation of DNA is one of the commonest epigenetic modifications that can be used in epigenetic mapping [337, 338]. Methylation also plays a vital role in mammalian gene cell expression [339, 340]. These roles include cell development, aging, and regulation of tumor suppressor genes [341–343]. However, most DNA sequencing technologies are unable to differentiate methylated and unmethylated nucleotides in a DNA strand [25, 344]. However, the discovery of the Oxford Nanopore MinION sequencer allows the sequence of methylated regulatory marks without special sample preparation, and with long-read single-molecule nature [345]. This feature makes MinION easier to study allele-specific methylation in heterogeneous cancer samples [25, 54, 346]. Limitations such as multiple nucleotides signal due to at a time entry of 5 nucleotides into the pore and current overlapping of methylated and unmethylated bases are identified [186]. Those drawbacks are resolved upon designing base-caller computational hidden Markov model (HMM) software [64, 347]. Based on the visibility of different current distributions, the software allows distinguishing three modified cytosine (C, 5mC, and 5hmC) and two modified adenine variants (A and 6-mA) [348–350]. Despite the incorporation of HMM, clear detection of DNA methylation by solid nanopore sensors constructed from two-dimensional (2D) graphene or molybdenum disulfide has also widened the validity of the process [351]. Furthermore, to detect the mC nucleotide upon passing through the nanopore, labeling of DNA methylation site by an adaptor of methyl-CpG-binding domain proteins (MBD1) is also mandatory (Fig. 19).

For studying CpG methylation and chromatin accessibility on long fragments of DNA, nanopore sequencing allows detecting sequencing difficult regions for characterization of genomic elements such as repetitive elements [352, 353]. Looking for the CCN1 gene (a poor prognosis correlated gene in colorectal cancer), methylation heterogeneity was observed in three enhancer regions with the highest activity in Enhancer 3 which is responsible for CCN1 up-regulation. The only way to decipher this is using the long-read nanopore technology [346]. By using nanopore sequencing data, the most complete human methylome is produced through long-read chromatin accessibility measurements (nanoNOMe) paired with CUT and RUN data [354, 355]. The hypomethylated region is extremely inaccessible and paired to CENP-A/B binding [354]. However, long reads interrogated allele-specific long-range epigenetic patterns in complex macro-satellite arrays existent in X chromosome inactivation can be deciphered. This single-molecule measurement clustered read based on the methylation status of epigenetically heterogeneous and homogenous provides a framework to investigate the most ambiguous regions of the human genome [354].

Augmenting the DNA bisulfite method with high-throughput sequencing technologies has widened the range to genome-wide DNA methylation than limited to CpG sites and CpG islands [356, 357]. Genome-wide DNA methylation studies show differential methylation at the genomic sites like promoters, CGIs, and respective elements [358]. Those differential methylations are sources of various clonal cell populations that create heterogeneity [359, 360]. The easiest method to identify modifications has a positive impact on epigenetics and excellent reproducibility and correlation with bisulfite sequencing. Suggestions are saying that nanopore sequencing could become the gold standard for detecting methylation patterns. As the short-read bisulfite sequencing demands differential methylation assessment, statistical methods which we lack now in long-read sequencing extend even to allow nanopore sequencing modifications in haplotypes [77, 361].

MethyQA software package solves the glitch that occurs when the unmethylated cytosine is converted into U and T while using the bisulfite conversion technique [360, 362]. Alleviated by this software, NGS technologies can output the methylation sequencing data having quality issues like: low per-base sequencing at the 3′ end, PCR amplification bias, and low bisulfite conversion rates [362, 363].

5hmC detection limitation deterred the assessing of 5hmC physiological functions and its role in demethylation pathways [364]. The limitation also affects the deep identification role performed by 5hmC: location, regulation of transcription, replication, and epigenetic reprogramming [365]. So, such determination of 5hmC functions demands the development of single-molecule DNA sequencing technologies for which nanopore sequencing best fits [365, 366].

#### Accuracy measurements for the detection of epigenetic modifications through nanopore sequencing

**Fig. 20:**
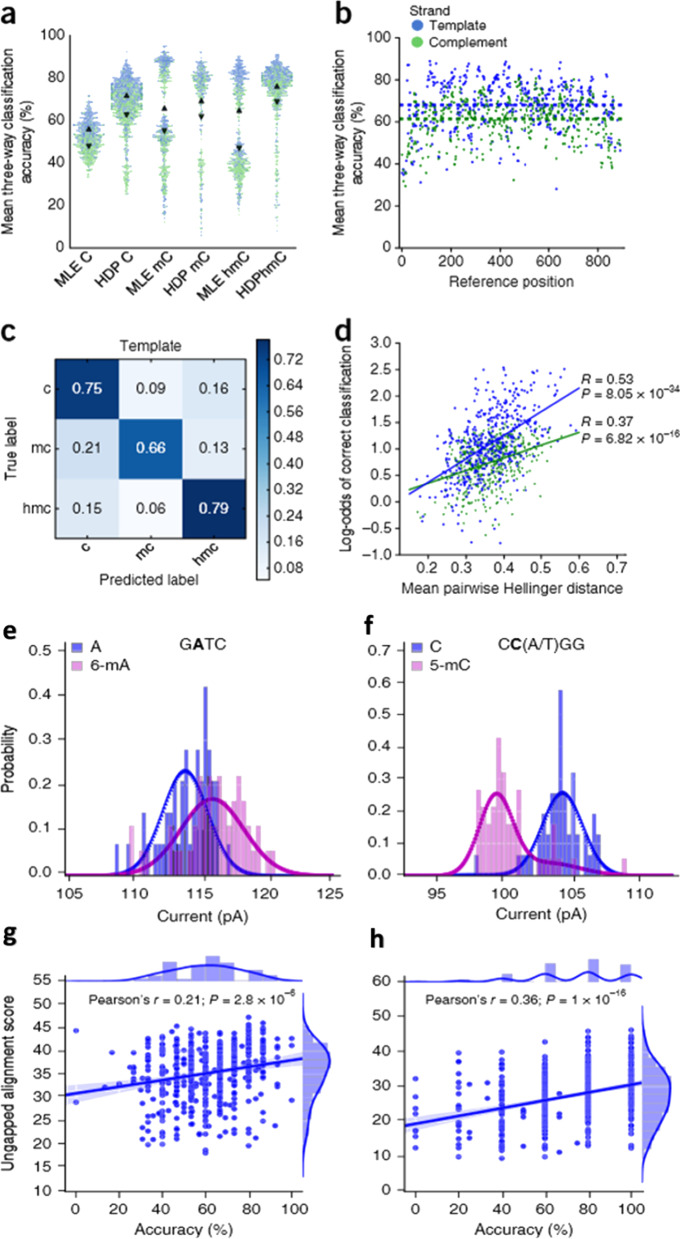
**a–d** Accuracy result of the MinION detection of cytosine methylation variants found in the synthetic oligonucleotides. Outputs from the classification of 6,966 C, 294 5mC, and 467 5hmC strands were sequenced in similar MinION flow cells. **a** Pre-read accuracy distribution results expressed by comparing normal distributions as Maximum-likelihood estimates (MLE) and HDP model distributions. Distributions are shown by triangles. **b** Across all cite three-way classification (C, mC, and hmC) of the template and co-template reads. **c** Confusion matrix showing the performance of HMM-HDP three-way cytosine classification on template reads of synthetic oligonucleotides. **d** Correlation between the log-odds of correct classification and the mean pairwise Hellinger distance between the methylation statuses of the 6-mer distributions overlapping a cytosine. **e**–**h** Variation between the ionic current distribution and effect of reading quality for left (6 mA in GATC) and right (5mC in CC(A/T) GG) motifs. The ionic current distribution between A and 6-mA (**e**) and C and 5mC (**f**) has shown a difference. Ionic current levels from 100 alignments are shown as a histogram **g** for A and 6-mA and **h** for C and mC. Learned probability densities of HDP are shown as curves. Image is Reprinted from [348] with permission of the publisher (Request ID 600061677, 27 Nov 2021)

Out of the discussed methods above, the Oxford MinION nanopore sequencing model with HMM (hidden Markov model) is reported to have the capacity to differentiate among all the modified bases of Cytosine [63, 347]. With better improvements of HMM, HMM-HDP (hidden Markov model with hierarchical Dirichlet process) model has been developed, incorporating accuracy measurements of the modified bases detected by MinION sequencing (Fig. 20a–d) [64, 348, 367]. The model discriminates among all five C^5^ cytosine variants based on ionic current measurements from low throughput nanopore sensors [368]. In HMM-HDP, the base modifications are detected as changes in the ONT-MinION’s ionic current signal. MinION frequently records ionic currents to divide them into segments called events. The design models each event as a nucleotide striking of length called K-mer [369]. Each K-mer has an alliance with a distribution of ionic currents in Picomas (P^A^). The individual C, mC, and hmC bases are classified from the synthetic nucleotide regions to measure the accuracy of detections through a change of ionic current signal. After detection of changes in the model, the distribution of the ionic current signal has to be measured to determine segregational strength (Fig. 20e–h). The model also incorporates mapping of 5mC from CC(A/T) GG motifs and 6 mA from G**A**TC motifs using *E. coli* genomic DNA [367].

### Single-cell tumor epigenetic mapping using nanopore sequencer

The field of single-cell epigenomics is in its infancy. But, due to the increasingly recognized importance of intercellular heterogeneity in tumors with the rapid pace of technological development, it is expected to show enormous progress over the next few years [370]. Single-cell epigenomics incorporates epigenetic profiling with the isolation of single-cell, barcoding it, and high-throughput sequencing of the isolated cell genome [371]. Since epigenetically modified genes are shown in most cancer cells, it is essential to use simple and lower-cost methods to identify these modifications [372]. Nanopore sequencing with recently upgraded technologies has been the easier and preferable method to detect the epigenetic modifications that occur in a specific cancer type of various organs [373].

Deletions, amplifications, inversions, and translocations of nucleotides in a DNA sequence are the four DNA replication-related causes of gene mutations. Nanopore sequencing can be used to detect the heterogeneity of tumors as a result of these changes, which led to the anticipated alterations during epigenetic modifications [391]. Additionally, nanopore sequencing is highlighted as one of the primary areas of focus for the next-generation approaches to understand the heterogeneity of cancer [392].

Beyond previously accolade genetic alterations, tumor heterogeneity derived by epigenetic reprogramming causes drug-resistant subpopulations of tumor cells [374]. It shows the need for single-cell epigenetic technology capacity to truck drug-induced tumor evolution for the timely intercession of the treatment [293]. In hepatocellular carcinoma, identification of the modification status of tumor suppressor genes using nanopore sequencing showed around 10 potential tumor suppressor gene candidates and the glucokinase gene, more validated to involve in HCC development [375]. Nanopore sequencing allows whole-genome sequencing with the possible identification of epigenetic modifications in lung cancer cell line LC2/ad gene [376]. It also allows the detection of epigenetically modified genes in various cancer types (Table 5).

**Table 5:** Nanopore sequencing for epigenetic modification study of various cancer types

Type of nanopore sequencing	Type of cancer in which epigenetic modifications are identified	References
ONT and PacBio	Breast cancer	[377, 378]
ONT	Leukemia	[379, 380]
ONT	Brain tumors	[75, 393]
Linked Read	Gastric cancer	[376]
NGS	Cervical cancer	[394]

## Main results in the epigenetics-cancer field that nanopore technology allowed

Nanopore sequencing (NGS) is still in its infancy as a tool for cancer research, and applications in molecular cancer research are particularly lacking. Of course, NGS technologies are more suited for use in the investigation of fields like plant science and microbiology. However, employing cell lines as a study medium is gradually being applied to human samples [395–397]. Even if the sequencing overcame several obstacles, there are still opportunities for improvement and benchmarking computer techniques for detecting whole-genome DNA alterations [398]. It suggested that there was a pressing need for the benchmark to be able to predict CpG methylation in multiple genomic contexts, particularly those including genes involved in tumor heterogeneity and tumor suppression.

The epigenome pattern on copies of DNA segments has been employed as a harbinger endeavor, and nanopore sequencing is still being used alongside the old standard methods midway. These patterns are determined by nanopore sequencing and allow the assignment of reads of haplotypes to enable chromosome-level allele-specific profiles of CpG methylation and chromatin accessibility on four human cell lines (GM12878, MCF-10°, MCF-7, and MDA-MB-231), which are determinants of nucleosome positioning and DNA accessibility. Then, the application of nanopore sequencing was expanded to find heterogeneity in breast cancer model tumors [346]. Due to its capacity to recognize and sequence nucleotides even when they have little alterations, nanopore sequencing is evolving into a standard to rule the sequencing market [399]. It is hoped that future methylation-mapping-complete software, like NanoMetPhase, would offer a second deep signal for the detection of 5mC and 6 mA. The software employs 2 × coverage to find any DNA base methylation states that are reliable markers for the more accurate detection of tumor heterogeneity [400]. This upbringing will support the parallel implementation of nanopore-based computational and experimental application methods.

Oxford Nanopore can be used for whole-genome sequencing to identify insertions, deletions, inversions, and intrachromosomal translocations in liver cancer, which could then be used for epigenome analysis as the instrument allows for parallel genome and epigenome sequencing to determine the complex heterogeneity and variation of tumor cells [401]. The magic of this pocket-size nanopore sequencing device was tested by sequencing simultaneously on the same day the genome and epigenome of the low-pass whole genome to generate diagnostic copy number (CN) and methylation profiles from the same sequencing run. That is the beginning of the explosion of using nanopores for important molecular classifications in cancer for better diagnosis, prognosis, and treatment decisions in clinics [75]. Another study discovered that nanopore Cas9-targeted sequencing (nCATS) is more effective at detecting isocitrate dehydrogenase 1 and 2 (IDH1, IDH2) and O6-Methylguanine-DNA methyltransferase (MGMT) mutations and methylation status in diffuse glioma in 36 h [402]. The combination of Cas9 mutation and library creation for sequencing appears to be the most effective coupling currently available, and it could aid in identifying single-nucleotide variants (SNVs), structural variations (SVs), and CpG methylations [403]. In order to enable long-range amplification and nanopore sequencing, the BRCA1 breast cancer gene's body and flanking regions are isolated from peripheral blood cells using the Cas9-assisted targeting of chromosomal segments (CATCH) method. It is reasonable to assume that this technology will eventually be available in medical offices and patients' pockets [404]. It is crucial to sequence the epigenome of tumor-specific LINE-1 insertions and their retrotransposon signatures because CpG methylation controls the transposable elements (Tes) involved in the evolution of tumor growth. [405]

Nanopore whole-genome sequencing for intraoperative neuropathological classification has been found to improve practical intraoperative diagnostic accuracy impacting surgical decisions [406], so that with the previous data accumulated for epigenomic tumor signatures in whole-genome analysis done using the chemical methods and Illumina are now the background to bounce up along with nanopore sequencing soon.

For the high-level identification of epigenetic heterogeneity in cancer, nanopore sequencing is generally on the way to link with nanostructural components/materials such as glass nanopipettes, nanostraws, carbon nanotube probes, and other nanomaterials [381]. By constructing channels between the intracellular and extracellular portions of the cell membrane, these nanocomponents facilitate the sequencing by enabling single-cell sampling [382]. An application of bisulfite sequencing to a single-cell level, similar to these nanocomponents, addresses inter- or intra-heterogeneity of tumor cells with significant DNA degradation [382]. To accurately identify the heterogeneity of genes in future cancer treatments, it is therefore advised to research on the combination of nanopore sequencing, nanostructure components, and bisulfite sequencing or direct sequencing.

## Conclusion and future perspective

Epigenetics is a significant gene regulator that necessitates thorough sequencing. The multi-omics-based medicine of the future will not be complete without sequencing epigenetics, particularly in the context of cancer biology. Furthermore, research and individualized, evidence-based medical services would benefit from using epigenetics as a biomarker for diagnosis and as a pharmaceutical target. The heterogeneity of cancer is influenced by epigenetics, which makes epigenetic sequencing crucial. Conventional methods have been used for sequencing up until now, but in the future, nanopore sequencing will be a more specialized method. According to earlier research, the Oxford Nanopore sequencer is the best method for advancing both genomic and epigenomic sequencing and has more advantages over rival sequencing technologies when presenting epigenetics in the multi-omics space. Moreover, Oxford Nanopore Technologies, which permits direct sequencing without the need for a lot of reagents, is better suited than any other sequencing device for exploring the roles of epigenetics in cancer heterogeneity.

In the multi-omics age, the Oxford Nanopore sequencing technique will be highly effective in presenting one arm of epigenetics and the other arm of genomics. Oxford Nanopore sequencing is a quickly developing method that is fiercely challenging Illumina's sequencing technology. Due to its reduced size and price, Oxford Nanopore sequencing is predicted to overtake Illumina sequencing technology with several advantages. Consequently, a single nanopore sequencing platform may perform epigenomics, genomics, transcriptomics, and proteomics.

Finally, future cancer medicine studies will need to take into account the incorporation of different nano-biomaterials with nanopore sequencing technologies in order to detect epigenetics in cancer in a more accurate manner. The clinical viability and delivery mechanism must be taken into account by the nano-combined sequencing procedures in addition to the incorporation of biomaterials.

## Data Availability

Data available in this review manuscript (figures and tables) have got permission for reprinting from the publishers.

## Abbreviations

5caC: 5-Carboxylcytosine
5fC: 5-Fluorocytosine
5hmC: 5-Hydroxymethylcytosine
5mC: 5-Methylcytosine
Bis/oxBis: Bisulfite/oxidative bisulfite sequencing
BSPAT: Bisulfite pattern analysis
CDB: Controlled dielectric breakdown
CIMP: CpG island methylator phenotypes
clyA: Cytolysin A
CpG islands: Cytosine followed by guanine residues
CRC: Colorectal cancer
CsgG: Curli-specific gene product G
DNMT: DNA methyltransferases
HAT: Histone acetyltransferases
HCC: Hepatocellular carcinoma
HDAC: Histone deacetylases
HDMT: Histone demethylases
HGP: Human Genome Project
hMeDIP-seq: Hydroxymethylation DNA immunoprecipitation
HMT: Histone methyltransferases
HMM: Hidden Markov model
HMM-HDP: Hidden Markov model with hierarchical Dirichlet process
KF: Klenow fragment
LncRNAs: Long noncoding RNAs
MspA: Mycobacterium smegmatis porin A
NfpA/NfpB: Nocardia farcinica peptide A/B
NGS: Next-generation sequencing
OmpF: Outer membrane protein F
OmpG: Outer membrane protein G
ONT: Oxford Nanopore Technologies
PCR: Polymerase chain reaction
PSD: Power spectral density
SAHA: Suberoyl anilide bishydroxamide
SBS: Sequence by synthesis
SGS: Second-generation sequencing
SMRT: Single-molecule real-time
SMS: Single-molecule sequencing
SNAILS: Slow nucleic acid instruments for long sequencer
SNR: Signal-to-noise ratio
T2T: Telomere-to-telomere
TAB-Seq: TET-assisted bisulfite sequencing
TAPS: TET-Assisted pyridine borane sequencing
TET: Ten–eleven translocation family proteins
TSA: Trichostatin A
TSG: Tumor suppressor genes
α-HL: α-Hemolysin

## References

[1] Watson JD, Crick FH. The structure of DNA. In: Cold spring harbor symposia on quantitative biology, vol 18. Cold Spring Harbor Laboratory Press; 1953. p. 123–31 10.1101/SQB.1953.018.01.020. 10.1101/sqb.1953.018.01.020 13168976

[2] Dahm R (2005). Friedrich Miescher and the discovery of DNA. Dev Biol.

[3] Klug A (1968). Rosalind Franklin and the discovery of the structure of DNA. Nature.

[4] Men AE, Wilson P, Siemering K, Forrest S. Sanger DNA sequencing. In: Next-generation genome sequencing: towards personalized medicine; 2008. p. 1–11. 10.1002/9783527625130.

[5] Baudhuin LM, Lagerstedt SA, Klee EW, Fadra N, Oglesbee D, Ferber MJ (2015). Confirming variants in next-generation sequencing panel testing by Sanger sequencing. J Mol Diagn.

[6] França LT, Carrilho E, Kist TB (2002). A review of DNA sequencing techniques. Q Rev Biophys.

[7] Maxam AM, Gilbert W (1977). A new method for sequencing DNA. Proc Natl Acad Sci.

[8] Tipu HN, Shabbir A (2015). Evolution of DNA sequencing. J Coll Physicians Surg Pak.

[9] Sakamoto F, Suzuki E, Fujii Y (2002). Novel approach for the effective determination of DNA scission site using the Sanger method. J Biochem Biophys Methods.

[10] Verma M, Kulshrestha S, Puri A (2016). Genome sequencing. Bioinformatics.

[11] Collins FS, McKusick VA (2001). Implications of the human genome project for medical science. JAMA.

[12] Lunshof JE, Bobe J, Aach J, Angrist M, Thakuria JV, Vorhaus DB, Hoehe MR, Church GM (2010). Personal genomes in progress: from the human genome project to the personal genome project. Dialogues Clin Neurosci.

[13] Powledge TM (2003). Human genome project completed. Genome Biol.

[14] Collins FS, Morgan M, Patrinos A (2003). The human genome project: lessons from large-scale biology. Science.

[15] Hu T, Chitnis N, Monos D, Dinh A (2021). Next-generation sequencing technologies: an overview. Hum Immunol.

[16] Miga KH, Koren S, Rhie A, Vollger MR, Gershman A, Bzikadze A, Brooks S, Howe E, Porubsky D, Logsdon GA, Schneider VA (2020). Telomere-to-telomere assembly of a complete human X chromosome. Nature.

[17] Feng Y, Zhang Y, Ying C, Wang D, Du C (2015). Nanopore-based fourth-generation DNA sequencing technology. Genom Proteom Bioinform.

[18] Göpfrich K, Judge K (2018). Decoding DNA with a pocket-sized sequencer. Biol Health.

[19] Hoenen T, Groseth A, Rosenke K, Fischer RJ, Hoenen A, Judson SD, Martellaro C, Falzarano D, Marzi A, Squires RB, Wollenberg KR (2016). Nanopore sequencing as a rapidly deployable Ebola outbreak tool. Emerg Infect Dis.

[20] Hall CL, Zascavage RR, Sedlazeck FJ, Planz JV (2020). Potential applications of nanopore sequencing for forensic analysis. Forensic Science Review.

[21] Yan Y, Wu K, Chen J, Liu H, Huang Y, Zhang Y, Xiong J, Quan W, Wu X, Liang Y, He K (2021). Rapid acquisition of high-quality SARS-CoV-2 genome via amplicon-Oxford nanopore sequencing. Virol Sin.

[22] Wang M, Fu A, Hu B, Tong Y, Liu R, Liu Z, Gu J, Xiang B, Liu J, Jiang W, Shen G (2020). Nanopore targeted sequencing for the accurate and comprehensive detection of SARS-CoV-2 and other respiratory viruses. Small.

[23] Guo M, Peng Y, Gao A, Du C, Herman JG (2019). Epigenetic heterogeneity in cancer. Biomark Res.

[24] Hoey T (2010). Drug resistance, epigenetics, and tumor cell heterogeneity. Sci Transl Med.

[25] Schatz MC (2017). Nanopore sequencing meets epigenetics. Nat Methods.

[26] Brero A, Easwaran HP, Nowak D, Grunewald I, Cremer T, Leonhardt H, Cardoso MC (2005). Methyl CpG–binding proteins induce large-scale chromatin reorganization during terminal differentiation. J Cell Biol.

[27] Ferguson LR, Tatham AL, Lin Z, Denny WA (2011). Epigenetic regulation of gene expression as an anticancer drug target. Curr Cancer Drug Targets.

[28] Beckmann ND, Karri S, Fang G, Bashir A (2014). Detecting epigenetic motifs in low coverage and metagenomics settings. BMC Bioinform.

[29] Krishna BM, Khan MA, Khan ST (2019). Next-generation sequencing (NGS) platforms: an exciting era of genome sequence analysis. Microb Genom Sustain Agroecosyst.

[30] Gupta AK, Gupta UD (2020). Next-generation sequencing, and its applications. Anim Biotechnol.

[31] Lin B, Hui J, Mao H (2021). Nanopore technology and its applications in gene sequencing. Biosensors.

[32] Hutchison CA (2007). DNA sequencing: bench to bedside and beyond. Nucleic Acids Res.

[33] Watson JD (1990). The human genome project: past, present, and future. Science.

[34] Hilgartner S (2013). Constituting large-scale biology: building a regime of governance in the early years of the human genome project. BioSocieties.

[35] Herlihy W (1991). The human genome project. Anal Chem.

[36] Goodwin S, McPherson JD, McCombie WR (2016). Coming of age: ten years of next-generation sequencing technologies. Nat Rev Genet.

[37] Schloss JA, Gibbs RA, Makhijani VB, Marziali A (2020). Cultivating DNA sequencing technology after the human genome project. Annu Rev Genom Hum Genet.

[38] Nakano K, Shiroma A, Shimoji M, Tamotsu H, Ashimine N (2017). Advantages of genome sequencing by long-read sequencer using SMRT technology in medical area. Hum Cell.

[39] Shokralla S, Spall JL, Gibson JF, Hajibabaei M (2012). Next-generation sequencing technologies for environmental DNA research. Mol Ecol.

[40] Balzer S, Malde K, Lanzén A, Sharma A, Jonassen I (2010). Characteristics of 454 pyrosequencing data—enabling realistic simulation with flowsim. Bioinformatics.

[41] Kchouk M, Gibrat JF, Elloumi M (2017). Generations of sequencing technologies: from first to next generation. Biol Med.

[42] Shuikan A, Alharbi SA, Alkhalifah DHM, Hozzein WN. High-throughput sequencing and metagenomic data analysis. In: Metagenomics-basics, methods, and applications. IntechOpen; 2019. 10.5772/intechopen.78746.

[43] Schatz MC, Delcher AL, Salzberg SL (2010). Assembly of large genomes using second-generation sequencing. Genome Res.

[44] Schadt EE, Turner S, Kasarskis A (2010). R2, a window into third-generation sequencing. Hum Mol Genet.

[45] Xiao T, Zhou W (2020). The third generation sequencing: the advanced approach to genetic diseases. Transl Pediatr.

[46] Niedringhaus TP, Milanova D, Kerby MB, Snyder MP, Barron AE (2011). The landscape of next-generation sequencing technologies. Anal Chem.

[47] McCarthy A (2010). Third generation DNA sequencing: pacific biosciences' single-molecule real-time technology. Chem Biol.

[48] Shendure J, Balasubramanian S, Church GM, Gilbert W, Rogers J, Schloss JA, Waterston RH (2017). DNA sequencing at 40: past, present, and future. Nature.

[49] Pereira R, Oliveira J, Sousa M (2020). Bioinformatics and computational tools for next-generation sequencing analysis in clinical genetics. J Clin Med.

[50] Gonzalez-Garay ML. Introduction to isoform sequencing using pacific biosciences technology (Iso-Seq). In: Transcriptomics and gene regulation. Springer; 2016. p. 141–60. 10.1007/978-94-017-7450-5_6.

[51] Koren S, Schatz MC, Walenz BP, Martin J, Howard JT, Ganapathy G, Wang Z, Rasko DA, McCombie WR, Jarvis ED, Phillippy AM (2012). Hybrid error correction and de novo assembly of single-molecule sequencing reads. Nat Biotechnol.

[52] Schloss PD, Jenior ML, Koumpouras CC, Westcott SL, Highlander SK (2016). Sequencing 16S rRNA gene fragments using the PacBio SMRT DNA sequencing system. PeerJ.

[53] Ari Ş, Arikan M. Next-generation sequencing: advantages, disadvantages, and future. In: Plant omics: trends and applications. Cham: Springer; 2016. p. 109–35. 10.1007/978-3-319-31703-8_5.

[54] Rhoads A, Au KF (2015). PacBio sequencing, and its applications. Genom Proteom Bioinform.

[55] Raley C, Munroe D, Jones K, Tsai YC, Guo Y, Tran B, Gowda S, Troyer JL, Soppet DR, Stewart C, Stephens R (2014). Preparation of next-generation DNA sequencing libraries from ultra-low amounts of input DNA: Application to single-molecule, real-time (SMRT) sequencing on the Pacific biosciences RS II. bioRxiv.

[56] Ardui S, Ameur A, Vermeesch JR, Hestand MS (2018). Single-molecule real-time (SMRT) sequencing comes of age: applications and utilities for medical diagnostics. Nucleic Acids Res.

[57] Eid J, Fehr A, Gray J, Luong K, Lyle J, Otto G, Peluso P, Rank D, Baybayan P, Bettman B, Bibillo A (2009). Real-time DNA sequencing from single polymerase molecules. Science.

[58] Niedringhaus TP, Milanova D, Kerby MB, Snyder MP, Barron AE (2011). Landscape of next-generation sequencing technologies. Anal Chem.

[59] Li Y (2021). Modern epigenetics methods in biological research. Methods.

[60] Ludwig CH, Bintu L (2019). Mapping chromatin modifications at the single-cell level. Development.

[61] Hagan JT, Sheetz BS, Bandara YND, Karawdeniya BI, Morris MA, Chevalier RB, Dwyer JR (2020). Chemically tailoring nanopores for single-molecule sensing and glycomics. Anal Bioanal Chem.

[62] Desai TA, Hansford DJ, Kulinsky L, Nashat AH, Rasi G, Tu J, Wang Y, Zhang M, Ferrari M (1999). Nanopore technology for biomedical applications. Biomed Microdevice.

[63] Lu H, Giordano F, Ning Z (2016). Oxford Nanopore MinION sequencing and genome assembly. Genom Proteom Bioinform.

[64] Jain M, Olsen HE, Paten B, Akeson M (2016). The Oxford Nanopore MinION: delivery of nanopore sequencing to the genomics community. Genome Biol.

[65] Laver T, Harrison J, O’Neill PA, Moore K, Farbos A, Paszkiewicz K, Studholme DJ (2015). Assessing the performance of the Oxford nanopore technologies minion. Biomol Detect Quantif.

[66] Bowden R, Davies RW, Heger A, Pagnamenta AT, de Cesare M, Oikkonen LE, Parkes D, Freeman C, Dhalla F, Patel SY, Popitsch N (2019). Sequencing of human genomes with nanopore technology. Nat Commun.

[67] Maitra RD, Kim J, Dunbar WB (2012). Recent advances in nanopore sequencing. Electrophoresis.

[68] Wong IY, Bhatia SN, Toner M (2013). Nanotechnology: emerging tools for biology and medicine. Genes Dev.

[69] Nehra A, Ahlawat S, Singh KP (2019). A biosensing expedition of nanopore: a review. Sens Actuators B Chem.

[70] Karawdeniya BI, Bandara YND, Nichols JW, Chevalier RB, Hagan JT, Dwyer JR (2019). Challenging nanopores with analyte scope and environment. J Anal Test.

[71] Kowalczyk SW, Blosser TR, Dekker C (2011). Biomimetic nanopores: learning from and about nature. Trends Biotechnol.

[72] Watson MA, Cockroft SL (2016). Man-made molecular machines: membrane-bound. Chem Soc Rev.

[73] Norris AL, Workman RE, Fan Y, Eshleman JR, Timp W (2016). Nanopore sequencing detects structural variants in cancer. Cancer Biol Ther.

[74] Ko J, Bhagwat N, Black T, Yee SS, Na YJ, Fisher S, Kim J, Carpenter EL, Stanger BZ, Issadore D (2018). miRNA profiling of magnetic nanopore–isolated extracellular vesicles for the diagnosis of pancreatic cancer. Can Res.

[75] Euskirchen P, Bielle F, Labreche K, Kloosterman WP, Rosenberg S, Daniau M, Schmitt C, Masliah-Planchon J, Bourdeaut F, Dehais C, Marie Y (2017). Same-day genomic and epigenomic diagnosis of brain tumors using real-time nanopore sequencing. Acta Neuropathol.

[76] Hong M, Tao S, Zhang L, Diao LT, Huang X, Huang S, Xie SJ, Xiao ZD, Zhang H (2020). RNA sequencing: new technologies and applications in cancer research. J Hematol Oncol.

[77] Jain M, Koren S, Miga KH, Quick J, Rand AC, Sasani TA, Tyson JR, Beggs AD, Dilthey AT, Fiddes IT, Malla S (2018). Nanopore sequencing and assembly of a human genome with ultra-long reads. Nat Biotechnol.

[78] Schweiger MR, Kerick M, Timmermann B, Isau M (2011). The power of NGS technologies to delineate the genome organization in cancer: from mutations to structural variations and epigenetic alterations. Cancer Metastasis Rev.

[79] Ku CS, Roukos DH (2013). From next-generation sequencing to nanopore sequencing technology: paving the way to personalized genomic medicine. Expert Rev Med Devices.

[80] Brown CG, Clarke J (2016). Nanopore development at oxford nanopore. Nat Biotechnol.

[81] Neher E, Sakmann B (1976). Single-channel currents recorded from membrane of denervated frog muscle fibers. Nature.

[82] Wanunu M (2012). Nanopores: a journey towards DNA sequencing. Phys Life Rev.

[83] Deamer DW, Nichols JW (1989). Proton flux mechanisms in model and biological membranes. J Membr.

[84] Bayley H (2015). Nanopore sequencing: from imagination to reality. Clin Chem.

[85] Branton D, Deamer DW. Nanopore sequencing: an introduction. World Scientific. 2019.

[86] Ma L, Cockroft SL (2010). Biological nanopores for single-molecule biophysics. ChemBioChem.

[87] Agah S, Zheng M, Pasquali M, Kolomeisky AB (2016). DNA sequencing by nanopores: advances and challenges. J Phys D Appl Phys.

[88] Schneider GF, Dekker C (2012). DNA sequencing with nanopores. Nat Biotechnol.

[89] Steinbock LJ, Radenovic A (2015). The emergence of nanopores in next-generation sequencing. Nanotechnology.

[90] Rusk N (2014). Nanopores read long genomic DNA. Nat Methods.

[91] Galdiero S, Gouaux E (2004). High-resolution crystallographic studies of α-hemolysin–phospholipid complexes define heptamer–lipid head group interactions: Implication for understanding protein-lipid interactions. Protein Sci.

[92] He M, Chi X, Ren J. Applications of Oxford nanopore sequencing in Schizosaccharomyces pombe. In: Yeast protocols; 2021. p. 97–116. 10.1007/978-1-0716-0868-5_9. 10.1007/978-1-0716-0868-5_9 32889716

[93] Celaya G, Perales-Calvo J, Muga A, Moro F, Rodriguez-Larrea D (2017). Label-free, multiplexed, single-molecule analysis of protein–DNA complexes with nanopores. ACS Nano.

[94] Mikheyev AS, Tin MM (2014). A first look at the Oxford Nanopore MinION sequencer. Mol Ecol Resour.

[95] Xie S, Leung AWS, Zheng Z, Zhang D, Xiao C, Luo R, Luo M, Zhang S (2021). The applications and potentials of nanopore sequencing in the (epi) genome and (epi) transcriptome era. The Innovation.

[96] Chu Y, Corey DR (2012). RNA sequencing: platform selection, experimental design, and data interpretation. Nucleic Acid Ther.

[97] Zhao L, Zhang H, Kohnen MV, Prasad KV, Gu L, Reddy AS (2019). Analysis of transcriptome and epitranscriptome in plants using PacBio Iso-Seq and nanopore-based direct RNA sequencing. Front Genet.

[98] Garalde DR, Snell EA, Jachimowicz D, Sipos B, Lloyd JH, Bruce M, Pantic N, Admassu T, James P, Warland A, Jordan M (2018). Highly parallel direct RNA sequencing on an array of nanopores. Nat Methods.

[99] Kang X, Alibakhshi MA, Wanunu M (2019). Improved bilayer membrane stability for nanopore sensing applications. Biophys J.

[100] Leggett RM, Clark MD (2017). A world of opportunities with nanopore sequencing. J Exp Bot.

[101] Logsdon GA, Vollger MR, Eichler EE (2020). Long-read human genome sequencing and its applications. Nat Rev Genet.

[102] Loit K, Adamson K, Bahram M, Puusepp R, Anslan S, Kiiker R, Drenkhan R, Tedersoo L (2019). Relative performance of MinION (Oxford Nanopore Technologies) versus Sequel (Pacific Biosciences) third-generation sequencing instruments in identification of agricultural and forest fungal pathogens. Appl Environ Microbiol.

[103] Check Hayden E (2012). Nanopore genome sequencer makes its debut. Nature News.

[104] Andrews TS, Kiselev VY, McCarthy D, Hemberg M (2021). Tutorial: guidelines for the computational analysis of single-cell RNA sequencing data. Nat Protoc.

[105] Park PJ (2009). ChIP–seq: advantages and challenges of a maturing technology. Nat Rev Genet.

[106] Rauluseviciute I, Drabløs F, Rye MB (2019). DNA methylation data by sequencing: experimental approaches and recommendations for tools and pipelines for data analysis. Clin Epigenet.

[107] Carson S, Wanunu M (2015). Challenges in DNA motion control and sequence readout using nanopore devices. Nanotechnology.

[108] Branton D, Deamer DW, Marziali A, Bayley H, Benner SA, Butler T, Di Ventra M, Garaj S, Hibbs A, Huang X, Jovanovich SB (2010). The potential and challenges of nanopore sequencing. Nanosci Technol Collect Rev Nat J.

[109] Vogel R, Pal AK, Jambhrunkar S, Patel P, Thakur SS, Reátegui E, Parekh HS, Saá P, Stassinopoulos A, Broom MF (2017). High-resolution single-particle zeta potential characterization of biological nanoparticles using tunable resistive pulse sensing. Sci Rep.

[110] Stanley S (2014). Biological nanoparticles and their influence on organisms. Curr Opin Biotechnol.

[111] Stark WJ (2011). Nanoparticles in biological systems. Angew Chem Int Ed.

[112] Schmidt J (2016). Membrane platforms for biological nanopore sensing and sequencing. Curr Opin Biotechnol.

[113] May M, Wang HM, Akid R (2010). Effects of the addition of inorganic nanoparticles on the adhesive strength of a hybrid sol-gel epoxy system. Int J Adhes Adhes.

[114] Cumbal L, Greenleaf J, Leun D, SenGupta AK (2003). Polymer supported inorganic nanoparticles: characterization and environmental applications. React Funct Polym.

[115] Lee MH, Kumar A, Park KB, Cho SY, Kim HM, Lim MC, Kim YR, Kim KB (2014). A low-noise solid-state nanopore platform based on a highly insulating substrate. Sci Rep.

[116] Haque F, Li J, Wu HC, Liang XJ, Guo P (2013). Solid-state and biological nanopore for real-time sensing of single chemical and sequencing of DNA. Nano Today.

[117] Iqbal SM, Akin D, Bashir R (2007). Solid-state nanopore channels with DNA selectivity. Nat Nanotechnol.

[118] Chen Q, Liu Z (2019). Fabrication and applications of solid-state nanopores. Sensors.

[119] Miles BN, Ivanov AP, Wilson KA, Doğan F, Japrung D, Edel JB (2013). Single-molecule sensing with solid-state nanopores: novel materials, methods, and applications. Chem Soc Rev.

[120] Di Fiori N, Squires A, Bar D, Gilboa T, Moustakas TD, Meller A (2013). Optoelectronic control of surface charge and translocation dynamics in solid-state nanopores. Nat Nanotechnol.

[121] Rollings R, Graef E, Walsh N, Nandivada S, Benamara M, Li J (2015). The effects of geometry and stability of solid-state nanopores on detecting single DNA molecules. Nanotechnology.

[122] Larkin J, Henley R, Bell DC, Cohen-Karni T, Rosenstein JK, Wanunu M (2013). Slow DNA transport through nanopores in hafnium oxide membranes. ACS Nano.

[123] Li J, Yu D, Zhao Q (2016). Solid-state nanopore-based DNA single-molecule detection and sequencing. Microchim Acta.

[124] Chen W, Liu GC, Ouyang J, Gao MJ, Liu B, Zhao YD (2017). Graphene nanopores toward DNA sequencing: a review of experimental aspects. Sci China Chem.

[125] Kudr J, Skalickova S, Nejdl L, Moulick A, Ruttkay-Nedecky B, Adam V, Kizek R (2015). Fabrication of solid-state nanopores and its perspectives. Electrophoresis.

[126] Liu K, Feng J, Kis A, Radenovic A (2014). Atomically thin molybdenum disulfide nanopores with high sensitivity for DNA translocation. ACS Nano.

[127] Xiong M, Graf M, Athreya N, Radenovic A, Leburton JP (2020). Microscopic detection analysis of single molecules in MoS2 membrane nanopores. ACS Nano.

[128] Boutilier MS, Jang D, Idrobo JC, Kidambi PR, Hadjiconstantinou NG, Karnik R (2017). Molecular sieving across centimeter-scale single-layer nanoporous graphene membranes. ACS Nano.

[129] Yuan Z, Liu Y, Dai M, Yi X, Wang C (2020). Controlling DNA translocation through solid-state nanopores. Nanoscale Res Lett.

[130] Thakur M, Macha M, Chernev A, Graf M, Lihter M, Deen J, Tripathi M, Kis A, Radenovic A (2020). Wafer-scale fabrication of nanopore devices for single-molecule DNA biosensing using MoS2. Small Methods.

[131] Schneider GF, Kowalczyk SW, Calado VE, Pandraud G, Zandbergen HW, Vandersypen LM, Dekker C (2010). DNA translocation through graphene nanopores. Nano Lett.

[132] Kowalczyk SW, Hall AR, Dekker C (2010). Detection of local protein structures along DNA using solid-state nanopores. Nano Lett.

[133] Wasfi A, Awwad F, Ayesh AI (2018). Graphene-based nanopore approaches for DNA sequencing: a literature review. Biosens Bioelectron.

[134] Cao J, Jia W, Zhang J, Xu X, Yan S, Wang Y, Zhang P, Chen HY, Huang S (2019). Giant single-molecule chemistry events observed from a tetrachloroaurate (III) embedded Mycobacterium smegmatis porin A nanopore. Nat Commun.

[135] Manrao EA, Derrington IM, Pavlenok M, Niederweis M, Gundlach JH (2011). Nucleotide discrimination with DNA immobilized in the MspA nanopore. PLoS ONE.

[136] Cao B, Zhao Y, Kou Y, Ni D, Zhang XC, Huang Y (2014). Structure of the nonameric bacterial amyloid secretion channel. Proc Natl Acad Sci.

[137] Carter JM, Hussain S (2017). Robust long-read native DNA sequencing using the ONT CsgG nanopore system. Wellcome Open Res.

[138] Goyal P, Krasteva PV, Van Gerven N, Gubellini F, Van den Broeck I, Troupiotis-Tsaïlaki A, Jonckheere W, Péhau-Arnaudet G, Pinkner JS, Chapman MR, Hultgren SJ (2014). Structural and mechanistic insights into the bacterial amyloid secretion channel CsgG. Nature.

[139] Stoddart D, Maglia G, Mikhailova E, Heron AJ, Bayley H (2010). Multiple base-recognition sites in a biological nanopore: two heads are better than one. Angew Chem.

[140] Van der Verren SE, Van Gerven N, Jonckheere W, Hambley R, Singh P, Kilgour J, Jordan M, Wallace EJ, Jayasinghe L, Remaut H (2020). A dual-constriction biological nanopore resolves homonucleotide sequences with high fidelity. Nat Biotechnol.

[141] Crnković A, Senko M, Anderluh G (2021). Biological nanopores. Engineering on demand. Life.

[142] Ying YL, Cao C, Long YT (2014). Single-molecule analysis by biological nanopore sensors. Analyst.

[143] Yoo H, Jo H, Oh SS (2020). Detection and beyond: challenges and advances in aptamer-based biosensors. Mater Adv.

[144] Howorka S (2017). Building membrane nanopores. Nat Nanotechnol.

[145] Fyta M (2015). Threading DNA through nanopores for biosensing applications. J Phys Condens Matter.

[146] Lu B, Albertorio F, Hoogerheide DP, Golovchenko JA (2011). Origins and consequences of velocity fluctuations during DNA passage through a nanopore. Biophys J.

[147] Tan CS (2018). Detection of DNA base modifications by biological nanopores.

[148] Kulkarni SK, Kulkarni SK (2015). Nanotechnology: principles and practices.

[149] Healy K (2007). Nanopore-based single-molecule DNA analysis. Future Med.

[150] Fyta M, Melchionna S, Succi S (2011). Translocation of biomolecules through solid-state nanopores: theory meets experiments. J Polym Sci Part B Polym Phys.

[151] Luan B, Stolovitzky G, Martyna G (2012). Slowing and controlling the translocation of DNA in a solid-state nanopore. Nanoscale.

[152] Wang J, Yang J, Ying YL, Long YT (2019). Nanopore-based confined spaces for single-molecular analysis. Chem Asian J.

[153] Reiner JE, Balijepalli A, Robertson JW, Drown BS, Burden DL, Kasianowicz JJ (2012). The effects of diffusion on an exonuclease/nanopore-based DNA sequencing engine. J Chem Phys.

[154] Dillingham MS, Kowalczykowski SC (2008). RecBCD enzyme and the repair of double-stranded DNA breaks. Microbiol Mol Biol Rev.

[155] Gyarfas B, Olasagasti F, Benner S, Garalde D, Lieberman KR, Akeson M (2009). Mapping the position of DNA polymerase-bound DNA templates in a nanopore at 5 Å resolution. ACS Nano.

[156] Cherf GM, Lieberman KR, Rashid H, Lam CE, Karplus K, Akeson M (2012). Automated forward and reverse ratcheting of DNA in a nanopore at 5-Å precision. Nat Biotechnol.

[157] Lieberman KR, Cherf GM, Doody MJ, Olasagasti F, Kolodji Y, Akeson M (2010). Processive replication of single DNA molecules in a nanopore catalyzed by phi29 DNA polymerase. J Am Chem Soc.

[158] Cho MW, Richards OC, Dmitrieva TM, Agol V, Ehrenfeld E (1993). RNA duplex unwinding activity of poliovirus RNA-dependent RNA polymerase 3Dpol. J Virol.

[159] Caldwell CC, Spies M (2017). Helicase SPRNTing through the nanopore. Proc Natl Acad Sci.

[160] Craig JM, Laszlo AH, Nova IC, Brinkerhoff H, Noakes MT, Baker KS, Bowman JL, Higinbotham HR, Mount JW, Gundlach JH (2019). Determining the effects of DNA sequence on Hel308 helicase translocation along single-stranded DNA using nanopore tweezers. Nucleic Acids Res.

[161] Goto Y, Yanagi I, Matsui K, Yokoi T, Takeda KI (2016). Integrated solid-state nanopore platform for nanopore fabrication via dielectric breakdown, DNA-speed deceleration, and noise reduction. Sci Rep.

[162] Aksimentiev A, Heng JB, Timp G, Schulten K (2004). Microscopic kinetics of DNA translocation through synthetic nanopores. Biophys J.

[163] Avdoshenko SM, Nozaki D, Gomes da Rocha C, González JW, Lee MH, Gutierrez R, Cuniberti G (2013). Dynamic and electronic transport properties of DNA translocation through graphene nanopores. Nano Lett.

[164] Kawano R, Schibel AE, Cauley C, White HS (2009). Controlling the translocation of single-stranded DNA through α-hemolysin ion channels using viscosity. Langmuir.

[165] Ali M, Yameen B, Cervera J, Ramirez P, Neumann R, Ensinger W, Knoll W, Azzaroni O (2010). Layer-by-layer assembly of polyelectrolytes into ionic current rectifying solid-state nanopores: insights from theory and experiment. J Am Chem Soc.

[166] Akahori R, Haga T, Hatano T, Yanagi I, Ohura T, Hamamura H, Iwasaki T, Yokoi T, Anazawa T (2014). Slowing single-stranded DNA translocation through a solid-state nanopore by decreasing the nanopore diameter. Nanotechnology.

[167] Tu B, Bai S, Lu B, Fang Q (2018). Conic shapes have higher sensitivity than cylindrical ones in nanopore DNA sequencing. Sci Rep.

[168] Goto Y, Akahori R, Yanagi I, Takeda KI (2020). Solid-state nanopores towards single-molecule DNA sequencing. J Hum Genet.

[169] Goto Y, Matsui K, Yanagi I, Takeda KI (2019). Silicon nitride nanopore created by dielectric breakdown with a divalent cation: deceleration of translocation speed and identification of single nucleotides. Nanoscale.

[170] Kowalczyk SW, Wells DB, Aksimentiev A, Dekker C (2012). Slowing down DNA translocation through a nanopore in lithium chloride. Nano Lett.

[171] Yan H, Zhou D, Shi B, Zhang Z, Tian H, Yu L, Wang Y, Guan X, Wang Z, Wang D (2019). Slowing down DNA translocation velocity using a LiCl salt gradient and nanofiber mesh. Eur Biophys J.

[172] Vu T, Borgesi J, Soyring J, D'Alia M, Davidson SL, Shim J (2019). Employing LiCl salt gradient in the wild-type α-hemolysin nanopore to slow down DNA translocation and detect methylated cytosine. Nanoscale.

[173] Uplinger J, Thomas B, Rollings R, Fologea D, McNabb D, Li J (2012). K+, N a+, and M g2+ on DNA translocation in silicon nitride nanopores. Electrophoresis.

[174] Fragasso A, Schmid S, Dekker C (2020). Comparing current noise in biological and solid-state nanopores. ACS Nano.

[175] Liang S, Xiang F, Tang Z, Nouri R, He X, Dong M, Guan W (2020). Noise in nanopore sensors: sources, models, reduction, and benchmarking. Nanotechnol Precis Eng.

[176] Gilboa T, Meller A (2015). Optical sensing and analyte manipulation in solid-state nanopores. Analyst.

[177] Hartel AJ, Shekar S, Ong P, Schroeder I, Thiel G, Shepard KL (2019). High bandwidth approaches in nanopore and ion channel recordings-a tutorial review. Anal Chim Acta.

[178] Lee K, Park KB, Kim HJ, Yu JS, Chae H, Kim HM, Kim KB (2018). Recent progress in solid-state nanopores. Adv Mater.

[179] Rodríguez-Manzo JA, Puster M, Nicolaï A, Meunier V, Drndic M (2015). DNA translocation in nanometer-thick silicon nanopores. ACS Nano.

[180] Danda G, Drndić M (2019). Two-dimensional nanopores and nanoporous membranes for ion and molecule transport. Curr Opin Biotechnol.

[181] Beamish E, Kwok H, Tabard-Cossa V, Godin M (2012). Precise control of the size and noise of solid-state nanopores using high electric fields. Nanotechnology.

[182] Rang FJ, Kloosterman WP, de Ridder J (2018). From squiggle to basepair: computational approaches for improving nanopore sequencing read accuracy. Genome Biol.

[183] Kawano R (2018). Nanopore decoding of oligonucleotides in DNA computing. Biotechnol J.

[184] Senol Cali D, Kim JS, Ghose S, Alkan C, Mutlu O (2019). Nanopore sequencing technology and tools for genome assembly: computational analysis of the current state, bottlenecks, and future directions. Brief Bioinform.

[185] Manrao EA, Derrington IM, Laszlo AH, Langford KW, Hopper MK, Gillgren N, Pavlenok M, Niederweis M, Gundlach JH (2012). Reading DNA at single-nucleotide resolution with a mutant MspA nanopore and phi29 DNA polymerase. Nat Biotechnol.

[186] Deamer D, Akeson M, Branton D (2016). Three decades of nanopore sequencing. Nat Biotechnol.

[187] Prall TM, Neumann EK, Karl JA, Shortreed CG, Baker DA, Bussan HE, Wiseman RW, O’Connor DH (2021). Consistent ultra-long DNA sequencing with automated slow pipetting. BMC Genom.

[188] McCoy RC, Taylor RW, Blauwkamp TA, Kelley JL, Kertesz M, Pushkarev D, Petrov DA, Fiston-Lavier AS (2014). Illumina TruSeq synthetic long-reads empower de novo assembly and resolve complex highly-repetitive transposable elements. PLoS ONE.

[189] Mason CE, Afshinnekoo E, Tighe S, Wu S, Levy S (2017). International standards for genomes, transcriptomes, and metagenomes. J Biomol Tech JBT.

[190] Gong L, Wong CH, Idol J, Ngan CY, Wei CL (2019). Ultra-long read sequencing for whole genomic DNA analysis. JoVE J Vis Exp.

[191] Weirather JL, de Cesare M, Wang Y, Piazza P, Sebastiano V, Wang XJ, Buck D, Au KF (2017). Comprehensive comparison of Pacific Biosciences and Oxford Nanopore Technologies and their applications to transcriptome analysis. F1000Research.

[192] Nurk S, Koren S, Rhie A, Rautiainen M, Bzikadze AV, Mikheenko A, Vollger MR, Altemose N, Uralsky L, Gershman A, Aganezov S (2021). The complete sequence of a human genome. bioRxiv.

[193] Levy-Sakin M, Pastor S, Mostovoy Y, Li L, Leung AK, McCaffrey J, Young E, Lam ET, Hastie AR, Wong KH, Chung CY (2019). Genome maps across 26 human populations reveal population-specific patterns of structural variation. Nat Commun.

[194] Cooper DN, Bacolla A, Férec C, Vasquez KM, Kehrer-Sawatzki H, Chen JM (2011). On the sequence-directed nature of human gene mutation: the role of genomic architecture and the local DNA sequence environment in mediating gene mutations underlying human inherited disease. Hum Mutat.

[195] Magi A, Semeraro R, Mingrino A, Giusti B, D’Aurizio R (2018). Nanopore sequencing data analysis: state of the art, applications, and challenges. Brief Bioinform.

[196] Silvestre-Ryan J, Holmes I (2021). Pair consensus decoding improves accuracy of neural network basecallers for nanopore sequencing. Genome Biol.

[197] Rosenstein JK, Lemay SG, Shepard KL (2015). Single-molecule bioelectronics. Wiley Interdiscip Rev Nanomed Nanobiotechnol.

[198] Xue L, Yamazaki H, Ren R, Wanunu M, Ivanov AP, Edel JB (2020). Solid-state nanopore sensors. Nat Rev Mater.

[199] Ma T, Janot JM, Balme S (2020). Track-etched nanopore/membrane: from fundamental to applications. Small Methods.

[200] Balme S, Lepoitevin M, Bechelany M, Janot JM. Hybrid biological/artificial nanopore. In: Physics, chemistry, and applications of nanostructures: proceedings of international conference nanomeeting; 2015. p. 454–6. 10.1142/9789814696524_0112.

[201] Bechelany M, Balme S, Miele P (2015). Atomic layer deposition of biobased nanostructured interfaces for energy, environmental and health applications. Pure Appl Chem.

[202] Lepoitevin M, Ma T, Bechelany M, Janot JM, Balme S (2017). Functionalization of single solid-state nanopores to mimic biological ion channels: a review. Adv Coll Interface Sci.

[203] Plesivkova D, Richards R, Harbison S (2019). A review of the potential of the MinION™ single-molecule sequencing system for forensic applications. Wiley Interdiscip Rev Forensic Science.

[204] Kono N, Arakawa K (2019). Nanopore sequencing: review of potential applications in functional genomics. Dev Growth Differ.

[205] Chaisson MJ, Tesler G (2012). Mapping single-molecule sequencing reads using basic local alignment with successive refinement (BLASR): application and theory. BMC Bioinform.

[206] Mardis ER (2017). DNA sequencing technologies: 2006–2016. Nat Protoc.

[207] Petersen LM, Martin IW, Moschetti WE, Kershaw CM, Tsongalis GJ (2019). Third-generation sequencing in the clinical laboratory: exploring the advantages and challenges of nanopore sequencing. J Clin Microbiol.

[208] Raza K, Qazi S. Nanopore sequencing technology and Internet of living things: a big hope for U-healthcare. In: Sensors for health monitoring. Academic Press; 2019. p. 95–116. 10.1016/B978-0-12-819361-7.00005-1.

[209] Xu L, Seki M (2020). Recent advances in the detection of base modifications using the Nanopore sequencer. J Hum Genet.

[210] Sedlazeck FJ, Rescheneder P, Smolka M, Fang H, Nattestad M, Von Haeseler A, Schatz MC (2018). Accurate detection of complex structural variations using single-molecule sequencing. Nat Methods.

[211] Thayer A (2016). Illumina sues Oxford Nano. Chem Eng News.

[212] Oxford Nanopore bests PacBio. Nat Biotechnol. 2019;37:333–9. 10.1038/s41587-019-0098-y 30940945

[213] Sutton JM, Millwood JD, Fierst JL. Optimizing experimental design for genome sequencing and assembly with Oxford Nanopore Technologies. bioRxiv, p. 2020–05. 2021. 10.46471/gigabyte.27. 10.46471/gigabyte.27 PMC9650304 36824342

[214] Voelkerding KV, Dames SA, Durtschi JD (2009). Next-generation sequencing: from basic research to diagnostics. Clin Chem.

[215] Lopez R, Chen YJ, Ang SD, Yekhanin S, Makarychev K, Racz MZ, Seelig G, Strauss K, Ceze L (2019). DNA assembly for nanopore data storage readout. Nat Commun.

[216] Ciuffreda L, Rodríguez-Pérez H, Flores C (2021). Nanopore sequencing and its application to the study of microbial communities. Comput Struct Biotechnol J.

[217] Lebrigand K, Magnone V, Barbry P, Waldmann R (2020). High throughput error-corrected Nanopore single-cell transcriptome sequencing. Nat Commun.

[218] Li R, Xie M, Dong N, Lin D, Yang X, Wong MHY, Chan EWC, Chen S (2018). Efficient generation of complete sequences of MDR-encoding plasmids by rapid assembly of MinION barcoding sequencing data. Gigascience.

[219] Di Costanzo A, Del Gaudio N, Migliaccio A, Altucci L (2014). Epigenetic drugs against cancer: an evolving landscape. Arch Toxicol.

[220] Cossío FP, Esteller M, Berdasco M (2020). Towards a more precise therapy in cancer: exploring epigenetic complexity. Curr Opin Chem Biol.

[221] Neganova ME, Klochkov SG, Aleksandrova YR, Aliev G. Histone modifications in epigenetic regulation of cancer: perspectives and achieved progress. In: Seminars in cancer biology. Academic Press; 2020. 10.1016/j.semcancer.2020.07.015. 10.1016/j.semcancer.2020.07.015 32814115

[222] Berdasco M, Esteller M (2013). Genetic syndromes caused by mutations in epigenetic genes. Hum Genet.

[223] Zhao L, Duan YT, Lu P, Zhang ZJ, Zheng XK, Wang JL, Feng WS (2018). Epigenetic targets and their inhibitors in cancer therapy. Curr Top Med Chem.

[224] Tomaselli D, Lucidi A, Rotili D, Mai A (2020). Epigenetic polypharmacology: a new frontier for epi-drug discovery. Med Res Rev.

[225] Kumar S, Chinnusamy V, Mohapatra T (2018). Epigenetics of modified DNA bases: 5-methylcytosine and beyonds. Front Genet.

[226] Yu M, Hon GC, Szulwach KE, Song CX, Zhang L, Kim A, Li X, Dai Q, Shen Y, Park B, Min JH (2012). Base-resolution analysis of 5-hydroxymethylcytosine in the mammalian genome. Cell.

[227] Moore LD, Le T, Fan G (2013). DNA methylation and its basic function. Neuropsychopharmacology.

[228] Liyanage VR, Jarmasz JS, Murugeshan N, Del Bigio MR, Rastegar M, Davie JR (2014). DNA modifications: function and applications in normal and disease states. Biology.

[229] Baylin SB (2005). DNA methylation and gene silencing in cancer. Nat Clin Pract Oncol.

[230] Nephew KP, Huang THM (2003). Epigenetic gene silencing in cancer initiation and progression. Cancer Lett.

[231] Raynal NJM, Si J, Taby RF, Gharibyan V, Ahmed S, Jelinek J, Estécio MR, Issa JPJ (2012). DNA methylation does not stably lock gene expression but instead serves as a molecular mark for gene silencing memory. Can Res.

[232] Cedar H, Bergman Y (2009). Linking DNA methylation and histone modification: patterns and paradigms. Nat Rev Genet.

[233] Carter B, Zhao K (2021). The epigenetic basis of cellular heterogeneity. Nat Rev Genet.

[234] Feldman N, Gerson A, Fang J, Li E, Zhang Y, Shinkai Y, Cedar H, Bergman Y (2006). G9a-mediated irreversible epigenetic inactivation of Oct-3/4 during early embryogenesis. Nat Cell Biol.

[235] Epsztejn-Litman S, Feldman N, Abu-Remaileh M, Shufaro Y, Gerson A, Ueda J, Deplus R, Fuks F, Shinkai Y, Cedar H, Bergman Y (2008). De novo DNA methylation promoted by G9a prevents reprogramming of embryonically silenced genes. Nat Struct Mol Biol.

[236] Olkhov-Mitsel E, Bapat B (2012). Strategies for discovery and validation of methylated and hydroxymethylated DNA biomarkers. Cancer Med.

[237] Richa R, Sinha RP (2014). Hydroxymethylation of DNA: an epigenetic marker. EXCLI J.

[238] Bian K, Lenz SA, Tang Q, Chen F, Qi R, Jost M, Drennan CL, Essigmann JM, Wetmore SD, Li D (2019). DNA repair enzymes ALKBH2, ALKBH3, and AlkB oxidize 5-methylcytosine to 5-hydroxymethylcytosine, 5-formylcytosine, and 5-carboxylcytosine in vitro. Nucleic Acids Res.

[239] Hu H, Shu M, He L, Yu X, Liu X, Lu Y, Chen Y, Miao X, Chen X (2017). Epigenomic landscape of 5-hydroxymethylcytosine reveals its transcriptional regulation of lncRNAs in colorectal cancer. Br J Cancer.

[240] Aristizabal MJ, Anreiter I, Halldorsdottir T, Odgers CL, McDade TW, Goldenberg A, Mostafavi S, Kobor MS, Binder EB, Sokolowski MB, O’Donnell KJ (2020). Biological embedding of experience: a primer on epigenetics. Proc Natl Acad Sci.

[241] El-Osta ASSAM, Wolffe AP (2001). DNA methylation and histone deacetylation in the control of gene expression: basic biochemistry to human development and disease. Gene Expr J Liver Res.

[242] Majumder S, Kutay H, Datta J, Summers D, Jacob ST, Ghoshal K (2006). Epigenetic regulation of metallothionein-i gene expression: differential regulation of methylated and unmethylated promoters by DNA methyltransferases and methyl CpG binding proteins. J Cell Biochem.

[243] Saito Y, Kanai Y, Sakamoto M, Saito H, Ishii H, Hirohashi S (2001). Expression of mRNA for DNA methyltransferases and methyl-CpG–binding proteins and DNA methylation status on CpG islands and pericentromeric satellite regions during human hepatocarcinogenesis. Hepatology.

[244] Li LC (2014). 1, Chromatin remodeling by the small RNA machinery in mammalian cells. Epigenetics.

[245] Li X, Fu XD (2019). Chromatin-associated RNAs as facilitators of functional genomic interactions. Nat Rev Genet.

[246] Holoch D, Moazed D (2015). RNA-mediated epigenetic regulation of gene expression. Nat Rev Genet.

[247] Dawson MA, Kouzarides T (2012). Cancer epigenetics: from mechanism to therapy. Cell.

[248] Song J, Pfeifer GP (2016). Are there specific readers of oxidized 5-methylcytosine bases?. BioEssays.

[249] Pfeifer GP, Szabó PE, Song J (2020). Protein interactions at oxidized 5-methylcytosine bases. J Mol Biol.

[250] Zhou T, Xiong J, Wang M, Yang N, Wong J, Zhu B, Xu RM (2014). Structural basis for hydroxymethylcytosine recognition by the SRA domain of UHRF2. Mol Cell.

[251] Hamidi T, Singh AK, Chen T (2015). Genetic alterations of DNA methylation machinery in human diseases. Epigenomics.

[252] Ong CT, Corces VG (2014). CTCF: an architectural protein bridging genome topology and function. Nat Rev Genet.

[253] Hillyar C, Rallis KS, Varghese J (2020). Advances in epigenetic cancer therapeutics. Cureus.

[254] Muacevic A, Adler J, Hillyar C, Rallis K, Varghese J (2021). Advances in epigenetic cancer therapeutics. Cureus.

[255] Xu H, Liu L, Li W, Zou D, Yu J, Wang L, Wong CC (2021). Transcription factors in colorectal cancer: molecular mechanism and therapeutic implications. Oncogene.

[256] Boot A, Oosting J, van Eendenburg JD, Kuppen PJ, Morreau H, van Wezel T (2017). Methylation associated transcriptional repression of ELOVL5 in novel colorectal cancer cell lines. PLoS ONE.

[257] Weisenberger DJ, Liang G, Lenz HJ (2018). DNA methylation aberrancies delineate clinically distinct subsets of colorectal cancer and provide novel targets for epigenetic therapies. Oncogene.

[258] Huang G, Zhang J, Gong L, Liu D, Wang X, Chen Y, Guo S (2021). Specific lung squamous cell carcinoma prognosis-subtype distinctions based on DNA methylation patterns. Med Sci Monit Int Med J Exp Clin Res.

[259] Um SW, Kim HK, Kim Y, Lee BB, Kim D, Han J, Kim H, Shim YM, Kim DH (2017). Bronchial biopsy specimen as a surrogate for DNA methylation analysis in inoperable lung cancer. Clin Epigenet.

[260] Dong Z, Cui H (2019). Epigenetic modulation of metabolism in glioblastoma. Semin Cancer Biol.

[261] Stroud H, Feng S, Kinney SM, Pradhan S, Jacobsen SE (2011). 5-Hydroxymethylcytosine is associated with enhancers and gene bodies in human embryonic stem cells. Genome Biol.

[262] Bochtler M, Kolano A, Xu GL (2017). DNA demethylation pathways: additional players and regulators. BioEssays.

[263] Kohli RM, Zhang Y (2013). TET enzymes, TDG, and the dynamics of DNA demethylation. Nature.

[264] Hackett JA, Dietmann S, Murakami K, Down TA, Leitch HG, Surani MA (2013). Synergistic mechanisms of DNA demethylation during transition to ground-state pluripotency. Stem cell reports.

[265] Pastor WA, Aravind L, Rao A (2013). TETonic shift: biological roles of TET proteins in DNA demethylation and transcription. Nat Rev Mol Cell Biol.

[266] Wu SC, Zhang Y (2010). Active DNA demethylation: many roads lead to Rome. Nat Rev Mol Cell Biol.

[267] Xu GL, Wong J (2015). Oxidative DNA demethylation mediated by Tet enzymes. Natl Sci Rev.

[268] Hu X, Zhang L, Mao SQ, Li Z, Chen J, Zhang RR, Wu HP, Gao J, Guo F, Liu W, Xu GF (2014). Tet and TDG mediate DNA demethylation essential for mesenchymal-to-epithelial transition in somatic cell reprogramming. Cell Stem Cell.

[269] Bagci H, Fisher AG (2013). DNA demethylation in pluripotency and reprogramming: the role of tet proteins and cell division. Cell Stem Cell.

[270] Hahn MA, Szabó PE, Pfeifer GP (2014). 5-Hydroxymethylcytosine: a stable or transient DNA modification?. Genomics.

[271] Münzel M, Globisch D, Carell T (2011). 5-Hydroxymethylcytosine, the sixth base of the genome. Angew Chem Int Ed.

[272] Zeng C, Stroup EK, Zhang Z, Chiu BCH, Zhang W (2019). Towards precision medicine: advances in 5-hydroxymethylcytosine cancer biomarker discovery in liquid biopsy. Cancer Commun.

[273] Xu T, Gao H (2020). Hydroxymethylation and tumors: can 5-hydroxymethylation be used as a marker for tumor diagnosis and treatment?. Hum Genom.

[274] Nebbioso A, Tambaro FP, Dell’Aversana C, Altucci L (2018). Cancer epigenetics: moving forward. PLoS Genet.

[275] Taby R, Issa JPJ (2010). Cancer epigenetics. CA Cancer J Clin.

[276] Joosten SC, Smits KM, Aarts MJ, Melotte V, Koch A, Tjan-Heijnen VC, van Engeland M (2018). Epigenetics in renal cell cancer: mechanisms and clinical applications. Nat Rev Urol.

[277] Valdespino V, Valdespino PM (2015). Potential of epigenetic therapies in the management of solid tumors. Cancer Manag Res.

[278] Feng S, De Carvalho DD (2021). Clinical advances in targeting epigenetics for cancer therapy. FEBS J.

[279] Berdasco M, Esteller M (2019). Clinical epigenetics: seizing opportunities for translation. Nat Rev Genet.

[280] Lakshmaiah KC, Jacob LA, Aparna S, Lokanatha D, Saldanha SC (2014). Epigenetic therapy of cancer with histone deacetylase inhibitors. J Cancer Res Ther.

[281] Mani S, Herceg Z (2010). DNA demethylating agents and epigenetic therapy of cancer. Adv Genet.

[282] Yoo CB, Jones PA (2006). Epigenetic therapy of cancer: past, present, and future. Nat Rev Drug Discov.

[283] Zhu WG, Otterson GA (2003). The interaction of histone deacetylase inhibitors and DNA methyltransferase inhibitors in the treatment of human cancer cells. Curr Med Chem Anti-Cancer Agents.

[284] Ganesan A, Arimondo PB, Rots MG, Jeronimo C, Berdasco M (2019). The timeline of epigenetic drug discovery: from reality to dreams. Clin Epigenet.

[285] Ganai SA. Different groups of HDAC inhibitors based on various classifications. In: Histone deacetylase inhibitors—epidrugs for neurological disorders. Springer; 2019. p. 33–38. 10.1007/978-981-13-8019-8_5.

[286] O'Connor OA, Horwitz S, Masszi T, Van Hoof A, Brown P, Doorduijn J, Hess G, Jurczak W, Knoblauch P, Chawla S, Bhat G (2015). Belinostat in patients with relapsed or refractory peripheral T-cell lymphoma: results of the pivotal phase II BELIEF (CLN-19) study. J Clin Oncol.

[287] Lee HZ, Kwitkowski VE, Del Valle PL, Ricci MS, Saber H, Habtemariam BA, Bullock J, Bloomquist E, Shen YL, Chen XH, Brown J (2015). FDA approval: belinostat for the treatment of patients with relapsed or refractory peripheral T-cell lymphoma. Clin Cancer Res.

[288] Atadja P (2009). Development of the pan-DAC inhibitor panobinostat (LBH589): successes and challenges. Cancer Lett.

[289] TOCRIS.Cancer research product guide (2015).

[290] Costa-Pinheiro P, Montezuma D, Henrique R, Jerónimo C (2015). Diagnostic and prognostic epigenetic biomarkers in cancer. Epigenomics.

[291] Herceg Z, Hainaut P (2007). Genetic and epigenetic alterations as biomarkers for cancer detection, diagnosis, and prognosis. Mol Oncol.

[292] Ziegler A, Koch A, Krockenberger K, Großhennig A (2012). Personalized medicine using DNA biomarkers: a review. Hum Genet.

[293] Sharma A (2019). Hiding in plain sight: epigenetic plasticity in drug-induced tumor evolution. Epigenet Insights.

[294] Feng Z, Li J, Zhang JR, Zhang X (2014). qDNAmod: a statistical model-based tool to reveal intercellular heterogeneity of DNA modification from SMRT sequencing data. Nucleic Acids Res.

[295] Feng Z, Li J, Zhang JR, Zhang X (2015). qDNAmod: a statistical model-based tool to reveal intercellular heterogeneity of DNA modification from SMRT sequencing data. Nucleic Acids Res.

[296] Gao D, Herman JG, Guo M (2016). The clinical value of aberrant epigenetic changes of DNA damage repair genes in human cancer. Oncotarget.

[297] Feinberg AP, Ohlsson R, Henikoff S (2006). The epigenetic progenitor origin of human cancer. Nat Rev Genet.

[298] Martínez-Cardús A, Moran S, Musulen E, Moutinho C, Manzano JL, Martinez-Balibrea E, Tierno M, Élez E, Landolfi S, Lorden P, Arribas C (2016). Epigenetic homogeneity within colorectal tumors predicts shorter relapse-free and overall survival times for patients with locoregional cancer. Gastroenterology.

[299] Assenov Y, Brocks D, Gerhäuser C (2018). Intratumor heterogeneity in epigenetic patterns. Semin Cancer Biol.

[300] Shibue T, Weinberg RA (2017). EMT, CSCs, and drug resistance: the mechanistic link and clinical implications. Nat Rev Clin Oncol.

[301] Wang X, Zhang H, Chen X (2019). Drug resistance and combating drug resistance in cancer. Cancer Drug Resist.

[302] Marusyk A, Almendro V, Polyak K (2012). Intra-tumour heterogeneity: a looking glass for cancer?. Nat Rev Cancer.

[303] Liu C, Liu L, Chen X, Shen J, Shan J, Xu Y, Yang Z, Wu L, Xia F, Bie P, Cui Y (2013). Decrease of 5-hydroxymethylcytosine is associated with progression of hepatocellular carcinoma through downregulation of TET1. PLoS ONE.

[304] Liu J, Jiang J, Mo J, Liu D, Cao D, Wang H, He Y, Wang H (2019). Global DNA 5-hydroxymethylcytosine and 5-formylcytosine contents are decreased in the early stage of hepatocellular carcinoma. Hepatology.

[305] Wang P, Yan Y, Yu W, Zhang H (2019). Role of ten-eleven translocation proteins and 5-hydroxymethylcytosine in hepatocellular carcinoma. Cell Prolif.

[306] Jiang J, Yan T, Guo F (2021). Global DNA 5hmC and CK195hmC+ contents: a promising biomarker for predicting prognosis in small hepatocellular carcinoma. Curr Oncol.

[307] Song CX, Yin S, Ma L, Wheeler A, Chen Y, Zhang Y, Liu B, Xiong J, Zhang W, Hu J, Zhou Z (2017). 5-Hydroxymethylcytosine signatures in cell-free DNA provide information about tumor types and stages. Cell Res.

[308] Li W, Zhang X, Lu X, You L, Song Y, Luo Z, Zhang J, Nie J, Zheng W, Xu D, Wang Y (2017). 5-Hydroxymethylcytosine signatures in circulating cell-free DNA as diagnostic biomarkers for human cancers. Cell Res.

[309] Guler GD, Ning Y, Ku CJ, Phillips T, McCarthy E, Ellison CK, Bergamaschi A, Collin F, Lloyd P, Scott A, Antoine M (2020). Detection of early-stage pancreatic cancer using 5-hydroxymethylcytosine signatures in circulating cell-free DNA. Nat Commun.

[310] Xu S, Chen L, Shen Q, Yu H, Pei S, Yangting Z, He X, Wang Q, Li D (2021). 5-hydroxymethylcytosine signatures in circulating cell-free DNA as diagnostic biomarkers for late-onset Alzheimer's disease. J Alzheimer's Dis.

[311] Jankowska AM, Millward CL, Caldwell CW (2015). The potential of DNA modifications as biomarkers and therapeutic targets in oncology. Expert Rev Mol Diagn.

[312] Nestor C, Ruzov A, Meehan RR, Dunican DS (2010). Enzymatic approaches and bisulfite sequencing cannot distinguish between 5-methylcytosine and 5-hydroxymethylcytosine in DNA. Biotechniques.

[313] Huang YW, Huang THM, Wang LS (2010). Profiling DNA methylomes from microarray to genome-scale sequencing. Technol Cancer Res Treat.

[314] Zhao LY, Song J, Liu Y, Song CX, Yi C (2020). Mapping the epigenetic modifications of DNA and RNA. Protein Cell.

[315] Wang T, Loo CE, Kohli RM (2021). Enzymatic approaches for profiling cytosine methylation and hydroxymethylation. Mol Metab.

[316] Liu Y, Siejka-Zielińska P, Velikova G, Bi Y, Yuan F, Tomkova M, Bai C, Chen L, Schuster-Böckler B, Song CX (2019). Bisulfite-free direct detection of 5-methylcytosine and 5-hydroxymethylcytosine at base resolution. Nat Biotechnol.

[317] Cavalcante RG, Patil S, Park Y, Rozek LS, Sartor MA (2017). Integrating DNA methylation and hydroxymethylation data with the mint pipeline. Can Res.

[318] Cao M, Zhang C, Zhou L (2021). DNA methylation detection technology and plasma-based methylation biomarkers in screening of gastrointestinal carcinoma. Epigenomics.

[319] Clark SJ, Smallwood SA, Lee HJ, Krueger F, Reik W, Kelsey G (2017). Genome-wide base-resolution mapping of DNA methylation in single cells using single-cell bisulfite sequencing (scBS-seq). Nat Protoc.

[320] Liu Y, Hu Z, Cheng J, Siejka-Zielińska P, Chen J, Inoue M, Ahmed AA, Song CX (2021). Subtraction-free and bisulfite-free specific sequencing of 5-methylcytosine and its oxidized derivatives at base resolution. Nat Commun.

[321] Jurkowski TP (2020). Technologies, and applications for the assessment of 5-hydroxymethylcytosine. Epigenet Methods.

[322] Liu Z, Wang Z, Jia E, Ouyang T, Pan M, Lu J, Ge Q, Bai Y (2019). Analysis of genome-wide in cell-free DNA methylation: progress and prospect. Analyst.

[323] Booth MJ, Branco MR, Ficz G, Oxley D, Krueger F, Reik W, Balasubramanian S (2012). Quantitative sequencing of 5-methylcytosine and 5-hydroxymethylcytosine at single-base resolution. Science.

[324] Booth MJ, Ost TW, Beraldi D, Bell NM, Branco MR, Reik W, Balasubramanian S (2013). Oxidative bisulfite sequencing of 5-methylcytosine and 5-hydroxymethylcytosine. Nat Protoc.

[325] De Borre M., Branco MR. Oxidative bisulfite sequencing: an experimental and computational protocol. In: DNA modifications; 2021. p. 333–348. 10.1007/978-1-0716-0876-0_26. 10.1007/978-1-0716-0876-0_26 32822043

[326] Zeng H, He B, Xia B, Bai D, Lu X, Cai J, Chen L, Zhou A, Zhu C, Meng H, Gao Y (2018). Bisulfite-free, nanoscale analysis of 5-hydroxymethylcytosine at single-base resolution. J Am Chem Soc.

[327] Hu K, Ting AH, Li J (2015). BSPAT: a fast online tool for DNA methylation co-occurrence pattern analysis based on high-throughput bisulfite sequencing data. BMC Bioinform.

[328] Skvortsova K, Stirzaker C, Taberlay P (2019). The DNA methylation landscape in cancer. Essays Biochem.

[329] Booth MJ, Raiber EA, Balasubramanian S (2015). Chemical methods for decoding cytosine modifications in DNA. Chem Rev.

[330] Parle-Mcdermott A, Harrison A (2011). DNA methylation: a timeline of methods and applications. Front Genet.

[331] Feng L, Lou J. DNA methylation analysis. In: Nanotoxicity; 2019. p. 181–227. 10.1007/978-1-4939-8916-4_12.

[332] Dahl C, Guldberg P (2003). DNA methylation analysis techniques. Biogerontology.

[333] Kurdyukov S, Bullock M (2016). DNA methylation analysis: choosing the right method. Biology.

[334] Dietrich D, Uhl B, Sailer V, Holmes EE, Jung M, Meller S, Kristiansen G (2013). Improved PCR performance using template DNA from formalin-fixed and paraffin-embedded tissues by overcoming PCR inhibition. PLoS ONE.

[335] Grunau C, Clark SJ, Rosenthal A (2001). Bisulfite genomic sequencing: systematic investigation of critical experimental parameters. Nucleic Acids Res.

[336] Darst RP, Pardo CE, Ai L, Brown KD, Kladde MP (2010). Bisulfite sequencing of DNA. Curr Protoc Mol Biol.

[337] Portela A, Esteller M (2010). Epigenetic modifications and human disease. Nat Biotechnol.

[338] Esteller M (2007). Cancer epigenomics: DNA methylomes and histone-modification maps. Nat Rev Genet.

[339] Jones PA, Takai D (2001). The role of DNA methylation in mammalian epigenetics. Science.

[340] Singal R, Ginder GD (1999). DNA methylation. Blood J Am Soc Hematol.

[341] Richardson BC (2002). Role of DNA methylation in the regulation of cell function: autoimmunity, aging, and cancer. J Nutr.

[342] Luczak MW, Jagodziński PP (2006). The role of DNA methylation in cancer development. Folia Histochem Cytobiol.

[343] Lee PP, Fitzpatrick DR, Beard C, Jessup HK, Lehar S, Makar KW, Pérez-Melgosa M, Sweetser MT, Schlissel MS, Nguyen S, Cherry SR (2001). A critical role for Dnmt1 and DNA methylation in T cell development, function, and survival. Immunity.

[344] Gupta PK (2008). Single-molecule DNA sequencing technologies for future genomics research. Trends Biotechnol.

[345] van Dijk EL, Jaszczyszyn Y, Naquin D, Thermes C (2018). The third revolution in sequencing technology. Trends Genet.

[346] Lee I, Razaghi R, Gilpatrick T, Molnar M, Gershman A, Sadowski N, Sedlazeck FJ, Hansen KD, Simpson JT, Timp W (2020). Simultaneous profiling of chromatin accessibility and methylation on human cell lines with nanopore sequencing. Nat Methods.

[347] Henderson J, Salzberg S, Fasman KH (1997). Finding genes in DNA with a hidden Markov model. J Comput Biol.

[348] Rand AC, Jain M, Eizenga JM, Musselman-Brown A, Olsen HE, Akeson M, Paten B (2017). Mapping DNA methylation with high-throughput nanopore sequencing. Nat Methods.

[349] Simpson JT, Workman RE, Zuzarte PC, Matei DM, Dursi LJ, Timp W (2017). Detecting DNA cytosine methylation using nanopore sequencing. Nat Methods.

[350] Wu KJ (2020). The epigenetic roles of DNA N6-Methyladenine (6mA) modification in eukaryotes. Cancer Lett.

[351] Qiu H, Sarathy A, Schulten K, Leburton JP (2017). Detection and mapping of DNA methylation with 2D material nanopores. Mater Appl.

[352] Ameur A, Kloosterman WP, Hestand MS (2019). Single-molecule sequencing: towards clinical applications. Trends Biotechnol.

[353] Tyson JR, O'Neil NJ, Jain M, Olsen HE, Hieter P, Snutch TP (2018). MinION-based long-read sequencing, and assembly extends the Caenorhabditis elegans reference genome. Genome Res.

[354] Gershman A, Sauria ME, Hook PW, Hoyt SJ, Razaghi R, Koren S, Altemose N, Caldas GV, Vollger MR, Logsdon GA, Rhie A (2021). Epigenetic patterns in a complete human genome. bioRxiv.

[355] Minnoye L, Marinov GK, Krausgruber T, Pan L, Marand AP, Secchia S, Greenleaf WJ, Furlong EE, Zhao K, Schmitz RJ, Bock C (2021). Chromatin accessibility profiling methods. Nat Rev Methods Primers.

[356] Shen L, Waterland RA (2007). Methods of DNA methylation analysis. Curr Opin Clin Nutr Metab Care.

[357] Umer M, Herceg Z (2013). Deciphering the epigenetic code: an overview of DNA methylation analysis methods. Antioxid Redox Signal.

[358] Bibikova M, Le J, Barnes B, Saedinia-Melnyk S, Zhou L, Shen R, Gunderson KL (2009). Genome-wide DNA methylation profiling using Infinium® assay. Epigenomics.

[359] Sun XX, Yu Q (2015). Intra-tumor heterogeneity of cancer cells and its implications for cancer treatment. Acta Pharmacol Sin.

[360] Adusumalli S, Mohd Omar MF, Soong R, Benoukraf T (2015). Methodological aspects of whole-genome bisulfite sequencing analysis. Brief Bioinform.

[361] De Coster W, Weissensteiner MH, Sedlazeck FJ (2021). Towards population-scale long-read sequencing. Nat Rev Genet.

[362] Sun S, Noviski A, Yu X (2013). MethyQA: a pipeline for bisulfite-treated methylation sequencing quality assessment. BMC Bioinform.

[363] Liu L, Li Y, Li S, Hu N, He Y, Pong R, Lin D, Lu L, Law M (2012). Comparison of next-generation sequencing systems. J Biomed Biotechnol.

[364] Tang Y, Zheng SJ, Qi CB, Feng YQ, Yuan BF (2015). Sensitive and simultaneous determination of 5-methylcytosine and its oxidation products in genomic DNA by chemical derivatization coupled with liquid chromatography-tandem mass spectrometry analysis. Anal Chem.

[365] Shen L, Zhang Y (2013). 5-Hydroxymethylcytosine: generation, fate, and genomic distribution. Curr Opin Cell Biol.

[366] Du Y, Wang Y, Hu X, Liu J, Diao J (2020). Single-molecule quantification of 5-methylcytosine and 5-hydroxymethylcytosine in cancer genome. View.

[367] Rand AC, Jain M, Eizenga J, Musselman-Brown A, Olsen HE, Akeson M, Paten B (2016). Cytosine variant calling with high-throughput nanopore sequencing. bioRxiv.

[368] Jain M. High-coverage long read DNA sequencing with the Oxford Nanopore MinION. University of California; 2017. https://escholarship.org/uc/item/11p602ct.

[369] Kirk JM, Kim SO, Inoue K, Smola MJ, Lee DM, Schertzer MD, Wooten JS, Baker AR, Sprague D, Collins DW, Horning CR (2018). Functional classification of long non-coding RNAs by k-mer content. Nat Genet.

[370] Wang Y, Navin NE (2015). Advances, and applications of single-cell sequencing technologies. Mol Cell.

[371] Schwartzman O, Tanay A (2015). Single-cell epigenomics: techniques and emerging applications. Nat Rev Genet.

[372] Grønbaek K, Hother C, Jones PA (2007). Epigenetic changes in cancer. APMIS.

[373] Harris TJ, McCormick F (2010). The molecular pathology of cancer. Nat Rev Clin Oncol.

[374] Chatterjee N, Bivona TG (2019). Polytherapy and targeted cancer drug resistance. Trends Cancer.

[375] Davenport CF, Scheithauer T, Dunst A, Bahr FS, Dorda M, Wiehlmann L, Tran DDH (2021). Genome-wide methylation mapping using nanopore sequencing technology identifies novel tumor suppressor genes in hepatocellular carcinoma. Int J Mol Sci.

[376] Sakamoto Y, Sereewattanawoot S, Suzuki A (2020). A new era of long-read sequencing for cancer genomics. J Hum Genet.

[377] Zhuang J, Huo Q, Yang F, Xie N (2020). Perspectives on the role of histone modification in breast cancer progression and the advanced technological tools to study epigenetic determinants of metastasis. Front Genet.

[378] Nattestad M, Goodwin S, Ng K, Baslan T, Sedlazeck FJ, Rescheneder P, Garvin T, Fang H, Gurtowski J, Hutton E, Tseng E (2018). Complex rearrangements and oncogene amplifications revealed by long-read DNA and RNA sequencing of a breast cancer cell line. Genome Res.

[379] Minervini CF, Cumbo C, Orsini P, Anelli L, Zagaria A, Impera L, Coccaro N, Brunetti C, Minervini A, Carcieri P, Tota G (2017). Mutational analysis in BCR-ABL1 positive leukemia by deep sequencing based on nanopore MinION technology. Exp Mol Pathol.

[380] Tang AD, Soulette CM, van Baren MJ, Hart K, Hrabeta-Robinson E, Wu CJ, Brooks AN (2020). Full-length transcript characterization of SF3B1 mutation in chronic lymphocytic leukemia reveals downregulation of retained introns. Nat Commun.

[381] Xu X, Jia J, Guo M (2020). The most recent advances in the application of nano-structures/nano-materials for single-cell sampling. Front Chem.

[382] Kelsey G, Stegle O, Reik W (2017). Single-cell epigenomics: recording the past and predicting the future. Science.

[383] Trapnell C, Salzberg SL (2009). How to map billions of short reads onto genomes. Nat Biotechnol.

[384] Steinberg KM, Schneider VA, Alkan C (2017). Building and improving reference genome assemblies. Proc IEEE.

[385] Oxford Nanopore Technologies. 1Dsquared kit available in the store: boost accuracy, simple prep; 2017. https://nanoporetech.com/about-us/news/1d-squared-kit-available-store-boost-accuracy-simple-prep. Accessed 20 Apr 2018.

[386] Brown CG. Oxford Nanopore Technologies: GridION X5 the sequel. https://www.youtube.com/results?search_query=Oxford+Nanopore+Technologies%3A+GridION+X5+The+Sequel+. Streamed live March 2017. Accessed 29 May 2018.

[387] Cornelis S, Gansemans Y, Vander Plaetsen AS, Weymaere J, Willems S, Deforce D, Van Nieuwerburgh F (2019). Forensic tri-allelic SNP genotyping using nanopore sequencing. Forensic Sci Int Genet.

[388] Wang Y, Zhao Y, Bollas A, Wang Y, Au KF (2021). Nanopore sequencing technology, bioinformatics and applications. Nat Biotechnol.

[389] Wick RR, Judd LM, Holt KE (2019). Performance of neural network basecalling tools for Oxford Nanopore sequencing. Genome Biol.

[390] Amarasinghe SL, Su S, Dong X, Zappia L, Ritchie ME, Gouil Q (2020). Opportunities and challenges in long-read sequencing data analysis. Genome Biol.

[391] Vu T, Davidson SL, Borgesi J, Maksudul M, Jeon TJ, Shim J (2017). Piecing together the puzzle: nanopore technology in detection and quantification of cancer biomarkers. RSC Adv.

[392] Khoo BL, Chaudhuri PK, Ramalingam N, Tan DS, Lim CT, Warkiani ME (2016). Single-cell profiling approaches to probing tumor heterogeneity. Int J Cancer.

[393] Euskirchen P, Bielle F, Labreche K, Kloosterman WP, Rosenberg S, Daniau M (2017). Same-day genomic and epigenomic diagnosis of brain tumors using real-time nanopore sequencing. Acta Neuropathol.

[394] Zhou L, Qiu Q, Zhou Q, Li J, Yu M, Li K, Xu L, Ke X, Xu H, Lu B, Wang H (2022). Long-read sequencing unveils high-resolution HPV integration and its oncogenic progression in cervical cancer. Nat Commun.

[395] Knief C (2014). Analysis of plant microbe interactions in the era of next generation sequencing technologies. Front Plant Sci.

[396] Malla MA, Dubey A, Kumar A, Yadav S, Hashem A, Abd Allah EF (2019). Exploring the human microbiome: the potential future role of next-generation sequencing in disease diagnosis and treatment. Front Immunol.

[397] Ni P, Huang N, Nie F, Zhang J, Zhang Z, Wu B, Bai L, Liu W, Xiao CL, Luo F, Wang J (2021). Genome-wide detection of cytosine methylations in plant from Nanopore data using deep learning. Nat Commun.

[398] Liu Y, Rosikiewicz W, Pan Z, Jillette N, Wang P, Taghbalout A, Foox J, Mason C, Carroll M, Cheng A, Li S (2021). DNA methylation-calling tools for Oxford Nanopore sequencing: a survey and human epigenome-wide evaluation. Genome Biol.

[399] Kumar KR, Cowley MJ, Davis RL (2019). Next-generation sequencing and emerging technologies. Semin Thromb Hemost.

[400] Ni P, Huang N, Zhang Z, Wang DP, Liang F, Miao Y, Xiao CL, Luo F, Wang J (2019). DeepSignal: detecting DNA methylation state from Nanopore sequencing reads using deep-learning. Bioinformatics.

[401] Fujimoto A, Wong JH, Yoshii Y, Akiyama S, Tanaka A, Yagi H, Shigemizu D, Nakagawa H, Mizokami M, Shimada M (2021). Whole-genome sequencing with long reads reveals complex structure and origin of structural variation in human genetic variations and somatic mutations in cancer. Genome Med.

[402] Wongsurawat T, Jenjaroenpun P, De Loose A, Alkam D, Ussery DW, Nookaew I, Leung YK, Ho SM, Day JD, Rodriguez A (2020). A novel Cas9-targeted long-read assay for simultaneous detection of IDH1/2 mutations and clinically relevant MGMT methylation in fresh biopsies of diffuse glioma. Acta Neuropathol Commun.

[403] Gilpatrick T, Lee I, Graham JE, Raimondeau E, Bowen R, Heron A, Downs B, Sukumar S, Sedlazeck FJ, Timp W (2020). Targeted nanopore sequencing with Cas9-guided adapter ligation. Nat Biotechnol.

[404] Gabrieli T, Sharim H, Fridman D, Arbib N, Michaeli Y, Ebenstein Y (2018). Selective nanopore sequencing of human BRCA1 by Cas9-assisted targeting of chromosome segments (CATCH). Nucleic Acids Res.

[405] Ewing AD, Smits N, Sanchez-Luque FJ, Faivre J, Brennan PM, Richardson SR, Cheetham SW, Faulkner GJ (2020). Nanopore sequencing enables comprehensive transposable element epigenomic profiling. Mol Cell.

[406] Djirackor L, Halldorsson S, Niehusmann P, Leske H, Capper D, Kuschel LP, Pahnke J, Due-Tønnessen BJ, Langmoen IA, Sandberg CJ, Euskirchen P (2021). Intraoperative DNA methylation classification of brain tumors impacts neurosurgical strategy. Neuro Oncol Adv.

